# Heteroatom‐Synergistic Effect on Anchoring Polysulfides In Chalcone‐Linked Nanographene Covalent Organic Frameworks for High‐Performance Li─S Batteries

**DOI:** 10.1002/advs.202415897

**Published:** 2025-02-25

**Authors:** Kayaramkodath Chandran Ranjeesh, Bharathkumar H. Javaregowda, Safa Gaber, Preeti Bhauriyal, Sushil Kumar, Tina Skorjanc, Matjaž Finšgar, Thomas Heine, Kothandam Krishnamoorthy, Dinesh Shetty

**Affiliations:** ^1^ Department of Chemistry Khalifa University of Science & Technology Abu Dhabi P.O. Box 127788 UAE; ^2^ Polymer Science and Engineering Division, CSIR‐National Chemical Laboratory (CSIR‐NCL) Pune 411008 India; ^3^ Faculty of Chemistry and Food Chemistry Technische Universität Dresden 01062 Dresden Germany; ^4^ Materials Research Laboratory University of Nova Gorica Vipavska cesta 11c Ajdovscina 5270 Slovenia; ^5^ Faculty of Chemistry and Chemical Engineering University of Maribor Smetanova ulica 17 Maribor 2000 Slovenia; ^6^ Helmholtz‐Zentrum Dresden‐Rossendorf Center for Advanced Systems Understanding, CASUS Untermarkt 20 02826 Görlitz Germany; ^7^ Department of Chemistry and ibs for Nanomedicine Yonsei University Seodaemun‐gu Seoul 120‐749 South Korea; ^8^ Center for Catalysis & Separations (CeCaS) Khalifa University Science & Technology Abu Dhabi P.O. Box 127788 UAE

**Keywords:** covalent organic frameworks, lithium‐sulfur battery, nanographene, anion‐pi interaction, polysulfide shuttle

## Abstract

Lithium‐sulfur (Li─S) batteries are an attractive option for future energy storage devices because they offer higher theoretical specific capacity, energy density, and cost‐effectiveness than commercial lithium‐ion batteries. However, the practical applications of Li─S batteries are significantly limited by the shuttle effect caused by intermediate lithium polysulfides (LiPSs) and slow redox kinetics. In this study, the molecular engineering of chalcone‐linked, sp^2^‐bonded nanographene‐type covalent organic frameworks (COFs) as sulfur hosts is reported to enhance interactions with LiPSs, thereby effectively suppressing the shuttle effect. The developed sulfur‐hosting cathode material demonstrated outstanding battery performance, surpassing most reported materials by achieving a specific capacity of 1228 mA h g^−1^ at 0.5C, with 80% retention after 500 cycles and an average Coulombic Efficiency (C.E.) of 99%. Additionally, the mechanisms of sulfur immobilization, the subsequent conversion into lithium polysulfides (LiPSs), and their binding energies with COFs are investigated using density functional theory (DFT) calculations. These findings offer valuable insights into the structure‐property relationships essential for developing more efficient sulfur‐hosting cathodes.

## Graphical abstract

A series of nanographene‐type covalent organic framework sulfur‐hosting cathodes are developed by introducing pyridine and chalcone functionalities to amend strong lithium polysulfides (LiPSs) interactions to suppress the shuttle effect in lithium‐sulfur (Li─S) batteries. The cathode material showed a specific capacity of 1228 mA h g^−1^ at 0.5C with 80% retention after 500 cycles and maintained an average Coulombic Efficiency (C.E.) of 99%.

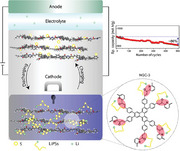

## 1 Introduction

The rapid evolution of modern electronics and electric vehicles has motivated scientists to develop safer rechargeable batteries with greater capacity and lower costs.^[^ ^1^ ^]^ Currently, lithium‐ion batteries (LIBs) with lithium‐mixed multi‐metal oxide cathodes possessing limited capacity (≈270 mA h g^−1^) and energy density (≈400 Wh kg^−1^) are powering portable electronic devices and electric vehicles. Additionally, the metals used in these cathodes, such as cobalt in LiCoO₂, are costly and often sourced from mines with harsh labor conditions. In this context, rechargeable lithium‐sulfur (Li─S) batteries are a promising candidate for next‐generation energy storage due to their high theoretical gravimetric capacity (1675 mA h g⁻¹) and energy density compared to state‐of‐the‐art lithium‐ion batteries (2600 vs 400 Wh kg^−1^).^[^ ^2^, ^3^ ^]^ Furthermore, Li─S batteries eliminate the need for cobalt in the electrodes, making them a viable option for future energy storage devices. Additionally, the natural abundance of sulfur, a major byproduct of petroleum refineries, contributes to their affordability.^[^ ^4^, ^5^ ^]^ Nevertheless, despite extensive research on Li─S batteries, their performance remains significantly below theoretical potential^[^ ^6^ ^]^ due to several factors:1) extremely poor conductivity for both electrons and ions resulting from the intrinsic insulating nature of sulfur (5 × 10^−30^ S cm^−1^) and its discharged (Li_2_S) (3 × 10^−7^ S cm^−1^) species, 2) sluggish conversion of sulfur to polysulfides, 3) loss of sulfur in each charge‐discharge cycle and quick capacity fading by polysulfide shuttle effect, 4) active site blocking by the insoluble and electrochemically inactive discharge products (Li_2_S and Li_2_S_2_) which leads to poor Coulombic Efficiency (C.E.) and poor cycle life, 5) a significant volumetric expansion (up to 80%) of cathode materials during discharge due to the density difference between S_8_ (2.03 g cm^−3^) and Li_2_S (1.63 g cm^−3^), and 6) the loss of active materials because of the uneven dissolution and deposition of S_8_ and Li_2_S within the electrode.^[^ ^6^, ^7^ ^]^

**Figure 1:**
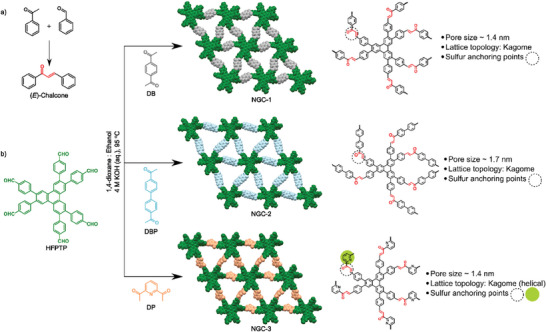
a) The Claisen‐Schmidt condensation for the preparation of the molecular analog, (E)‐Chalcone. b) Schematic illustration of the **NGC‐1,2,3** synthesis highlighting each material's rationally designed sulfur‐hosting features. The sulfur anchoring points are highlighted. Dashed: chalcone‐based, green: pyridine‐based.

To solve these problems, efforts have been made to develop advanced porous carbonaceous sulfur host materials to physically or chemically encase sulfur, improve conductivity, and immobilize LiPSs.^[^ ^8^, ^9^, ^10^ ^]^ Among these approaches, using a high‐caliber sulfur‐hosting cathode was the most effective approach to suppress the polysulfide shuttle, thereby improving the overall performance of Li─S batteries.^[^ ^6^, ^7^, ^8^, ^9^, ^10^ ^]^ A recent attractive addition to the sulfur hosting material family is 2D covalent organic frameworks (2D‐COFs), a class of covalent crystalline porous polymers with tunable nanopores and made of lightweight elements.^[^ ^11^, ^12^, ^13^, ^14^, ^15^, ^16^ ^]^ Notably, stacking 2D planar sheets in 2D‐COFs can enhance sulfur impregnation, thereby preventing sulfur aggregation and reducing soluble LiPSs intermediates' diffusive loss. However, most reported COFs primarily confine LiPSs within their micro‐ or mesopores through weak physical confinement/interactions. The lack of strong and effective binding sites in the vast majority of current reports,^[^ ^17^, ^18^, ^19^, ^20^, ^21^ ^]^ modest redox activity, and electronically insulating characteristics of COFs significantly limit their ability to suppress LiPSs shuttling.^[^ ^22^, ^23^, ^24^, ^25^, ^26^, ^27^ ^]^ Therefore, rationally designed COF‐based sulfur hosts with an appropriate pore environment and chemical structure are needed to reduce the shuttle effect and improve the battery performance.^[^ ^28^, ^29^, ^30^, ^31^, ^32^, ^33^, ^34^, ^35^, ^36^, ^37^ ^]^ In this context, herein, we rationally designed chalcone‐linked, sp^2^‐bonded nanographene‐type COFs (**Figure** **1**; **NGC‐1**, **NGC‐2**, and **NGC‐3)** with a polyaromatic core connected to benzene, extended biphenyl, or pyridine units to troubleshoot the existing limitations of sulfur hosting cathode materials. The designed **NGC**s possess a dual confinement effect for sulfur and polysulfides. The COF topologies are chosen to provide a microporous network to physically confine LiPSs using 1) van der Waals forces that arise between polarized LiPSs and clouds of aromatic core and 2) the chalcone unit exhibits strong electrostatic interactions with the polar LiPSs while the pyridinic nitrogen immobilizes the LiPSs via Lewis acid‐base interactions thus suppressing their dissolution and shuttling.^[^ ^38^, ^39^ ^]^ Also, the large *π*‐surface of the polyaromatic core possesses anion‐*π* interactions with negatively charged LiPSs. Furthermore, the carbonyl‐rich COF backbone imparts inherited redox activity, further enhanced by the pyridine units in **NGC‐3**. Chalcone and pyridine units promote favorable sulfur hosting, aiding in the nucleation and deposition of Li_2_S on the electrode surface and, in turn, also enhancing their conversion kinetics. The conjugated backbone possesses sufficient electronic conductivity, facilitating the activation of insulating Li_2_S through charge transfer, thus enhancing sulfur utilization.^[^ ^38^ **^,^** ^39^ ^]^ Notably, polar carbonyl and pyridine groups on the quasi‐nano graphene core render these COFs amphiphilic, thus offering strong Li_2_S_x_ coordination, simultaneously facilitating their fast conversion and helping better surface coating for electrode preparation. The systemic molecular engineering within rationally designed COFs resulted in the listed desirable properties and, in turn, showed exceptional Li─S battery performance: the cathode‐specific capacity of 1073, 907, and 1228 mA h g^−1^ at 0.5C was achieved for **S/NGC‐1**, **S/NGC‐2**, and **S/NGC‐3** cathodes, respectively. Moreover, ≈80%, 75%, and 63% of their initial capacities after 500 cycles were retained, while maintaining an average C.E. of 99%. Moreover, at a higher C  rate of 1C, the pyridine‐functionalized **S/NGC‐3** cathode delivered an exceptionally high capacity (1032 mA h g^−1^) and retained 81% of its initial capacity. Notably, chalcone‐linked COFs showed a high sulfur content of 87 wt.%. Even at a high sulfur loading and under lean electrolyte conditions, **S/NGC‐3** displayed a very high specific capacity of 800 mA h g^−1^ at 0.25C  and maintained a stable performance over 100 cycles with negligible capacity loss. We have also performed the density functional theory (DFT) calculations to investigate the mechanism of sulfur immobilization, further conversion into LiPSs, and the binding energies of polysulfides into COFs. These findings shed light on the power of molecular‐level material design to unravel the structure and property relationship in the performance enhancement of sulfur‐hosting cathodes for Li─S batteries.

## 2 Results and Discussion

### 2.1 Synthesis and Material Characterizations

The chalcone is an extensively studied simple scaffold found in many natural products.^[^ ^40^ ^]^ However, chalcone‐linkage was recently introduced in COF‐synthesis.^[^ ^41^, ^42^ ^]^ To confirm the chalcone formation from the aryl aldehyde and aryl ketone by base‐catalyzed Claisen‐Schmidt reaction, we successfully synthesized a model derivative, *E*‐chalcone, in good yield (Figure 1a; Scheme S1, Supporting Information). Furthermore, nanographene‐type chalcone‐linked COFs (**NGC‐1, NGC‐2,** and **NGC‐3**) were synthesized via topology‐guided Claisen‐Schmidt polycondensation by reacting C_3_‐symmetric 2,3,6,7,10,11‐hexakis(4‐formylphenyl)triphenylene (**HFPTP**) with one of C_2_‐symmetric 1,4‐Diacetylbenzene (**DB**), 4,4′‐Diacetylbiphenyl (**DBP**), and 2,6‐Diacetylpyridine (**DP**) linkers under solvothermal conditions (Figure 1b; Schemes S2–S4, Supporting Information details in ESI). We extensively screened the synthetic conditions to optimize the crystallinity of resulting COFs (Table S1, Supporting Information).

**Figure 2:**
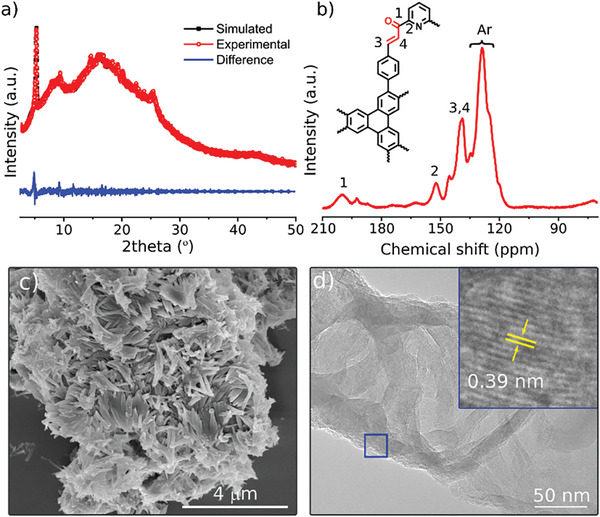
a) Experimental and simulated PXRD profiles of **NGC‐3**; simulated pattern for an ABC stacking mode is provided for comparison. b) ^13^C CP‐MAS NMR spectrum of **NGC‐3**. c) SEM image of **NGC‐3**. d) TEM image of the **NGC‐3**, showing layered sheets and lattice fringes (inset).

The crystallinity of the COFs was analyzed using powder X‐ray diffraction (PXRD). The intense and sharp diffraction peaks observed at low angles confirmed the long‐range ordered framework structures of **NGC‐1**, **NGC‐2**, and **NGC‐3** (**Figure** **2**a; Figures S1–S9, Supporting Information). The distinctive low‐angle 2θ peak detected at 5.65°, 5.58°, and 5.53° for **NGC‐1**, **NGC‐2,** and **NGC‐3**, respectively, corresponds to the (111) reflection plane. In comparison, the (001) plane associated with *π–π* stacking is identified at 23.34°, 23.16°, and 20.13° for the respective COFs (Figure 2a; Figures S4–S9, Supporting Information). To elucidate the structures of the three COFs, density functional‐based tight binding (DFTB) simulations were conducted using the Amsterdam Modeling Suite (AMS) ADF 2023.^[^ ^44^, ^45^ ^]^ We investigated various stacking models (Figures S1–S3, Supporting Information), and staggered ABC stacking structures were found to correlate best with experimental PXRD patterns.^[^ ^41^, ^43^ ^]^ The observed ABC packing can be attributed to the significant dipole moment exerted by the heteroatoms, specifically the oxygen atoms, within the molecular structure. This arrangement prevents the direct stacking of these polarized charged atoms on top of one another.^[^ ^41^ ^]^ The Pawley refinement has low residual values (respective R_wp_ and R_p_ of 2.30% and 1.67% for **NGC‐1**, 3.66% and 1.50% for **NGC‐2**, and 2.03% and 1.34% for **NGC‐3**), justifying the lattice parameters of ABC stacked COFs (Figures S4–S9 and Tables S2–S4, Supporting Information).^[^ ^41^ ^]^ ABC stacking mode in the **NGCs** offers distinct advantages, including enhanced exposure of functional sites (chalcone, pyridine, polyaromatic core) for stronger chemical interactions with lithium polysulfides, efficient physical encapsulation within its microporous architecture, and improved charge transport through reduced interlayer resistance. This configuration is also expected to prevent polysulfide aggregation, promoting uniform distribution that can improve cycling stability, making it highly effective for polysulfide capture and redox management.^[^ ^41^, ^42^ ^]^ The molecular structures of synthesized **COF**s were confirmed using Fourier‐transform infrared (FT‐IR) spectroscopy. The FT‐IR spectrum of **NGC‐1** shows a characteristic stretching mode vibration peak of C═O at ca. 1675 cm^−1^, derived from the **DB** carbonyl bond (C═O stretching mode at ca. 1681 cm^−1^). A peak attributed to a C═C bond appears at 1592 cm^−1^, merging with the remaining C═C stretching vibration mode, resulting in a broadened peak. Similar characteristic peaks that correspond to an allyl ketone (C═C─C═O) structure were observed for **NGC‐2** (C═O stretching mode at ca. 1678 cm^−1^, C═C at 1599 cm^−1^) and **NGC‐3** (C═O str. vibration at ca. 1675 cm^−1^, C═C at 1601 cm^−1^), indicating the successful Claisen‐Schmidt condensation and formation of chalcone linkage. The disappearance of the stretching mode of the vibration peak corresponding to the aromatic aldehyde group of **HFPTP** (Ar‐CHO; ≈1699 cm^−1^) suggests complete consumption of the starting material. The appearance of the bending mode of vibration peak for *trans* ─C═C─H at ≈975 cm^−1^ in the COFs signifies the predominant formation of a *trans*‐ geometry in the vinylene linkage, whereas a minor peak observed at ≈740 cm^−1^ can be attributed to the formation of *cis*‐ bonds (Figures S10–S12, Supporting Information). These observations indicate stereoselectivity, while there is a thermodynamic preference for *trans*‐chalcone formation.^[^ ^40^, ^41^, ^42^, ^43^ ^]^

Furthermore, solid‐state carbon‐13 cross‐polarization magic‐angle spinning nuclear magnetic resonance (^13^C CP MAS NMR) spectra of **NGC‐1**, **NGC‐2**, and **NGC‐3** displayed characteristic peaks corresponding to their chemical composition (Figure 2b; Figure S13, Supporting Information). The carbon atoms in the ketone groups (chalcone ─C═O) resulted in a peak at ≈192 ppm, and the newly formed olefin groups were responsible for a well‐resolved peak at ≈145 ppm (chalcone ─C═C─). The olefin‐linked phenyl carbon (─C═C─Ar) gave rise to a signal at ≈137 ppm.  The broad peak range between 133 and 120 ppm (Ar─ aromatic carbons) corresponds to aromatic carbon atoms within the structure. In addition, the characteristic signal for pyridine carbon atoms in **NGC‐3** was observed at ≈154 ppm (─C═N─). These NMR spectra provide conclusive evidence for successfully forming chalcone‐linked COFs. The morphologies of COFs were characterized by scanning electron microscopy (SEM) and transmission electron microscopy (TEM). SEM images of these COFs (Figures S14–S16, Supporting Information; Figure 2c) revealed the bulk morphologies of stacked layered sheets, whereas TEM images (Figure 2d; Figures S17–S19, Supporting Information) further confirm the multilayer stacks of 2D COFs. The nanodomain crystallinity of COFs is evidenced by the observation of distinct lattice fringes in the high‐resolution (HR)‐TEM images [(**NGC‐1**: *d* = 0.38 nm, (Figure S20a–c); **NGC‐2**: *d* = 0.36 nm, (Figure S20d–f) **NGC‐3**: *d* = 0.39 nm, (Figure 2d; Figure S20, Supporting Information)], which corresponds to *π–π* stacking distances between COF layers and matching with d‐spacing measurement between the 001 planes. SEM energy dispersive X‐ray (EDX) mapping of selected area images revealed the elemental composition of these COFs, which consisted of a uniform distribution of carbon and oxygen atoms throughout the framework structure (Figures S21 and S22, Supporting Information). Notably, the mapping of **NGC‐3** images showed a uniform distribution of nitrogen atoms originating from pyridine groups (Figure S23, Supporting Information).

The surface area and permanent porosity of **NGC‐COFs** were evaluated by measuring N_2_ adsorption isotherms at 77 K. The isotherms showed an increase in the lower relative pressure region (P/P_o_ = 0–0.05), corresponding to the microporous structures of the COFs (Figure S24, Supporting Information).^[^ ^46^, ^47^, ^48^ ^]^ The Brunauer–Emmett–Teller (BET) surface areas of the activated **NGC‐1**, **2**, and **3** samples were 172, 150, and 109 m^2^ g^−1^, respectively. The moderate BET surface areas could result from the ABC packing mode that blocks the regular triangular micropores.^[^ ^41^, ^42^, ^43^ ^]^ It matches the theoretical surface area trends of **NGC**s, calculated using PoreBlazer.^[^ ^48^ ^]^ The non‐local density functional theory (NLDFT) pore size distribution profile showed a narrow distribution centered at 1.4, 1.7, and 1.4 nm for **NGC‐1, 2,** and **3**, respectively (Figure S25, Supporting Information). The thermogravimetric analysis (TGA) indicated excellent thermal stability up to 300 °C (Figure S27, Supporting Information).

X‐ray photoelectron spectroscopy (XPS) analysis of **these COFs** showed the presence of the peaks at binding energies (BE) corresponding to C 1s for ─C═O (≈287 eV) and ─C═C─ (284.5 eV) along with the peaks corresponding to O 1s for ─C─OH (≈534 eV; less intense peak from incomplete condensation) and ─C═O (≈532 eV; high‐intensity peak). Furthermore, the XPS analysis for **NGC‐3** showed an additional peak at 399.2 eV corresponding to N 1s (─C═N, originating from the pyridine ring) (Figures S28 and S29, Supporting Information). The solid‐state ultraviolet/visible diffuse reflectance spectroscopy (UV/VIS DRS) of **NGC‐1**, **2**, and **3** displayed broad absorption spectra with absorption peaks extending up to 550, 560, and 590 nm, respectively (Figure S30a, Supporting Information). Compared to **NGC‐1**, the absorption peaks for **NGC‐2** and **NGC‐3** were red‐shifted by 10 and 40 nm, respectively, indicating the extended 𝜋‐conjugated 2D structures. The bandgap estimations by using the Tauc plot method imply the semiconducting nature of these COFs with the optical bandgaps of 2.75, 2.71, and 2.66 eV for **NGC‐1**, **2**, and **3**, respectively (Figure S30b, Supporting Information).

### 2.2 Electrochemical Performance of Chalcone‐Linked COFs as Sulfur Hosts in Li─S Battery

**Figure 3:**
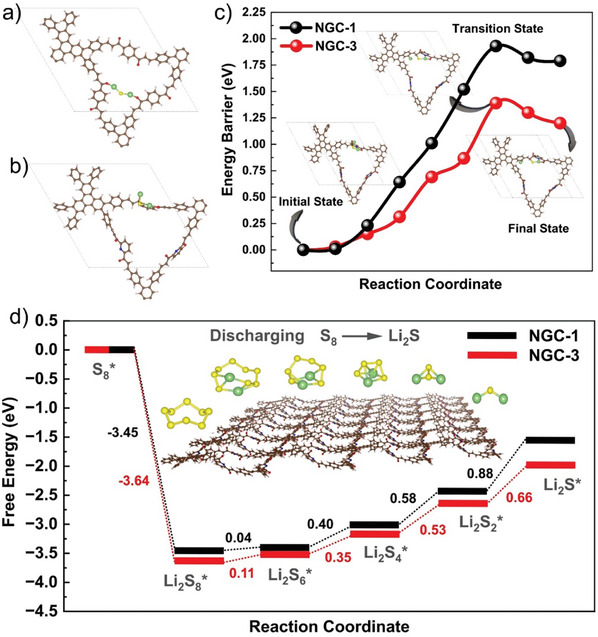
Stable binding configurations of Li_2_S on a) **NGC‐1** and b) **NGC‐3**. c) Li_2_S decomposition energy barriers for the charging process on **NGC‐1** and **NGC‐3**. Inset figures are top‐view schematic representations of the initial, transition, and final state configurations on **NGC‐3**. d) Gibbs free energy profiles for the discharging process.

**Figure 4:**
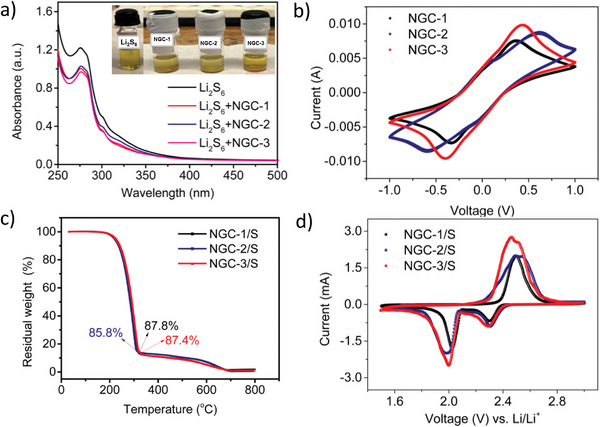
a) UV–vis. absorption spectra and images of Li_2_S_6_ solution before and after adsorption by **NGC**s. b) CVs of **NGC**s in symmetric cell configuration with Li_2_S_6_ electrolyte at 10.0 mV s^−1^. c) TGA profile of sulfur‐loaded **NGC**s d) CVs of Li‐S batteries with **NGC/S cathode**s at 0.2 mV s^−1^.

The potential efficacy of **NGC**s as cathode materials for Li─S batteries was assessed by polysulfide adsorption experiments. The polysulfide adsorption experiments were conducted on **NGC**s using Li_2_S_6_. Upon adding **NGC**s into the Li_2_S_6_ solution, the solution displayed a reduced color intensity, indicating a strong polysulfide adsorption ability of **NGC**s. To confirm this, UV–vis. absorption spectra of Li_2_S_6_ were recorded after 12 h. The Li_2_S_6_ solution showed two characteristic peaks at 280 and 300 nm corresponding to S_6_^2−^. Upon the addition of the COFs, the intensity of these peaks was significantly reduced. **NGC‐3**+Li_2_S_6_ showed the most prominent decrease, followed by **NGC‐1**+Li_2_S_6_ and **NGC‐2**+Li_2_S_6_ (**Figures** **3** and **4**a). **NGC‐3** possesses strong polysulfide adsorption due to the attractive interaction between the pyridinic nitrogens that are Lewis bases and Lewis acidic LiPSs. The larger pores of **NGC‐2** facilitate good encapsulation of polysulfides despite not having the strong interactions that existed in **NGC‐3**. The polysulfide adsorption in the case of **NGC‐1** is relatively better than **NGC‐2** because of the relatively smaller pores in **NGC‐1** compared to **NGC‐2**.

To confirm the strong polysulfide adsorption behavior of **NGC**s, DFT calculations were performed (Figure 3; Figures S31–S39, Supporting Information). We strategically investigated the binding behavior of Li₂S₈ and Li₂S clusters formed during the initial and final stages of the Li─S battery discharge cycle. The binding energy calculations (Table S6, Supporting Information) indicate that the **NGC‐3** exhibits strong binding with Li_2_S and Li_2_S_8_, with binding energies of −3.42 and −2.71 eV, respectively. These values are significantly higher than the binding energies of **NGC‐1** (−2.44 eV for Li_2_S and −2.01 eV for Li_2_S_8_) and **NGC‐2** (−2.34 eV for Li_2_S and −1.74 eV for Li_2_S_8_). The enhanced binding strength of **NGC‐3** is attributed to the active involvement of pyridine nitrogen in addition to the two carbonyl oxygen sites, which are the primary anchoring sites in **NGC‐1** and **NGC‐2** (Figure 3a,b). The charge density difference analysis revealed significant charge accumulation (yellow isosurface in Figure S35, Supporting Information) in the Li─O and Li─N interaction regions of **NGC‐3**, indicating the formation of Li─O and Li─N bonds. Furthermore, **NGC‐3** exhibits a notably larger isosurface than **NGC‐1** and **NGC‐2**, as shown in Figures S32 and S33 (Supporting Information), suggesting enhanced charge transfer between the active sites and lithium polysulfides. This enhanced charge transfer results in stronger binding interactions in **NGC‐3,** as evidenced by a significant elongation of the average Li─S bond lengths (∆r ≈0.20 Å) in both Li_2_S and Li_2_S_8_ upon binding to **NGC‐3** (Table S6, Supporting Information), compared to the smaller variation observed in **NGC‐1** and **NGC‐2** (∆r ≈0.12–0.14 Å). Bader charge analysis further supports these results, revealing that ≈0.84  and 0.34 ∣e∣ charges are transferred from Li_2_S and Li_2_S_8_ to **NGC‐3**, respectively. These values are higher than the corresponding charge transfers observed for **NGC‐1** (0.44  and 0.10 ∣e∣) and **NGC‐2** (0.44  and 0.07 ∣e∣), further confirming the superior binding capability of **NGC‐3** with lithium polysulfides. Therefore, we can conclude that all three **NGCs** possess the ability to both physically encapsulate LiPSs and establish robust chemical interactions, effectively suppressing LiPS shuttling.

To probe the electrocatalytic activity of **NGC**s toward lithium polysulfides, symmetric cells were fabricated using two identical electrodes of **NGC‐1/NGC‐2/NGC‐3** with Li_2_S_6_ electrolyte. The cyclic voltammetry (CV) plot of **NGC‐3** showed redox peaks at ±0.41 V, corresponding to the reversible conversion of Li_2_S_6_ to Li_2_S and further to S_8_ (Figure 4b). In contrast, **NGC‐1** and **NGC‐2** displayed redox peaks at ±0.34 and ±0.58 V, respectively (Figure 4b) for the same reversible conversion of Li_2_S_6_ to Li_2_S and further to S_8_. Notably, **NGC‐3** exhibits higher peak current values (9.8 mA) compared to **NGC‐2** (8.6 mA) and **NGC‐1** (7.5 mA), highlighting that the redox conversion kinetics of liquid polysulfides on **NGC‐3** was effectively accelerated, which is consistent with the low charge transfer resistance (R_ct_) depicted in the electrochemical impedance spectroscopy (EIS) spectra (Figure 6a).

To get deeper insights into the catalytic behavior of **NGC**s on polysulfide conversion, a Li_2_S nucleation and dissolution experiment was carried out. The nucleation of Li_2_S was probed by assembling the coin cells with **NGC** cathodes and Li foil anode with 20 µL Li_2_S_8_ catholyte and 20 µL LiTFSI as anolyte. The cells were first discharged to 2.06V at 0.1C and then the Li_2_S precipitation current at 2.05 V was recorded until the current dropped below 10^−5^ A. Figure S41 (Supporting Information) represents the Li_2_S nucleation and growth plot where the initial monotonically decreasing current represents the reduction of higher order LiPSs to Li_2_S_4_ and the following current peak represents the nucleation and growth of Li_2_S.^[^ ^6^, ^7^, ^8^, ^9^ ^]^ For Li_2_S dissolution, the cells were completely discharged at 0.1C. Then, the Li_2_S dissolution current was measured at 2.4 V until the current dropped below 10^−5^ A, resulting in the complete oxidation of Li_2_S to soluble polysulfides, as shown in Figure S42 (Supporting Information). The Li_2_S deposition and dissolution capacities were calculated by integrating the area under the current versus time plot. The Li_2_S precipitation capacity for **NGC‐1**, **NGC‐2**, and **NGC‐3** were calculated to be 244, 215, and 261 mAh g^−1^, while the Li_2_S dissolution capacities were 465.15, 429.4, and 504.44 mAh g^−1^, respectively. This suggests that **NGC‐3** possesses faster and better catalytic activity for Li_2_S conversion to soluble polysulfides, followed by **NGC‐1** and **NGC‐2**. This is attributed to the presence of pyridine N and carbonyl moieties, and it is well‐known in the literature that these functional groups facilitate the polysulfide conversion kinetics.^[^ ^37^, ^38^ ^]^

In the next set of experiments, Li─S batteries were fabricated using sulfur‐loaded **NGC**s. Sulfur distribution in **NGC**s is anticipated to be homogeneous due to abundant micropores and polar functionalities. The sulfur loading on the **NGC**s was quantified by TGA and found to be 87.8%, 85.4%, and 87.4% for **NGC‐1**, **NGC‐2**, and **NGC‐3,** respectively (Figure 4c). Upon sulfur loading, the surface areas were significantly reduced to 61 m^2^ g⁻¹ for **NGC‐1** **/S**, 29 m^2^ g⁻¹ for **NGC‐2** **/S**, and 26 m^2^ g⁻¹ for **NGC‐3** **/S**. This reduction in surface area is further supported by the disappearance of the microporous pore size distributions, previously centered ≈1.4, 1.7, and 1.3 nm for **NGC‐1**, **NGC‐2**, and **NGC‐3**, respectively (Figure S26, Supporting Information). These observations suggest that sulfur has effectively filled the micropores, reducing available surface area and confirming successful pore incorporation during the composite formation.^7^ The pore filling of sulfur alters the **NGC**’s physical characteristics and could enhance the electrochemical performance (Table S5, Supporting Information).

The Li─S batteries were fabricated using sulfur‐loaded **NGC** cathodes against Li as counter and reference electrode. The cyclic voltammograms of **NGC/S** cathodes are shown in Figure 4d. **NGC‐3** **/S** cathodes showed two typical cathodic peaks at ≈2.3 and 2.0 V related to the conversion of S_8_ to higher order soluble polysulfides Li_2_S_y_ (8>y>4) and further reduction to Li_2_S_2_/Li_2_S, respectively.^[^ ^49^, ^50^, ^51^ ^]^ In the anodic scan, the peaks at 2.48 and 2.51 V correspond to the conversion of solid Li_2_S/Li_2_S_2_ to Li_2_S_8_ and further to S8.^[^ ^49^, ^50^, ^51^ ^]^ **NGC‐3/S** showed higher redox peak currents and low onset oxidation and reduction potential values compared to **NGC‐1/S** and **NGC‐2/S**. This indicates faster polysulfide redox kinetics and effective sulfur utilization in **NGC‐3** and can be attributed to the micropores and presence of abundant pyridine N and carbonyl functional groups.^[^ ^35^, ^36^, ^37^, ^38^, ^39^ ^]^ These features of **NGC‐3** make it an ideal host where the system provides a strong electronic environment to anchor sulfur and lithium polysulfides. Thus, the electrocatalysis of polysulfide conversion in **NGC‐3** is better than in **NGC‐2** and **NGC‐1**. Due to the superior electrocatalytic activity of **NGC‐3**, the batteries comprising **NGC‐3/S** cathodes were cycled at different scan rates to understand Li‐ion diffusion kinetics. Figure S39 (Supporting Information) shows the CVs of **NGC‐3/S** cathodes at different scan rates, and the oxidation and reduction peak currents show a linear relationship with the (scan rate)^1/2^, which further supports the faster Li─ion diffusion kinetics in Li‐S batteries comprised of **NGC‐3**.

**Figure 5:**
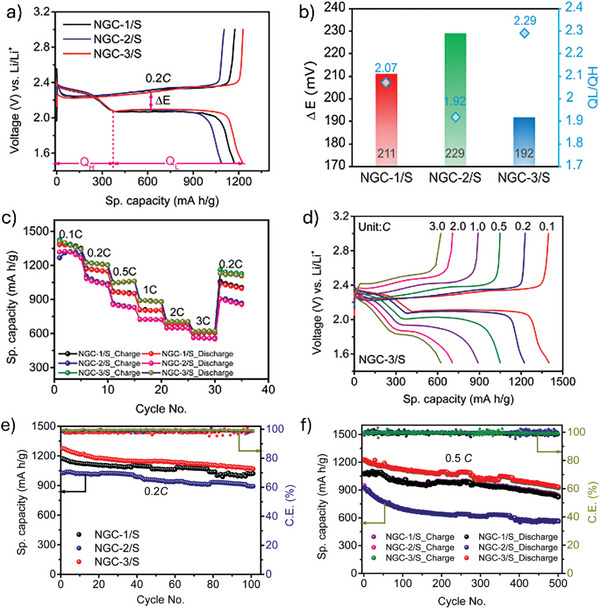
a) Charge–discharge profiles of **NGC**s at 0.2C. b) Plot showing ΔE and Q_L_/Q_H_ values for **NGC**s obtained from the GCD profile. c) Rate performance of **NGC**s. d) Charge–discharge profile of the cell comprising **NGC‐3/S** cathode at various C rates. e) Cyclic stability profile of **NGC/S** cathodes at 0.2C. f) Long‐term cycling of **NGC/S** cathodes at 0.5C.

To further verify the influence of structural features of **NGC**s on the Li─S redox process, galvanostatic charge/discharge (GCD) tests were conducted at various C rates. Initially, the cells comprising **NGC/** **S** cathodes were tested at 0.2C, where the GCD curve displayed two typical discharge plateaus at ≈2.3 (Li_2_S_8_ to Li_2_S_6_) and 2.07 V (Li_2_S_6_ to Li_2_S_2_/Li_2_S), respectively, consistent with the CV profiles (**Figure** **5**a).^[^ ^49^ ^]^ The initial discharge capacity of the cell comprising **NGC‐3/**S cathode at 0.2C is 1276 mAh g^−1^, which is higher than the cells comprising **NGC‐1/**S (1172 mAh g^−1^) and **NGC‐2/** **S** (1083 mAh g^−1^) cathodes. Interestingly, the **NGC‐3/** **S** cathode showed the smallest polarization potential (ΔE) of 192 mV compared to 211 and 229 mV for **NGC‐1** and **NGC‐2**, respectively. (Figure 5b). Furthermore, the electrocatalytic activity of **NGC**s toward lithium polysulfide conversion was evaluated using the Q_L_/Q_H_ ratio (Q_L_ and Q_H_ represent the discharge capacity of the two plateaus). **NGC‐3/** **S** cathode displayed the highest Q_L_/Q_H_ ratio of 2.29 compared to **NGC‐2/** **S** (2.07) and **NGC‐1/** **S** (1.92) cathodes (Figure 5b). These observations demonstrate that the conversion kinetics of polysulfides by **NGC**s follows the order **NGC‐3**>**NGC‐2**>**NGC‐1**.

Next, the rate capabilities of the cells comprising **NGC/** **S** cathodes at different C rates from 0.1 to 3.0C were examined (Figure 5c). **NGC‐3/** **S** displayed average discharge capacity values of 1398, 1223, 1061, 888, 705, and 622 mAh g^−1^ at 0.1, 0.2, 0.5, 1.0, 2.0, and 3.0C, respectively. When the C rate was switched back to 0.2C, the cell could recover an average discharge capacity of 1121 mAh g^−1^, indicating excellent electrochemical reversibility and rapid polysulfide conversion efficiency. The corresponding charge‐discharge profiles for the cell comprising the **NGC‐3/** **S** cathode are shown in Figure 5d. On the other hand, the cells comprising **NGC‐1/** **S** (605 mAh g^−1^ at 3C) and **NGC‐2/** **S** (526 mAh g^−1^ at 3C) cathodes also showed good rate performance but had lower capacity values than **NGC‐3/** **S** cathodes. The improved electrochemical behavior could be attributed to polysulfides' strong physical and chemical confinement via abundant micropores and the electrostatic attraction of polysulfides through polar functionalities in **NGC**s (**Table** **1**).

**Table 1:** The table compares the performance metrics of **NGC**s.

Performance metrics of NGCs	NGC‐1/S	NGC‐2/S	NGC‐3/S
Initial discharge capacity (0.2C), mAh g^−1^	1172	1083	127 6
Polarization potential (ΔE), mV	211	229	192
Q_L_/Q_H_ ratio	1.92	2.07	2.29
Discharge capacity at 3.0C, mAh g^−1^	605	526	622
Discharge Gibbs free energy, eV	0.88	—	0.66
Activation energy for the decomposition of Li_2_S, eV	1.93	—	1.39
Discharge capacity (0.2C), mAh g^−1^	1172	1083	1276
Discharge capacity after 100 cycles (0.2C), mAh g^−1^	1017	891	1075
Discharge capacity (0.5C), mAh g^−1^	1073	907	1228
Discharge capacity after 500 cycles (0.5C), mAh g^−1^	805	571	929
Discharge capacity (1.0C), mAh g^−1^	981	728	1032
Discharge capacity after 500 cycles (1.0C), mAh g^−1^	642	517	839

Figure 3d shows the DFT‐calculated relative Gibbs free energy profile of the discharge process in **NGC‐1** and **NGC‐3**. The overall reduction reaction involving the reversible formation of Li_2_S from S_8_ and Li and the corresponding optimized structures is shown in Figures S37 and S38 (Supporting Information). The shift from S_8_ to Li_2_S_8_ for both cases marks a spontaneous and exothermic conversion in the initial stage, while subsequent lithiation steps become endothermic. The rate‐limiting step in the total discharge process is Li_2_S_2_@ **NGC** → Li_2_S@ **NGC** conversion with the highest positive Gibbs free energy of 0.88 and 0.66 eV for **NGC‐1** and **NGC‐3**, respectively. The lower Gibbs free energy for **NGC**‐**3** indicates that the S reduction is thermodynamically more favorable and thus easier than for other COFs.

The decomposition of Li_2_S is the first and key step of the charging process, which involves the breakage of Li─S bond and Li^+^ escape, *Li*_2_ *S* → *LiS* + *Li* ^+^ + *e* ^−^. We calculated the energy barrier of this process for both **NGC‐1** and **NGC‐3**. The reaction path and corresponding energy profile are shown in Figure 3c. The **NGC‐1** exhibits a higher calculated energy barrier for the decomposition of Li_2_S (1.93 eV) compared to **NGC‐3** (1.39 eV), suggesting the conversion from Li_2_S to higher Li polysulfides is more rapid and feasible in the charging process for **NGC‐3**. Next, the long‐term cycling stabilities of Li─S batteries comprising **NGC**/**S** cathodes at 0.2, 0.5, and 1.0C were carried out. As shown in Figure 5a, the Li─S cell with **NGC‐3**/**S** cathode delivered a high discharge capacity of 1276 mAh g^−1^ at 0.2C. It retained an average discharge capacity of 1075 mA h g^−1^ after 100 cycles with an average C.E. of 99%. On the other hand, the cells comprising **NGC‐1/** **S** and **NGC‐2/** **S** delivered an initial discharge capacity of 1172 and 1083 mAh g^−1^, respectively. After 100 cycles, the cells retained the capacity of 1017 (**NGC‐1/** **S**) and 891 mAh g^−1^ (**NGC‐2/** **S**) (Figure 5a). These results indicate that all the **NGC**s displayed a stable and reversible electrochemical conversion of lithium polysulfides with excellent capacity retention at 0.2C (Figure S43, Supporting Information). Further, the batteries were tested at 0.5C, where **NGC‐3**/**S** cathodes delivered a high initial specific capacity of 1228 mAh g^−1^. After 500 cycles, the cell retained a reversible discharge capacity of 929 mAh g^−1^ with an average C.E. of 99%. Similarly, **NGC‐1/** **S** had an initial discharge capacity of 1073 mAh g^−1^ with a capacity retention of 75% at the 500^th^ cycle, and **NGC‐2/** **S** cathodes showed an initial capacity of 907 mAh g^−1^ with 63% retention at the end of 500^th^ cycle (Figure 5f).

It is interesting to note that even at a high C rate of 1C, **NGC‐3**/**S** displayed a very high discharge capacity of 1032 mAh g^−1^ with 81% retention (839 mAh g^−1^) at the end of the 500^th^ cycle (Figure S44, Supporting Information). Similarly, **NGC‐1** and **NGC‐2** delivered a reversible discharge capacity of 981 mAh g^−1^ (65.4% retention at the end of the 500^th^ cycle) and 728 mAh g^−1^ (71% retention at the end of the 500^th^ cycle), respectively. While all **NGC**s displayed very good electrochemical behavior because of the rationally designed chemical environment within the structure of COFs, the presence of additional pyridine units in **NGC‐3** contributed to much stronger polysulfide adsorption and conversion kinetics and, in turn, a significant improvement in the battery performance even at higher C rates compared to the other **NGC**s. It is known that pyridine N improves the conversion kinetics and imparts electrocatalytic activity toward lithium polysulfides.^[^ ^46^, ^47^, ^48^ ^]^ Considering the superior performance, **NGC‐3**/**S** was cycled even at 2.0C. The cell's capacity initially started increasing up to a few cycles and reached a maximum of 990 mAh g^−1^, and then a steady decrease in the capacity was observed. Even after 1000 charge‐discharge cycles, the cell maintained an average discharge capacity of 605 mAh g^−1^ (Figure S44, Supporting Information).

**Figure 6:**
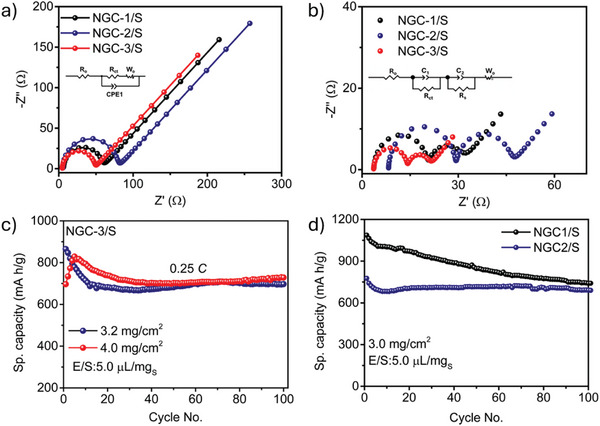
EIS of **NGC/S** cathodes a) before cycling and b) after 100 cycles (with the inset showing the equivalent circuit). c) Cyclic stability profile of **NGC‐3/S** cathodes at 0.25C with 3.2 and 4.0 mg cm^−2^ sulfur loading. d) Cyclic stability profile of **NGC‐1/S** and **NGC‐2/S** cathodes at 0.1C with sulfur loading of 3.0 mg cm^−2^.

To understand the kinetics of Li‐ions diffusion at the electrode/electrolyte interface, electrochemical impedance spectroscopic measurements of the cells were recorded at the initial stage and after 100 charge–discharge cycles. The Nyquist plots for all the batteries with **NGC** **/S** cathodes displayed a single semicircle in the high‐mid frequency region, which is assigned to charge‐transfer resistance (R_ct_). The **NGC‐3/** **S** cathode showed the smallest R_ct_ of 42.6 Ω compared to **NGC‐2/** **S** (50.5 Ω) and **NGC‐1/** **S** (72.6 Ω) (**Figure** **6**a). The low R_ct_ of **NGC‐3** **/S** is again attributed to the strong polysulfide adsorption and improved redox conversion kinetics compared to the other two COFs. Notably, the Nyquist plots of the batteries after cycling showed an additional semicircle corresponding to the resistance contributions from solid Li_2_S_2_/Li_2_S deposits at the electrode/electrolyte interface. All the cells had reduced R_ct_, which shows their effectiveness in polysulfide conversion kinetics with improved charge transfer properties. The R_ct_ values for the cell with **NGC‐3/** **S**, **NGC‐2/** **S**, and **NGC‐1/** **S** were found to be 10.7, 17.6, and 25.9 Ω, respectively (Figure 6b). The smaller R_ct_ of **NGC‐3/** **S** comprising the cell stems from the favorable electronic environment for the lithium polysulfide adsorption, which further facilitates their accelerated conversion kinetics during the charge–discharge process.

Furthermore, the batteries were tested at practically relevant conditions with high sulfur‐loaded cathodes under lean electrolyte conditions (electrolyte to sulfur ratio (E/S):5.0 µL mg^−1^). **NGC‐3/** **S** with sulfur loading 3.2 and 4.0 mg cm^−2^ with an E/S ratio of 5.0 µL mg^−1^ was tested (Figure 6c). The cell with 3.2 mg sulfur loading delivered an initial discharge capacity of 864 mAh g^−1^ at a 0.25C rate with a capacity retention of 81% (697 mAh g^−1^) at the 100th cycle. On the other hand, the cell with 4.0 mg sulfur loading showed a discharge capacity of 828 mAh g^−1^ at 0.25C rate with a capacity retention of 88% (728 mAh g^−1^) after 100 cycles. Similarly, the batteries with **NGC‐1/** **S** and **NGC‐2/** **S** cathodes with 3.0 mg cm^−2^ sulfur loading were tested under lean electrolyte conditions. The **NGC‐1/** **S** cathode showed a discharge capacity of 1087 mAh g^−1^ at 0.1C with a steady decrease in capacity to retain a capacity of 740 mAh g^−1^ at the 100th cycle. Meanwhile, **NGC‐2/** **S** cathodes delivered a 776 mAh g^−1^ capacity at 0.1C while maintaining a 691 mAh g^−1^ capacity at the end of the 100th cycle (Figure 6d). These results again demonstrate that the batteries’ improved performance, even at higher sulfur loading and under lean electrolyte conditions, is related to the rational design strategy of the **NGC**s with dual confinement for sulfur and polysulfides. It is interesting to note that even under such operating conditions, the materials remain stable, which is reflected in the batteries' stable electrochemical performance with negligible capacity fade.

The cells were disassembled after the first few cycles to understand the interaction between the **NGC**s and the polysulfides, and the electrodes were subjected to ex‐situ XPS analysis. **NGC‐3/S** electrodes showed three peaks in Li 1s spectra centered at 55.5, 55.9, and 56.4 eV, corresponding to Li─S, Li─N, and Li─Ointeractions, respectively (Figure S47b, Supporting Information). The N 1s spectrum of **NGC3/S** (Figure S47c, Supporting Information) shows the evolution of the N─Li peak situated at 398.8 eV, which stems from the participation of pyridine‐N in immobilizing the polysulfides through electrostatic interactions, which in turn improves their conversion kinetics in improving the overall electrochemical performance. The S 2p spectrum displayed two peaks at 161.4 and 163.2 eV, corresponding to the Li_2_S and Li_2_S_y_ (8>y>4). Also, the peaks centred at 166.5, 168.5, and 169 eV correspond to the thiosulphate, polythionate, and sulfate groups, respectively (Figure S47d, Supporting Information). Furthermore, C 1s (Figure S47a, Supporting Information) and O 1s (Figure S47b, Supporting Information) spectra showed the presence of ─C─O─Li_2_S_x_ peaks, indicating the lithium polysulfides and carbonyl oxygen interactions. Similar peaks were observed in the C 1s and O 1s XPS spectra of **NGC‐1/S** and **NGC‐2/S**: the Li 1s spectra of **NGC‐1/**S and **NGC‐2/S** showed two peaks at 54.8 and 55.4 eV corresponding to Li─O and Li─S, respectively (Figures S45–S47, Supporting Information).^[^ ^35^, ^36^, ^37^, ^38^, ^39^, ^51^ ^]^ The **NGC** COF frameworks exhibit effective stabilization of LiPSs through a combination of coordination interactions and anion‐*π* interactions enabled by chalcone, nanographene‐like polyaromatic cores, and pyridine functionalities. The  carbonyl groups in chalcone moieties stabilize LiPSs via electrostatic interactions with Li⁺, while the polyaromatic core facilitates anion*–π* interactions with LiPSs species, significantly reducing dissolution and migration, as evidenced by deconvoluted XPS spectra of post‐cycled **NGC‐1** and **NGC‐2** cells. Pyridine functionalities further enhance LiPSs immobilization by forming more stable coordination interaction with Li⁺ ions, as confirmed by XPS analysis of **NGC‐3** cells. In **NGC‐3**, the synergistic interplay of chalcone, polyaromatic cores, and pyridine functionalities effectively adsorbs and immobilizes LiPSs, mitigating migration while promoting uniform Li₂S nucleation and deposition during discharge, as supported by deposition‐dissolution experiments. These mechanisms, coupled with the robust structural integrity of the COF framework during cycling, ensure suppression of the shuttle effect and enhanced battery performance, as confirmed through post‐cycling XPS analysis, deposition‐dissolution studies, and DFT calculations. A schematic representation of the mechanistic insights is provided in Figure S48 (Supporting Information).^[^ ^34^, ^35^, ^36^, ^37^, ^38^, ^39^ ^]^ Overall, molecular‐level effective interactions offered by designed COFs resulted in highly efficient and stable battery performance (Table 1, S7, Supporting Information).

## 3 Conclusion

In summary, we have demonstrated three chalcone‐linked, sp^2^‐bonded nanographene‐type COFs as efficient sulfur‐hosting cathode materials for lithium‐sulfur batteries. Our design strategy to develop a sulfur host cathode that enables both physical (using micropores) and chemical (chalcone and pyridine functionalities) encapsulation of LiPSs offers a new avenue to overcome the challenges of COF‐based sulfur hosts. We have elucidated the critical role of pyridine and chalcone functionalities in mitigating the shuttle effect caused by intermediate LiPSs. The COFs display high capacities, outstanding rate performance, and superior long‐term stability, surpassing most reported polymeric sulfur host materials. We have also performed DFT calculations to investigate the mechanism of sulfur immobilization, further conversion into LiPSs, and the binding energies of polysulfides into COFs to provide reliable structure‐property correlations with experimental observations. These findings will pave the way for developing efficient and high‐performance future energy storage devices.

## Conflict of Interest

The authors declare no conflict of interest.

## Supporting information



Supporting Information

## Data Availability

The data that support the findings of this study are available in the supplementary material of this article.
