# Supplementary material for: Heteroatom‐Synergistic Effect on Anchoring Polysulfides In Chalcone‐Linked Nanographene Covalent Organic Frameworks for High‐Performance Li─S Batteries
Source: Adv Sci (Weinh). 2025 Feb 25;12(16):2415897. doi: 10.1002/advs.202415897 (PMC12021064; doi:10.1002/advs.202415897)
Supplement: Supplementary file 1 — Supporting Information [file ADVS-12-2415897-s001.docx]

Supporting Information for

**Heteroatom-Synergistic Effect on Anchoring Polysulfides in Chalcone-linked Nanographene Covalent Organic Frameworks for High-Performance Li‑S Batteries**

*Kayaramkodath C. Ranjeesh, ^[a] ≠^ Bharathkumar H. Javaregowda, ^[b]^ ^≠^ Safa Gaber, ^[a]^ ^≠^ Preeti Bhauriyal, ^[c]^ Sushil Kumar,^[a]^ Tina Skorjanc, ^[d]^ Matjaž Finšgar,* *^[e]^ Thomas Heine**, ^[c,f,g]^ Kothandam Krishnamoorthy,^[b]^ * Dinesh Shetty^[a,h]^ **

[a] Department of Chemistry, Khalifa University of Science & Technology, Abu Dhabi, United Arab Emirates. E-mail: dinesh.shetty@ku.ac.ae

[b] Polymer Science and Engineering, National Chemical Laboratory (CSIR-NCL), Pune-411008, India. Email: k.krishnamoorthy@ncl.res.in

[c] Faculty of Chemistry and Food Chemistry, Theoretical Chemistry Technische Universität Dresden, 01069 Dresden, Germany.

[d] Materials Research Laboratory University of Nova Gorica Vipavska cesta 11c, Ajdovscina 5270, Slovenia

[e] Faculty of Chemistry and Chemical Engineering, University of Maribor Smetanova ulica 17, Maribor 2000, Slovenia

[f] Helmholtz-Zentrum Dresden-Rossendorf, Center for Advanced Systems Understanding, CASUS, Untermarkt 20, 02826 Görlitz, Germany

[g] Department of Chemistry and ibs for Nanomedicine, Yonsei University, Seodaemun-gu, Seoul 120-749, South Korea

[h] Center for Catalysis & Separations (CeCaS) Khalifa University of Science & Technology Abu Dhabi P.O. Box 127788, UAE

**Content**

| 1. Materials | Page S2 |
| --- | --- |
| 2. Synthesis | Page S2-S5 |
| 3. Instruments and Methods | Page S5-S9 |
| 4. Supporting figures and tables | Page S10-S53 |
| 6. References | Page S53-S55 |

**1.** **Materials**

1,4-Diacetylbenzene (**DB**) (TCI), 4,4'-diacetylbiphenyl (**DBP**) (TCI), 2,6-diacetylpyridine (**DP**) (TCI), 2,3,6,7,10,11-hexakis(4-formylphenyl)triphenylene (**HFPTP**) (ET Co., Ltd), potassium hydroxide (KOH) (Sigma-Aldrich), sodium hydroxide (NaOH), 1,4-dioxane (Thermo Scientific, Extra dry over molecular sieve), and ethanol (EtOH) (Merck), tetrahydrofuran (THF) (Merck), acetone (Merck), were used as received. All the reactions were carried out in oven-dried 35-ml heavy-walled (HW) pressure glass vessel capped with a Teflon screw cap with rubber internal thread under an argon (Ar) atmosphere unless otherwise mentioned. All the aqueous solutions were prepared, and water washing was done using deionized water obtained from the Millipore system (15 MΩ).

N-methyl-2-pyrrolidone (NMP), sulfur, lithium sulfide (Li_2_S), lithium bis(trifluoromethane) sulfonimide (LiTFSI), lithium nitrate (LiNO_3_), 1,2-dimethoxyethane anhydrous (99.5%, inhibitor-free, DME), and 1,3-dioxolane (99.5%, DOL) were purchased from Sigma-Aldrich chemicals. Poly (vinylidene fluoride) PVDF, Super P carbon, carbon-coated aluminum current collector, Li foil (300 μm), CR2032 coin cell components, and the CR2300 Celgard separator were procured from Global Nanotechnology Pvt. Ltd. All reagents were used as received without any further purification.

**2. Synthesis**

**2.1 Model compound (E)-chalcone synthesis:** (E)-chalcone was synthesized by a base-catalyzed Claisen-Schmidt reaction in which benzaldehyde (24.29 mg, 0.228 mmol) and acetophenone (25 mg, 0.208 mmol) were added sequentially in 10 mL of ethanol. The reaction was conducted in a sealed pressure tube with a catalytic amount of potassium hydroxide (300 µl, 4M). After heating at 70 °C for 24 hours, at the end of the reaction, 50 ml of water was added, and the reaction mixture was refrigerated until the product precipitated (~5 hours). The crude product was obtained by filtration, washed with water to neutralize the pH, collected, and dried. The fine product, which was obtained as a white solid (low melting) by recrystallization from acetone, had an isolated yield of 90% (39 mg).

**Scheme S1**. Synthetic scheme of model compound **(E)-chalcone**.

^1^H NMR (500 MHz, CDCl_3_) δ 8.05 (d, J = 8.9 Hz, 2H), 7.74 (d, J = 15.6 Hz, 1H), 7.54 (d, J = 8.8 Hz, 2H), 7.51 (d, J = 15.6 Hz, 1H), 7.67-7.35 (m, 6H).^13^C NMR (125 MHz, CDCl_3_) δ 190.62, 144.85, 134.79, 132.81, 130.60, 128.65, 121.78. LCMS (ESI^+^ ): calcd for [M+H]^+^ 209.10, found 209.12.

**2.2 General Synthesis of NGC** COFs: 2,3,6,7,10,11-hexakis(4-formylphenyl)triphenylene (1 equiv) and corresponding diacetyl compound (1,4-diacetylbenzene, 4,4'-diacetylbiphenyl, diacetylpyridine) (3 equiv) were added sequentially in a 35-mL pressure tube, closed with a rubber septum, and purged with Argon. 2 ml of 1,4-dioxane/EtOH mixture (1.2: 0.8 v/v, before being purged and saturated with Ar) was introduced. After purging the reaction mixture for an hour, a catalytic amount of potassium hydroxide (300 µl, 4M aqueous, before being purged and saturated with Ar) was added. The reaction mixture was sonicated for 10 minutes. The pressure tube was capped in an Ar-saturated atmosphere and heated at 95 °C for five days. The yellow precipitate was collected by centrifuging and washed several times with water until the pH reached neutral. Further purification of the COF was carried out by Soxhlet extraction with THF for 48 hours to remove any impurities and unreacted products. The sample was vacuum-dried for 12 hours at 80 °C.

**2.2.1** **Synthesis of NGC-1**

Employing the general procedure and using 2,3,6,7,10,11-hexakis(4-formylphenyl)triphenylene (**HFPTP**) (25 mg, 0.029 mmol) and 1,4-diacetylbenzene (**DB**) (14.26 mg, 0.087 mmol), **NGC-1** COF was obtained as a yellow solid in 85 % (33.5 mg) isolated yield.

**Scheme S2**. Synthetic scheme for the preparation of **NGC-1**.

**2.2.2** **Synthesis of NGC-2:** Employing the general procedure and using 2,3,6,7,10,11-hexakis(4-formylphenyl)triphenylene (**HFPTP**) (25 mg, 0.029 mmol) and 4,4'-diacetylbiphenyl (**DBP**) (20.95 mg, 0.087 mmol), **NGC-2** COF was obtained as a yellow solid in 82 % (38 mg) of the isolated yield.

**Scheme S3**. Synthetic scheme for the preparation of **NGC-2**.

**2.2.3 Synthesis of NGC-3:** To execute the reaction, 2,3,6,7,10,11-hexakis(4-formylphenyl)triphenylene **(HFPTP)** (25 mg, 0.029 mmol) and diacetylpyridine (**DP**) (14.35 mg, 0.087 mmol) were used as per the general synthesis procedure to obtain **NGC-3** COF, which was obtained as a yellow solid in 78 % (31 mg) of the isolated yield.

**Scheme S4**. Synthetic scheme for the preparation of **NGC-3**.

**Table S1**. Optimization of synthesis conditions for **NGC-1**.

| **Entry** | **Solvents** | **Base** | **T (°C)** | **Crystallinity^#^** |
| --- | --- | --- | --- | --- |
| **NGC-1a** | 1,4-dioxane | 4M aqueous KOH | **95** | **Low** |
| **NGC-1b** | 1,4-dioxane | 6M aqueous KOH | **95** | **Moderate** |
| **NGC-1c** | 1,4-dioxane: EtOH (1.2: 0.8 v/v) | 1M aqueous NaOH | **95** | **High** |
| **NGC-1c** | 1,4-dioxane: EtOH (1.2: 0.8 v/v) | 4M aqueous KOH | **95** | **Highest** |

**^#^** Defined based on the intensity of the peak at (2θ = ~5 °)

**3. Instruments and methods**

**Powder X-ray diffraction (PXRD)**: Powder X-ray diffraction measurements were performed on Rigaku Smart Lab II with Cu Kα (λ = 1.5405 Å) radiation source operating at 40 kV and 40 mA. The patterns were recorded with a divergent slit of 1/16° over the 2Ɵ range of 2–50° with step size = 0.02°.

**Fourier transform infrared spectroscopy (FT-IR)**: FT-IR spectra were taken on a Bruker Optics ALPHA-E spectrometer with a universal Zn-Se ATR (attenuated total reflection) accessory in the 600- 4000 cm^-1^ region or using a Diamond ATR (Golden Gate) with 24 scan rate and 4 cm^-1^ resolution.

**Solid-state** **^13^Carbon Cross-Polarization Magic Angle Spinning (CP MAS)**: ^13^C CP MAS NMR spectra of the COFs were recorded on a Bruker Avance NEO 500MHz NMR spectrometer using a 4.0mm MAS probe at ambient temperature and a magic angle spinning rate of 12.0 kHz. Spectra were acquired using a CP contact time of 2000us, a recycle delay of 2sec and a total number of 42200 scans. ^13^C chemical shifts were externally referenced to the adamantane CH_2_ signal at 38.46 ppm. NMR data were processed using the software “TopSpin 4.1.4”.

**BET analysis:** Porosity analyses were performed using Anton Paar Autosorb iQ combined physisorption and chemisorption instrument. For each measurement, 20 – 30 mg of COF samples were used. The samples were activated at 80 °C for 16 hours before being subject to N_2_ gas adsorption in a liquid N_2_ bath (77 K) to collect full isotherms. Surface areas were calculated using the Multipoint Brunauer – Emmett – Teller (BET) model, and pore size distributions were evaluated/calculated using the non-local density functional theory (NLDFT).

**Morphology and elemental analysis**

The morphology of the materials was characterized by scanning electron microscopy (SEM, JEOL JSM-7610F FEG-SEM. The SEM samples were prepared by drop-casting 10 μL of COF slurry (COFs dispersed in isopropyl alcohol) on a silicon substrate and dried in air, followed by Pt coating (nano-sized film) using the JEOL JEC-300FC Auto Fine before SEM analysis. Further, transmission electron microscopy (TEM) and high-resolution TEM (HR-TEM) were employed for an in-depth morphological analysis by FEI Tecnai TEM 20 kV. The TEM samples were prepared by drop casting the COFs dispersion (dispersed in isopropyl alcohol) over carbon grids (TED PELLA, INC. 200 mesh) and allowed to dry overnight in a desiccator.

**Thermogravimetric analysis (TGA)**: TGA analysis was performed using a PerkinElmer Simultaneous Thermal analyzer STA 6000 under N_2_ environment at a heating rate of 15 ^o^C min^-1^ and a temperature range of 30-900 °C.

**X-ray photoelectron spectroscopy (XPS):** XPS measurements were performed using a Supra+ instrument (Kratos, Manchester, UK) equipped with an Al K_α_ excitation source and a monochromator. The charge neutralizer was on during the measurements. The take-off angle was 90°. XPS measurements and data processing were performed using ESCApe 1.5 software (Kratos). The powder samples were placed on a carbon tape attached to the silicon wafer. The area analyzed was 300 by 700 microns. The measurements were performed at a pass energy of 20 eV. The base pressure in the main analysis chamber was 8·10^–8^ mbar. The binding energy scale was corrected based on the C-C/C-H peak at 284.8 eV in the C 1s spectrum.

**Preparation of NGCs/S composite**

The NGCs/S composite was prepared by melt-diffusion strategy. For this, **NGC**s and sulfur with a mass ratio of 1:4 were grounded for 30 minutes, followed by heating at 155 °C for 12 h under Ar atmosphere. Finally, the resulting mixture was cooled to room temperature.

**Polysulfide adsorption test**

0.625 M Li_2_S_6_ solution was prepared by mixing S and Li_2_S in a molar ratio 5:1 in a 1,2-dimethoxyethane (DOL): 1,2-dimethoxyethane anhydrous (DME), (1:1) solvent mixture inside an Ar-filled glovebox. The mixture was stirred for 24 hours at 80 °C, which resulted in a homogeneous dark brown solution. This solution was diluted to 0.0005 M, and the **NGC-1**, **NGC-2**, and **NGC-3** were added into the solution and left overnight undisturbed for 12 hours. Further, the supernatant solution was analyzed using UV-visible absorption spectroscopy.

**Lithium polysulfide electrocatalysis test**

For the polysulfide electrocatalysis, standard 2025-coin cells comprising two identical electrodes of **NGC-1/NGC-2/NGC-3** were assembled using 40 μL Li_2_S_6_ electrolyte. The cells were cycled between -1.0 V and +1.0 V at a scan rate of 10.0 mV s^-1^.

**Electrochemical measurements**

The cathodes were prepared by casting a homogeneous slurry of **NGCs/S** composite, carbon black, and PVDF binder in an 8:1:1 ratio in the NMP solvent onto a carbon-coated aluminum current collector. The casted film was dried in a vacuum oven for 8h at 120 °C and hot roll pressed. The sulfur loading in the electrodes was maintained at 1.0-2.0 mg/cm^2^, and the electrolyte-to-sulfur ratio was maintained at 20 μL/mg_S_. The cells were assembled inside an argon-filled glovebox (O_2_ ≤0.1 ppm and H_2_O ≤0.1 ppm) using NGCs/S cathode, Li foil as counter and reference electrode, and 1.0 M LiTFSI electrolyte with 2 wt. % LiNO_3_ additive in the DOL: DME (1:1) mixture. Galvanostatic charge-discharge experiments were done using NEWARE battery testers with a potential window of 1.5 to 3.0 V against Li/Li^+^. Cyclic voltammetry tests were carried out using BioLogic VSP-300 potentiostat within the potential window of 1.5 V to 3.0 V. The Electrochemical Impedance Spectroscopic (EIS) analysis was carried out in the frequency range of 10 mHZ to 1 MHz with an amplitude of 10 mV.

**Computational Details**

To identify the stacking of COFs, structural simulations are carried out using self-consistent charge density functional-based tight-binding (SCC-DFTB) in Amsterdam Modelling Suite (AMS) ADF 2023 with 3ob-3-1 parameters and D3(BJ) dispersion correction. ^(reference 40,41 in the main manuscript)^ Three distinct stackings of the 2D layers are considered: AA-slipped, AB, and ABC.

Calculations in this work are performed using the Vienna ab initio Simulation Package (VASP) code based on periodic density functional theory (DFT).^1^ Therefore, we have used the Perdew–Burke–Ernzerhof (PBE) form of the Generalized Gradient Approach (GGA) to perform the exchange-correlation function.^2^ The ions are modeled with the projector augmented-wave (PAW) method.^3,4^ Grimme's DFT-D3 approach is utilized to accurately account for long-range van der Waals (vdW) forces.^5^ Self-consistent field calculations are performed with a convergence criterion of 1 × 10^−5^ eV per atom. The cutoff energy is set to 520 eV, and the magnitude of the force on atoms is minimized to be less than 10^−3^ eV Å^−1^. The binding energy and charge/discharge calculations are carried out with corresponding monolayers of **NGC-1**, **NGC-2,** and **NGC-3** and with 1 × 1 × 1 Gamma-pack k-point grid. A vacuum space of 20 Å along the z-direction is adopted to avoid interactions between two layers in the nearest neighbouring unit cells. The nudged elastic band (NEB) method implemented in VASP was performed to investigate the transition state searches.^6^

The Gibbs free energy change (ΔG) is defined as,

ΔG = ΔE + ΔZPE − TΔS

Here, ΔE is the total energy difference obtained from DFT calculations, and ΔZPE is the change of zero-point energy (ZPE). The entropy contribution TΔS is approximated to be negligible at 0 K. The ZPE of the system is calculated by considering only the degrees of freedom of adsorbed species. We have used the Nørskov model^7^ where the energy of an added/removed (Li^+^ + e^−^) is given by bulk Li, since it is assumed that G(Li) = G(Li^+^ + e^−^), i.e., equilibrium at the anode.

We considered, three distinct stackings, namely, AA-slipped stacking, AB, and ABC (as shown in Figure S1-3, supporting information). In case of all three COFs, we observed a distinct contrast in ABC stackings in comparison to AA and AB stacking when analyzing their simulated X-ray diffraction (XRD) patterns. Furthermore, the simulated XRD patterns of ABC stackings of these COFs match best with the experimental PXRD patterns. This suggests that **NGC-1**, **NGC-2,** and **NGC-3** furnish ABC stacking. The initial distinctive low-angle 2θ peak is detected at 5.65°, 5.58°, and 5.53° for **NGC-1**, **NGC-2** and **NGC-3**, respectively. These angles correspond to the (111) reflection plane.

Pawley refinement gives optimized lattice parameters; a = 22.88 Å, b = 28.73 Å, c = 20.00, α = 90.00**^◦^**, ꞵ = 90.00**^◦^**, γ = 120.87**^◦^** for **NGC-1**, a = 33.40 Å, b = 33.40 Å, c = 18.90, α = 90.00**^◦^**, ꞵ = 90.00**^◦^**, γ = 120.00**^◦^** for **NGC-2**, and a = 28.50 Å, b = 28.50 Å, c = 18.80, α = 90.00**^◦^**, ꞵ = 90.00**^◦^**, γ = 120.00**^◦^** for **NGC-3**.


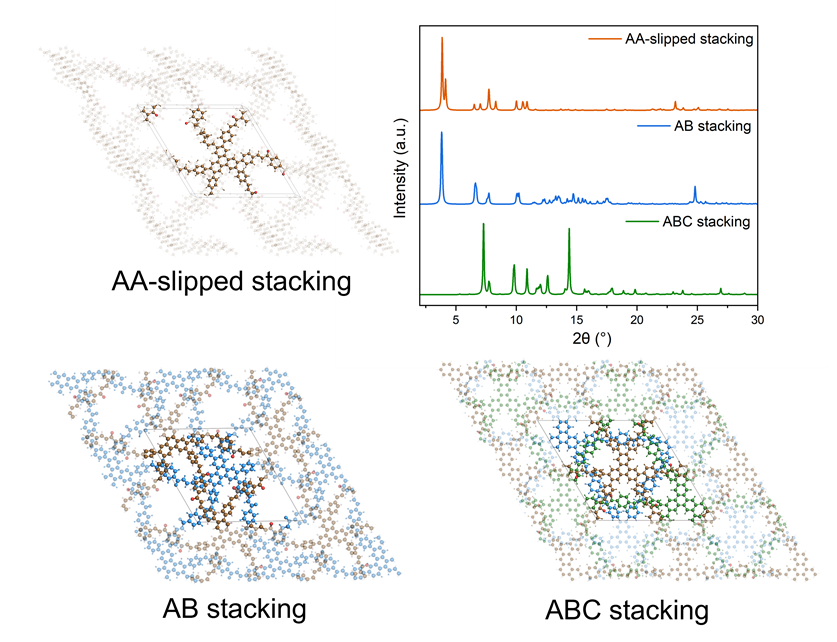


**Figure S1**: Simulated PXRD patterns of three different layer stackings of **NGC-1**.


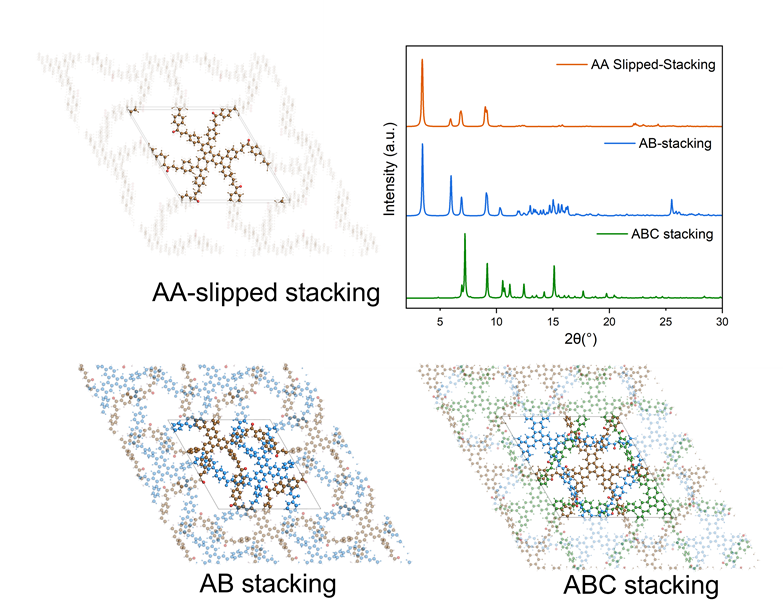


**Figure S2**: Simulated PXRD patterns of three different layer stackings of **NGC-2**.

**
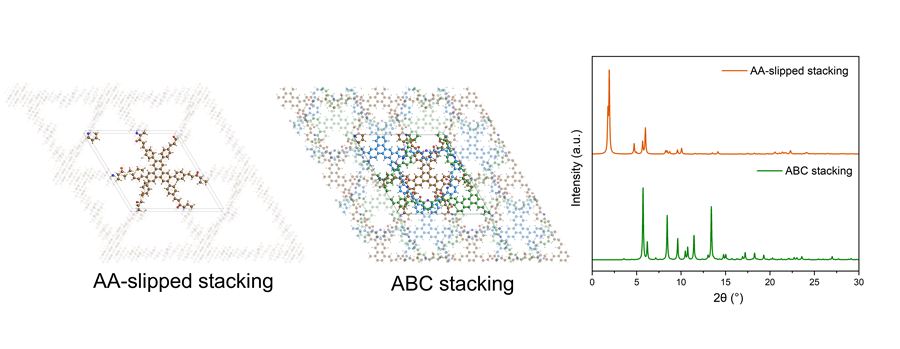
**

**Figure S3**: Simulated PXRD patterns of three different layer stackings of **NGC-3**.

**Structural modeling and Pawley refinement**

The sharp nature of the peaks in the PXRD patterns suggest that the **NGC-1,2,3** are crystalline materials. To correlate the experimental PXRD pattern with the simulated one, the two-dimensional modeling was performed in VASP. Refinements of the PXRD pattern were done using Pawley refinement of the Material Studio software.


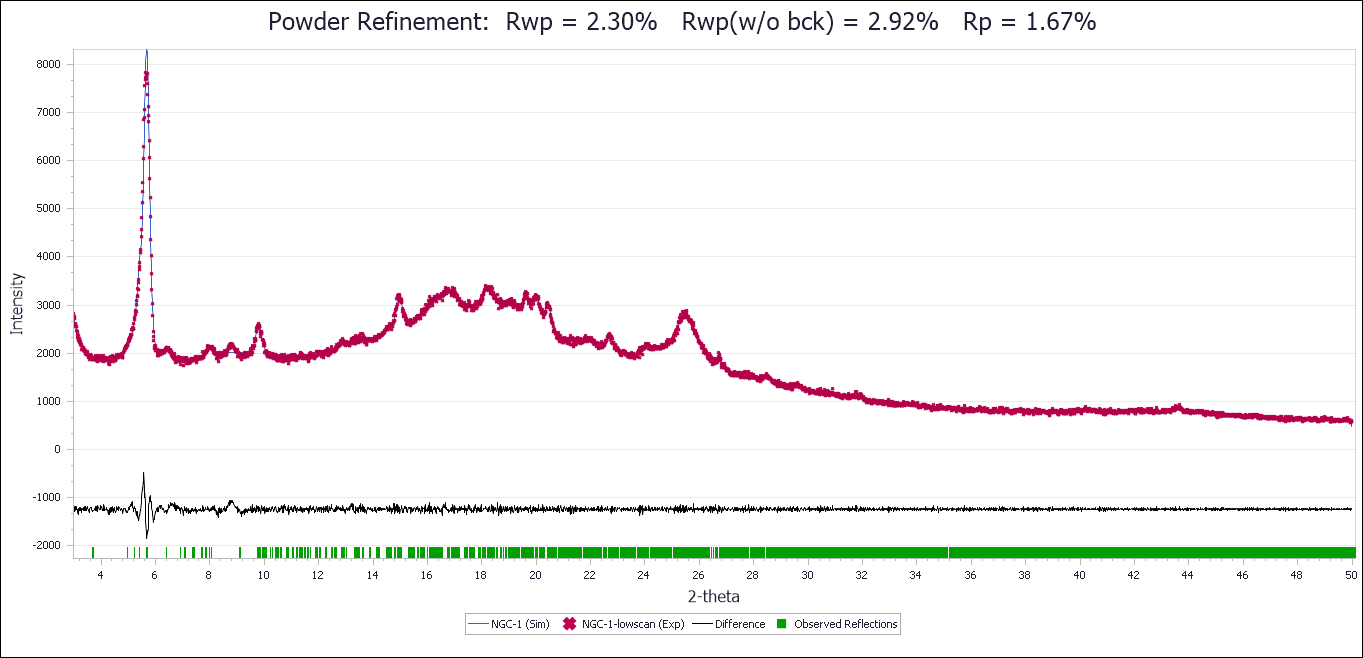


Figure S4: Pawley refinement of NGC-1. The experimental PXRD pattern after Pawley refinement is in good agreement with the simulated one.

**Fractional atomic coordinates of a unit cell of NGC-1.**

Table S2. Fractional atomic coordinates of a unit cell and lattice parameters of NGC-1.

| **NGC-1 ABC model; Space group P1**  **a = 27.74 Å, b = 27.59 Å, c = 17.53 Å; α = 138.59°, β = 73.72°, γ = 119.98°** | | | | | | | | | | | |
| --- | --- | --- | --- | --- | --- | --- | --- | --- | --- | --- | --- |
| **Atoms** | x | y | z | **Atoms** |  | y | z | **Atoms** | x | y | z |
| **H1** | 0.45584 | 0.35157 | -0.08313 | **H54** | 0.2846 | 0.81994 | 0.50466 | **H110** | -0.09812 | 0.1289 | 0.70025 |
| **H2** | 0.704 | 0.0387 | -0.02628 | **H55** | 0.79503 | 0.0703 | 0.27055 | **H111** | 0.63819 | 0.29932 | 0.5603 |
| **H3** | -0.04976 | 0.12094 | 0.17958 | **H56** | 0.55781 | 0.40117 | 0.35807 | **H112** | 0.31571 | 0.58488 | 0.62393 |
| **H4** | -0.04989 | -0.1087 | -0.06478 | **H57** | 0.29203 | 0.58369 | 0.23439 | **H113** | 0.88096 | 0.93945 | 0.6839 |
| **H5** | 0.2221 | 0.6772 | 0.00071 | **H58** | 0.96428 | 0.8583 | 0.30715 | **H114** | 0.39194 | 0.22337 | 0.70375 |
| **H6** | -0.02106 | 0.1171 | -0.07982 | **H59** | 0.53661 | 0.21336 | 0.3421 | **H115** | 0.00441 | 0.33326 | 0.583 |
| **H7** | 0.62524 | 0.13044 | -0.05922 | **H60** | 0.04453 | 0.49481 | 0.36047 | **H116** | 0.01419 | 0.28719 | 0.64391 |
| **H8** | 0.19828 | 0.49255 | -0.00254 | **H61** | 0.659 | 0.6272 | 0.29116 | **H117** | 0.76171 | 0.58984 | 0.60891 |
| **H9** | 0.70609 | 0.7827 | 0.02624 | **H62** | 0.67436 | 0.57946 | 0.33733 | **H118** | 0.27809 | 0.78332 | 0.75685 |
| **H10** | 0.07392 | 0.13445 | -0.07526 | **H63** | 0.41236 | 0.85881 | 0.27564 | **H119** | 0.51239 | 0.70731 | 0.66809 |
| **H11** | 0.33699 | -0.15183 | -0.03026 | **H64** | 0.93237 | 1.05299 | 0.41539 | **H120** | 0.86595 | 0.86874 | 0.72038 |
| **H12** | 0.02179 | -0.00703 | -0.05668 | **H65** | 0.16064 | 0.98842 | 0.37182 | **H121** | 0.09155 | 0.76509 | 0.69323 |
| **H13** | 0.59145 | 0.32867 | 0.07118 | **H66** | 0.52107 | 0.14705 | 0.38758 | **H122** | 0.3499 | 0.74278 | 0.70176 |
| **H14** | 0.82226 | 0.25016 | -0.01751 | **H67** | 0.74704 | 0.0501 | 0.37961 | **H123** | 0.66095 | 0.27504 | 0.73027 |
| **H15** | 0.18106 | 0.42149 | 0.03457 | **H68** | 1.00116 | 1.00663 | 0.35827 | **H124** | 0.6253 | 0.51644 | 0.79496 |
| **H16** | 0.4091 | 0.32714 | 0.02972 | **H69** | 0.31266 | 0.54992 | 0.39315 | **H125** | 0.87775 | 0.49372 | 0.6903 |
| **H17** | 0.66075 | 0.28354 | 0.00792 | **H70** | 0.38197 | 0.84681 | 0.51737 | **H126** | 0.21091 | 0.07487 | 0.72819 |
| **H18** | 0.97642 | 0.83712 | 0.06142 | **H71** | 0.53101 | 0.78002 | 0.37853 | **H127** | 0.95454 | 0.51422 | 0.69989 |
| **H19** | 0.04777 | 0.1484 | 0.19421 | **H72** | 0.86657 | 0.36126 | 0.41822 | **H128** | 0.10482 | 0.69268 | 0.66923 |
| **H20** | 0.19278 | 0.06677 | 0.04474 | **H73** | 0.60863 | 0.80644 | 0.40048 | **H129** | 0.711 | 0.39496 | 0.555 |
| **H21** | 0.52684 | 0.65044 | 0.09114 | **H74** | 0.75938 | 0.97667 | 0.35678 | **H130** | 0.55428 | 0.51208 | 0.74101 |
| **H22** | 0.26863 | 0.07733 | 0.03885 | **H75** | 0.36546 | 0.68533 | 0.24087 | **H131** | 0.8225 | 0.50295 | 0.82436 |
| **H23** | 0.41832 | 0.24501 | -0.0106 | **H76** | 0.20485 | 0.77287 | 0.397 | **H132** | 0.55763 | 0.65872 | 0.6883 |
| **H24** | 0.43822 | 0.06153 | -0.09344 | **H77** | 0.79358 | 0.86118 | 0.57989 | **H133** | 0.0461 | 0.92224 | 0.75428 |
| **H25** | -0.13027 | 0.06873 | 0.06866 | **H78** | 0.20498 | 0.93626 | 0.3861 | **H134** | 0.75066 | 0.40212 | 0.82313 |
| **H26** | 0.45387 | 0.12888 | 0.20789 | **H79** | 0.70409 | 0.21265 | 0.45509 | **H135** | 0.12508 | 0.44601 | 0.80389 |
| **H27** | 0.86717 | 0.19717 | -0.00604 | **H80** | 0.40092 | 0.67925 | 0.49525 | **H136** | 0.04299 | 0.08517 | 0.64339 |
| **H28** | 0.36792 | 0.49783 | 0.12824 | **H81** | 0.78259 | 0.7399 | 0.50707 | **H137** | 0.58492 | 0.58471 | 0.65216 |
| **H29** | 0.06831 | -0.04267 | 0.14821 | **H82** | 0.70103 | 0.36143 | 0.30201 | **H138** | -0.01327 | 0.40421 | 0.56629 |
| **H30** | 0.44504 | 0.0097 | 0.13677 | **H83** | 0.23435 | 0.85403 | 0.32201 | **H139** | 0.4702 | 0.06817 | 0.63572 |
| **H31** | 0.36639 | 0.6452 | -0.0339 | **H84** | 0.63879 | 0.6966 | 0.27508 | **H140** | 0.1297 | 0.51538 | 0.59814 |
| **H32** | 0.89582 | 0.12519 | -0.04264 | **H85** | 0.12354 | 0.34653 | 0.30875 | **H141** | 0.57168 | 0.26777 | 0.64651 |
| **H33** | 0.53578 | 0.08442 | -0.10604 | **H86** | 0.78158 | 0.80484 | 0.30893 | **H142** | 0.84135 | 0.64908 | 0.68666 |
| **H34** | 0.79099 | 0.6637 | 0.03126 | **H87** | 0.22342 | 0.5404 | 0.30611 | **H143** | 0.10531 | 0.20317 | 0.67402 |
| **H35** | 0.88652 | 0.83217 | -0.01679 | **H88** | 0.49745 | 0.93847 | 0.37695 | **H144** | 0.1162 | 0.63648 | 0.86459 |
| **H36** | 0.16024 | 0.22103 | 0.03332 | **H89** | 0.76294 | 0.48533 | 0.34716 | **H145** | 0.34976 | 0.55483 | 0.65958 |
| **H37** | 0.42782 | 0.76396 | -0.00122 | **H90** | 0.77363 | 0.92909 | 0.56281 | **H146** | 0.99337 | 0.86478 | 0.50954 |
| **H38** | 0.4331 | 0.19495 | 0.18826 | **H91** | -0.00328 | 0.79656 | 0.28009 | **H147** | 1.01874 | 0.79177 | 0.50917 |
| **H39** | 0.65908 | 0.09063 | -0.03959 | **H92** | 0.65229 | 0.14375 | 0.18331 | **H148** | 0.89429 | 0.68932 | 0.62802 |
| **H40** | 0.3212 | -0.10538 | -0.08122 | **H93** | 0.67761 | 0.07517 | 0.1926 | **H149** | 0.22746 | 0.54235 | 0.59059 |
| **H41** | 0.30004 | -0.03805 | -0.10036 | **H94** | 0.54987 | 0.97322 | 0.31078 | **H150** | 0.89356 | 0.79942 | 0.72519 |
| **H42** | 0.21283 | 0.25619 | -0.03159 | **H95** | 0.87699 | 0.82262 | 0.28727 | **H151** | 0.49456 | 0.26413 | 0.70091 |
| **H43** | 0.21028 | 0.36045 | 0.05414 | **H96** | 0.54837 | 0.08221 | 0.40203 | **H152** | 0.1798 | 0.75945 | 0.77566 |
| **H44** | 0.80654 | 0.81416 | 0.00611 | **H97** | 0.14572 | 0.52996 | 0.34648 | **H153** | 0.36854 | 0.02762 | 0.6386 |
| **H45** | 0.49403 | 0.30857 | 0.09405 | **H98** | 0.83649 | 1.03828 | 0.44589 | **H154** | 0.13428 | 0.56611 | 0.88168 |
| **H46** | 0.69153 | 0.63474 | 0.05563 | **H99** | 0.02318 | 0.31205 | 0.32369 | **H155** | 0.28896 | -0.0016 | 0.64237 |
| **H47** | 0.14 | 0.06349 | 0.16202 | **H100** | 0.47286 | 0.78385 | 0.50642 | **H156** | -0.03623 | 0.24462 | 0.725 |
| **H48** | 0.6088 | 0.58043 | 0.00986 | **H101** | 0.94206 | 0.27827 | 0.3169 | **H157** | 0.1237 | 1.0255 | 0.7681 |
| **H49** | 0.28306 | -0.20741 | 0.02532 | **H102** | 0.61947 | 0.52365 | 0.39777 | **H158** | 0.22389 | 0.90196 | 0.62935 |
| **H50** | 0.44509 | 0.60471 | 0.14874 | **H103** | 0.78137 | 0.3145 | 0.46594 | **H159** | 0.98444 | 0.973 | 0.57953 |
| **H51** | 0.30963 | 0.54063 | -0.07981 | **H104** | 0.87583 | 0.1788 | 0.29713 | **C181** | 0.24543 | 0.72515 | 0.725 |
| **H160** | 0.3172 | 0.43113 | -0.15335 | **H105** | 0.64544 | 0.25422 | 0.25374 | **C182** | 0.94895 | 0.67256 | 0.66475 |
| **H161** | 0.34326 | 0.36365 | -0.14327 | **C91** | 0.89911 | 0.99853 | 0.39271 | **C183** | 0.21775 | 0.5911 | 0.63338 |
| **H162** | 0.53494 | 0.46105 | -0.05711 | **C92** | 0.60425 | 0.95834 | 0.35154 | **C184** | 0.39551 | 0.07947 | 0.65205 |
| **C1** | 0.52703 | 0.13439 | -0.0618 | **C93** | 0.86894 | 0.87119 | 0.32419 | **C185** | 0.08645 | 0.62005 | 0.69256 |
| **C2** | 0.47161 | 0.12159 | -0.05378 | **C94** | 0.04916 | 0.35914 | 0.32713 | **C186** | 0.97347 | 0.59434 | 0.68796 |
| **C3** | 0.26942 | 0.53892 | -0.04871 | **C95** | 0.74094 | 0.90796 | 0.38745 | **C187** | 0.73266 | 0.57526 | 0.65249 |
| **C4** | 0.55783 | 0.26959 | 0.03487 | **C96** | 0.62794 | 0.88238 | 0.38067 | **C188** | 0.90415 | 0.62124 | 0.67612 |
| **C5** | 0.26643 | 0.23926 | 0.00651 | **C97** | 0.38518 | 0.85268 | 0.329 | **C189** | 0.9797 | 0.76088 | 0.64063 |
| **C6** | 0.71602 | 0.67337 | 0.04333 | **C98** | 0.55931 | 0.9088 | 0.36556 | **C190** | 0.07844 | 0.30334 | 0.72186 |
| **C7** | 0.39931 | 0.17222 | 0.01281 | **C99** | 0.63542 | 0.04508 | 0.32462 | **C191** | 0.12824 | 0.97463 | 0.73206 |
| **C8** | 0.28848 | 0.15775 | 0.02671 | **C100** | 0.73544 | 0.59327 | 0.41678 | **C192** | 0.78219 | 0.45807 | 0.76608 |
| **C9** | 0.0477 | 0.13288 | -0.01744 | **C101** | 0.78514 | 0.26193 | 0.42515 | **C193** | 0.5893 | 0.25251 | 0.67086 |
| **C10** | 0.22157 | 0.19106 | 0.02273 | **C102** | 0.43324 | 0.74029 | 0.44654 | **C194** | 0.02531 | 0.12025 | 0.66245 |
| **C11** | 0.29859 | 0.32683 | -0.0189 | **C103** | 0.24136 | 0.52515 | 0.32986 | **C195** | 0.10181 | 0.53043 | 0.81267 |
| **C12** | 0.39965 | -0.13508 | 0.0506 | **C104** | 0.68247 | 0.39618 | 0.32253 | **C196** | 0.07112 | 0.4444 | 0.73369 |
| **C13** | 0.44815 | 0.54945 | 0.09847 | **C105** | 0.75891 | 0.82417 | 0.51359 | **C197** | 0.01869 | 0.43996 | 0.63663 |
| **C14** | 0.09871 | 0.02489 | 0.11 | **C106** | 0.72816 | 0.73838 | 0.43685 | **C198** | 0.65535 | 0.5329 | 0.75427 |
| **C15** | 0.90474 | 0.81585 | 0.00457 | **C107** | 0.67229 | 0.73279 | 0.34313 | **C199** | -0.03226 | 0.09947 | 0.67076 |
| **C16** | 0.34757 | 0.67683 | -0.02139 | **C108** | 0.31278 | 0.8296 | 0.45529 | **C200** | 0.04766 | 0.7457 | 0.67441 |
| **C17** | 0.41961 | 0.09098 | 0.1402 | **C109** | 0.62459 | 0.37307 | 0.32793 | **C201** | 0.84915 | 0.6123 | 0.68256 |
| **C18** | 0.38959 | 0.00539 | 0.06419 | **C110** | 0.70314 | 0.03054 | 0.3607 | **C202** | 0.71923 | 0.39865 | 0.61695 |
| **C19** | 0.33349 | -0.00272 | -0.03294 | **C111** | 0.50472 | 0.90154 | 0.37328 | **C203** | 0.07208 | 0.65729 | 0.68295 |
| **C20** | -0.02229 | 0.12553 | 0.12498 | **C112** | 0.37277 | 0.6863 | 0.29998 | **C204** | 0.50247 | 0.5486 | 0.70325 |
| **C21** | 0.28964 | 0.65384 | -0.01702 | **C113** | 0.72673 | 0.94349 | 0.37405 | **C205** | 0.80425 | 0.56238 | 0.69067 |
| **C22** | 0.36543 | 0.30947 | 0.01319 | **C114** | 0.14872 | 0.79976 | 0.3469 | **C206** | 0.03883 | 0.23094 | 0.69996 |
| **C23** | 0.16748 | 0.18555 | 0.03277 | **C115** | 0.45983 | 0.85263 | 0.38263 | **C207** | 0.16189 | 0.57586 | 0.63834 |
| **C24** | 0.03248 | -0.01377 | -0.00994 | **C116** | 0.69583 | 0.51299 | 0.37552 | **C208** | 0.39532 | 0.5775 | 0.66441 |
| **C25** | 0.38594 | 0.21213 | 0.00733 | **C117** | 0.81421 | 0.86113 | 0.33682 | **C209** | 0.97098 | 0.85045 | 0.5568 |
| **C26** | 0.81168 | 0.08042 | -0.00366 | **C118** | 0.04244 | 0.82794 | 0.30355 | **C210** | 0.00533 | 0.68918 | 0.67422 |
| **C27** | 0.12298 | 0.14036 | 0.04829 | **C119** | 0.62931 | 0.13077 | 0.23189 | **C211** | 0.91692 | 0.92957 | 0.63901 |
| **C28** | 0.36016 | -0.21286 | 0.01611 | **C120** | 0.66049 | 0.97447 | 0.36124 | **C212** | 0.47888 | 0.57817 | 0.68468 |
| **C29** | 0.70475 | 0.11444 | -0.03265 | **C121** | 0.57437 | 0.20636 | 0.30391 | **C213** | 0.98743 | 0.55526 | 0.69522 |
| **C30** | 0.29353 | 0.41629 | -0.10724 | **C122** | 0.12575 | 0.8348 | 0.33821 | **C214** | 0.42835 | 0.63448 | 0.66108 |
| **C31** | 0.32219 | 0.25305 | 0.01261 | **C123** | 0.64172 | 0.84556 | 0.39275 | **C215** | 0.45278 | 0.10173 | 0.65013 |
| **C32** | 0.23703 | 0.4878 | -0.03759 | **C124** | 0.07603 | 0.89716 | 0.32551 | **C216** | 0.0509 | 0.52569 | 0.71341 |
| **C33** | 0.78817 | 0.11291 | -0.01654 | **C125** | 0.10575 | 0.37788 | 0.31812 | **C217** | 0.91595 | 0.58097 | 0.68481 |
| **C34** | 0.30141 | 0.11575 | 0.02953 | **C126** | 0.70498 | 0.8181 | 0.41688 | **C218** | 0.01765 | 0.64822 | 0.68366 |
| **C35** | 0.73793 | 0.17468 | -0.02975 | **C127** | 0.57049 | 0.8689 | 0.3759 | **C219** | 0.63913 | 0.54618 | 0.70436 |
| **C36** | 0.77198 | 0.68964 | 0.03043 | **C128** | 0.67239 | 0.93472 | 0.37384 | **C220** | 0.3203 | 0.68538 | 0.67496 |
| **C37** | 0.36532 | 0.08253 | 0.04074 | **C129** | 0.29232 | 0.8254 | 0.38451 | **C221** | 0.55103 | 0.19066 | 0.66924 |
| **C38** | 0.23212 | 0.15067 | 0.03298 | **C130** | 0.97102 | 0.95388 | 0.3423 | **C222** | 0.67779 | 0.34536 | 0.62127 |
| **C39** | 0.33276 | 0.20887 | 0.01758 | **C131** | 0.20345 | 0.46255 | 0.32655 | **C223** | 0.13882 | 0.84444 | 0.64062 |
| **C40** | -0.0438 | 0.11361 | 0.04546 | **C132** | 0.33091 | 0.629 | 0.2968 | **C224** | 0.04207 | 0.5674 | 0.69953 |
| **C41** | 0.63069 | 0.22669 | -0.01781 | **C133** | 0.79241 | 0.1254 | 0.31766 | **C225** | 0.487 | 0.66223 | 0.66895 |
| **C42** | 0.86687 | 0.75851 | 0.01092 | **C134** | 0.69631 | 0.85805 | 0.3991 | **C226** | 0.90003 | 0.8527 | 0.67508 |
| **C43** | -0.0085 | -0.07224 | -0.01511 | **C135** | 0.13482 | 0.93446 | 0.35398 | **C227** | 0.08685 | 0.84719 | 0.673 |
| **C44** | 0.45383 | 0.40669 | -0.03103 | **C136** | 0.55605 | 0.13227 | 0.34583 | **C228** | 0.03745 | 0.78328 | 0.65984 |
| **C45** | 0.35527 | 0.12311 | 0.02588 | **C137** | 0.74211 | 0.13183 | 0.35906 | **C229** | 0.93805 | 0.70691 | 0.64442 |
| **C46** | 0.79666 | 0.20295 | -0.02085 | **C138** | 0.69288 | 0.06768 | 0.34547 | **C230** | 0.48959 | 0.16826 | 0.66761 |
| **C47** | 0.21754 | 0.40991 | -0.00256 | **C139** | 0.5935 | 0.9915 | 0.32887 | **C231** | 0.37309 | 0.12335 | 0.67172 |
| **C48** | 0.40483 | 0.41442 | 0.01797 | **C140** | 0.14178 | 0.43954 | 0.32496 | **C232** | 0.14594 | 0.63523 | 0.68931 |
| **C49** | 0.35575 | 0.3486 | 0.00134 | **C141** | 0.02647 | 0.40116 | 0.3425 | **C233** | 0.74133 | 0.40204 | 0.76665 |
| **C50** | 0.25629 | 0.27334 | -0.01496 | **C142** | 0.79985 | 0.91967 | 0.37957 | **C234** | 0.1843 | 0.90573 | 0.65461 |
| **C51** | 0.80599 | 0.74025 | 0.0166 | **C143** | 0.39214 | 0.68117 | 0.44085 | **C235** | 0.87055 | 0.52914 | 0.6903 |
| **C52** | 0.69134 | 0.70588 | 0.04073 | **C144** | 0.83786 | 0.18613 | 0.33058 | **C236** | -0.05346 | 0.14589 | 0.69468 |
| **C53** | 0.45779 | 0.18255 | 0.00172 | **C145** | 0.5246 | 0.81662 | 0.38007 | **C237** | 0.34293 | 0.64121 | 0.64795 |
| **C54** | 0.05802 | -0.03548 | 0.10238 | **C146** | 0.60269 | 0.41964 | 0.35395 | **C238** | 0.02867 | 0.40003 | 0.64622 |
| **C55** | 0.49863 | 0.46876 | -0.01649 | **C147** | 0.99208 | 0.90993 | 0.32037 | **C239** | 0.0854 | 0.40651 | 0.74618 |
| **C56** | 0.18678 | 0.10337 | 0.04578 | **C148** | 0.68381 | 0.69371 | 0.35243 | **C240** | 0.92927 | 0.87636 | 0.62025 |
| **C57** | 0.26703 | 0.69545 | -0.00268 | **C149** | 0.74339 | 0.70043 | 0.44797 | **C241** | 0.64569 | 0.28965 | 0.70108 |
| **C58** | 0.65284 | 0.18371 | -0.04018 | **C150** | 0.58653 | 0.15523 | 0.29054 | **C242** | 0.2223 | 0.03099 | 0.69941 |
| **C59** | 0.3455 | -0.04056 | -0.02181 | **C151** | 0.29764 | 0.56452 | 0.36393 | **C243** | 0.94603 | 0.9742 | 0.61766 |
| **C60** | 0.40569 | -0.03087 | 0.07733 | **C152** | 0.8773 | 0.31533 | 0.38453 | **C244** | 0.40985 | 0.18982 | 0.68856 |
| **C61** | 0.30839 | 0.37806 | -0.10108 | **C153** | 0.60514 | 0.25208 | 0.28444 | **C245** | -0.01871 | 0.20991 | 0.70733 |
| **C62** | 0.96133 | 0.85317 | 0.03469 | **C154** | 0.06228 | 0.46205 | 0.34763 | **C246** | 0.18959 | 0.71075 | 0.73343 |
| **C63** | 0.53933 | 0.60653 | 0.05961 | **C155** | 0.63747 | 0.4878 | 0.37573 | **C247** | 0.7089 | 0.54208 | 0.75588 |
| **C64** | 0.24907 | 0.43724 | -0.0525 | **C156** | 0.84442 | 0.98943 | 0.40866 | **C248** | 0.17939 | 0.97064 | 0.69582 |
| **C65** | 0.72523 | 0.757 | 0.02741 | **C157** | 0.36782 | 0.844 | 0.46171 | **C249** | 0.09166 | 0.57005 | 0.80281 |
| **C66** | 0.30159 | -0.23958 | 0.01199 | **C158** | 0.83458 | 0.25458 | 0.38047 | **C250** | -0.07269 | 0.03023 | 0.6559 |
| **C67** | 0.50252 | 0.25767 | 0.04651 | **C159** | 0.74741 | 0.86324 | 0.50437 | **C251** | 0.3119 | 0.10396 | 0.67818 |
| **C68** | 0.03272 | 0.1403 | 0.1327 | **C160** | 0.58467 | 0.30336 | 0.31217 | **C252** | 0.67997 | 0.57048 | 0.65692 |
| **C69** | 0.49636 | 0.54127 | 0.04684 | **C161** | 0.96628 | 0.38622 | 0.35769 | **C253** | 0.05567 | 0.33004 | 0.69849 |
| **C70** | 0.40784 | 0.12888 | 0.12906 | **C162** | 0.33084 | 0.84149 | 0.32606 | **C254** | 0.46718 | 0.2119 | 0.6865 |
| **C71** | 0.25154 | 0.59265 | -0.01541 | **C163** | 0.71441 | 0.62248 | 0.39565 | **C255** | 0.27426 | 0.039 | 0.67021 |
| **C72** | 0.63048 | 0.68742 | 0.05005 | **C164** | 0.11893 | 0.48092 | 0.33899 | **C256** | 0.06053 | 0.1859 | 0.6785 |
| **C73** | -0.0065 | 0.12186 | -0.0214 | **C165** | 0.92839 | 0.32148 | 0.35048 | **C257** | 0.51191 | 0.6343 | 0.67995 |
| **C74** | 0.37744 | -0.10798 | 0.02726 | **C166** | 0.7177 | 0.46568 | 0.34721 | **C258** | 0.26115 | 0.66632 | 0.67658 |
| **C75** | 0.78157 | 0.77401 | 0.01582 | **C167** | 0.1594 | 0.9041 | 0.36051 | **C259** | 0.74857 | 0.55969 | 0.69992 |
| **C76** | 0.59288 | 0.62015 | 0.03821 | **C168** | 0.91303 | 0.94016 | 0.35147 | **C260** | 0.56042 | 0.55876 | 0.68397 |
| **C77** | 0.38251 | 0.74354 | -0.00309 | **C169** | 0.40433 | 0.85077 | 0.39274 | **C261** | 0.77196 | 0.45825 | 0.69207 |
| **C78** | 0.82142 | 0.17256 | -0.01471 | **C170** | 0.20928 | 0.82122 | 0.34378 | **C262** | 0.91542 | 0.81267 | 0.67656 |
| **C79** | 0.57177 | 0.20826 | -0.01759 | **C171** | 0.42455 | 0.74451 | 0.37674 | **C263** | 0.4203 | 0.55042 | 0.6768 |
| **C80** | 0.06792 | 0.1393 | 0.05651 | **C172** | 0.57121 | 0.09465 | 0.3528 | **C264** | 0.08409 | 0.91561 | 0.72292 |
| **C81** | 0.87217 | 0.101 | -0.00816 | **C173** | 0.06698 | 0.79762 | 0.31014 | **C265** | 0.98541 | 0.80889 | 0.55647 |
| **C82** | 0.08742 | 0.0369 | 0.05296 | **C174** | 0.74086 | 0.20337 | 0.41717 | **C266** | 0.96033 | 0.79322 | 0.62222 |
| **C83** | 0.23346 | 0.37379 | 0.00608 | **C175** | 0.6437 | 0.0918 | 0.23719 | **C267** | 0.68788 | 0.34642 | 0.6971 |
| **C84** | 0.72949 | 0.08452 | -0.02568 | **C176** | 0.617 | 0.07655 | 0.30303 | **C268** | 0.40431 | 0.66996 | 0.65564 |
| **C85** | 0.4041 | 0.48849 | 0.08606 | **C177** | 0.3397 | 0.6254 | 0.36766 | **C269** | 0.58273 | 0.53752 | 0.70938 |
| **C86** | 0.28069 | 0.35881 | -0.03993 | **C178** | 0.05275 | 0.93434 | 0.32186 | **C270** | 0.81556 | 0.51775 | 0.69177 |
| **C87** | 0.00335 | -0.08533 | 0.03976 | **C179** | 0.23332 | 0.80424 | 0.37406 | **O13** | 0.29493 | 0.14364 | 0.6917 |
| **C88** | 0.71424 | 0.21271 | -0.03109 | **C180** | 0.46979 | 0.80597 | 0.38114 | **O14** | 0.47236 | 0.51689 | 0.73581 |
| **C89** | -0.10276 | 0.09255 | 0.03482 | **O7** | 0.95011 | 0.42876 | 0.37701 | **O15** | 0.12897 | 0.33968 | 0.76054 |
| **C90** | 0.13251 | 0.09607 | 0.05268 | **O8** | 0.11645 | 0.75506 | 0.35861 | **O16** | -0.12082 | 0.02345 | 0.68026 |
| **O1** | 0.61293 | 0.727 | 0.06483 | **O9** | 0.78341 | 0.6328 | 0.46944 | **O17** | 0.43632 | 0.72408 | 0.66061 |
| **O2** | 0.7805 | 0.03917 | 0.01403 | **O10** | 0.53507 | 0.29149 | 0.32641 | **O18** | 0.56882 | 0.15704 | 0.66833 |
| **O3** | 0.44859 | -0.09554 | 0.09957 | **O11** | 0.08576 | 0.98718 | 0.32465 | **C182** | -0.05105 | 0.67256 | 0.66475 |
| **O4** | 0.20577 | 0.59257 | 0.02019 | **O12** | 0.22162 | 0.43006 | 0.32777 | **C186** | -0.02653 | 0.59434 | 0.68796 |
| **O5** | 0.74693 | 0.26899 | -0.02121 | **C99** | 0.63542 | 1.04508 | 0.32462 | **C189** | -0.0203 | 0.76088 | 0.64063 |
| **O6** | 0.88473 | 0.72822 | 0.01738 | **C110** | 0.70314 | 1.03054 | 0.3607 | **C210** | 1.00533 | 0.68918 | 0.67422 |
| **C28** | 0.36016 | 0.78714 | 0.01611 | **C120** | 0.66049 | -0.02553 | 0.36124 | **C213** | -0.01257 | 0.55526 | 0.69522 |
| **C57** | 0.26703 | -0.30455 | -0.00268 | **C139** | 0.5935 | -0.0085 | 0.32887 | **C218** | 1.01765 | 0.64822 | 0.68366 |
| **C62** | -0.03867 | -0.14683 | 0.03469 | **C141** | 1.02647 | 0.40116 | 0.3425 | **C224** | 1.04207 | 0.5674 | 0.69953 |
| **C66** | 0.30159 | 0.76042 | 0.01199 | **C147** | -0.00792 | 0.90993 | 0.32037 | **C228** | 1.03745 | 0.78328 | 0.65984 |
| **C77** | 0.38251 | -0.25646 | -0.00309 | **C161** | -0.03372 | 0.38622 | 0.35769 | **C242** | 0.2223 | 1.03099 | 0.69941 |
| **C81** | -0.12783 | 0.101 | -0.00816 | **C178** | 1.05275 | 0.93434 | 0.32186 | **C243** | -0.05397 | -0.0258 | 0.61766 |
| **C87** | 1.00335 | 0.91467 | 0.03976 | **H106** | 0.67004 | 0.58312 | 0.61922 | **C248** | 0.17939 | -0.02936 | 0.69582 |
| **C89** | 0.89724 | 0.09255 | 0.03482 | **H107** | 0.39515 | 0.50759 | 0.68178 | **C250** | 0.92731 | 1.03023 | 0.6559 |
| **H52** | 0.31718 | 0.84033 | 0.27207 | **H108** | 0.72034 | 0.53592 | 0.80184 |  |  |  |  |
| **H53** | 0.04117 | 0.74441 | 0.29406 | **H109** | 0.1434 | 0.79222 | 0.59969 |  |  |  |  |


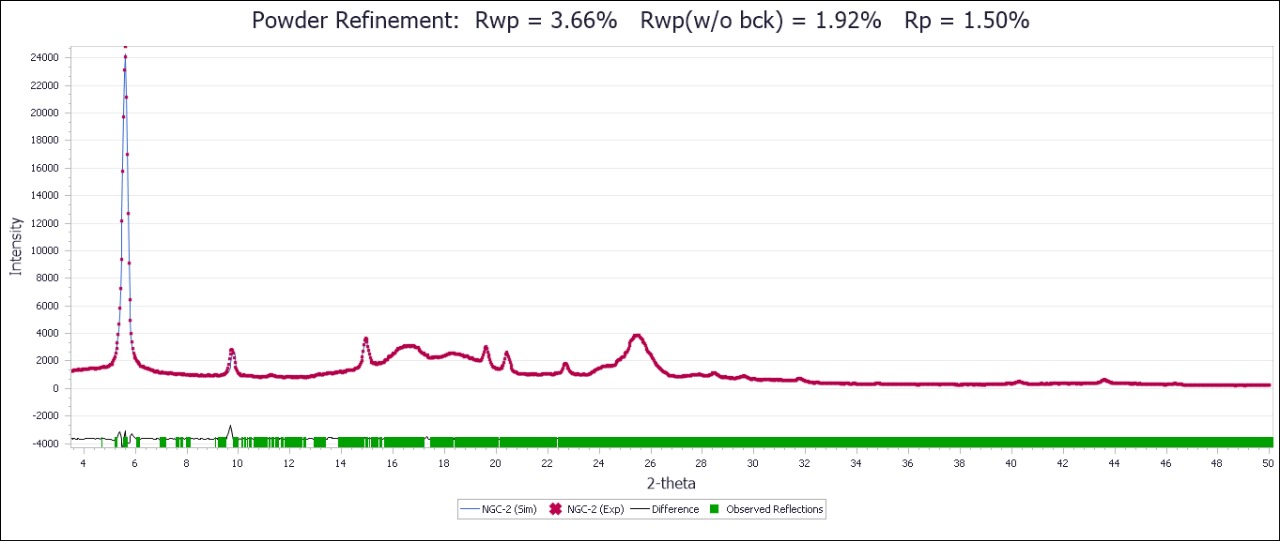


Figure S5: Pawley refinement of NGC-2. The experimental PXRD pattern after Pawley refinement is in good agreement with the simulated one.

Table S3. Fractional atomic coordinates of a unit cell and lattice parameters of NGC-2.

| **NGC-2 ABC model; Space group P1**  **a** =33.39 **Å, b** =33.38 **Å, c =** 18.83 **Å**; **α=** 90.83 **°,β** =88.47**°,γ** =119.25**°** | | | | | | | | | | | |
| --- | --- | --- | --- | --- | --- | --- | --- | --- | --- | --- | --- |
| **Atoms** | X | Y | Z | **Atoms** | X | Y | Z | **Atoms** | X | Y | Z |
| **C1** | 0.48256 | 0.44892 | 0.61027 | **C181** | 1.17493 | 0.75933 | 0.95804 | **C361** | 0.83305 | 1.11135 | 1.29055 |
| **C2** | 0.527 | 0.45417 | 0.61133 | **C182** | 1.21937 | 0.76458 | 0.9591 | **C362** | 0.87749 | 1.11659 | 1.2916 |
| **C3** | 0.4789 | 0.48904 | 0.6107 | **C183** | 1.17128 | 0.79945 | 0.95847 | **C363** | 0.82939 | 1.15146 | 1.29097 |
| **C4** | 0.56634 | 0.49792 | 0.61276 | **C184** | 1.25872 | 0.80833 | 0.96053 | **C364** | 0.91683 | 1.16034 | 1.29303 |
| **C5** | 0.51823 | 0.53281 | 0.61213 | **C185** | 1.21061 | 0.84322 | 0.9599 | **C365** | 0.86872 | 1.19523 | 1.2924 |
| **C6** | 0.56215 | 0.53745 | 0.61316 | **C186** | 1.25453 | 0.84786 | 0.96093 | **C366** | 0.91264 | 1.19988 | 1.29344 |
| **C7** | 0.43909 | 0.40058 | 0.60869 | **C187** | 1.13147 | 0.71099 | 0.95646 | **C367** | 0.78958 | 1.063 | 1.28897 |
| **C8** | 0.43073 | 0.48459 | 0.60957 | **C188** | 1.12311 | 0.79501 | 0.95734 | **C368** | 0.78122 | 1.14702 | 1.28984 |
| **C9** | 0.53218 | 0.41087 | 0.6109 | **C189** | 1.22456 | 0.72128 | 0.95866 | **C369** | 0.88267 | 1.07329 | 1.29117 |
| **C10** | 0.61455 | 0.50246 | 0.61389 | **C190** | 1.30693 | 0.81288 | 0.96166 | **C370** | 0.96504 | 1.16489 | 1.29416 |
| **C11** | 0.60969 | 0.58248 | 0.61474 | **C191** | 1.30207 | 0.8929 | 0.96251 | **C371** | 0.96018 | 1.24491 | 1.29502 |
| **C12** | 0.5171 | 0.57277 | 0.61256 | **C192** | 1.20948 | 0.88319 | 0.96033 | **C372** | 0.86759 | 1.2352 | 1.29283 |
| **C13** | 0.38803 | 0.43685 | 0.60802 | **C193** | 1.0804 | 0.74726 | 0.95579 | **C373** | 0.73852 | 1.09927 | 1.28829 |
| **C14** | 0.39324 | 0.39623 | 0.60762 | **C194** | 1.08562 | 0.70664 | 0.95539 | **C374** | 0.74373 | 1.05865 | 1.28789 |
| **C15** | 0.55902 | 0.62493 | 0.6107 | **C195** | 1.2514 | 0.93534 | 0.95847 | **C375** | 0.90951 | 1.28735 | 1.29097 |
| **C16** | 0.61624 | 0.63028 | 0.61345 | **C196** | 1.30862 | 0.94069 | 0.96122 | **C376** | 0.96673 | 1.2927 | 1.29372 |
| **C17** | 0.61609 | 0.46602 | 0.61346 | **C197** | 1.30847 | 0.77643 | 0.96123 | **C377** | 0.96658 | 1.12845 | 1.29373 |
| **C18** | 0.57835 | 0.41937 | 0.61203 | **C198** | 1.27073 | 0.72979 | 0.9598 | **C378** | 0.92884 | 1.0818 | 1.2923 |
| **C19** | 0.24861 | 0.2117 | 0.60919 | **C199** | 0.94098 | 0.52211 | 0.95696 | **C379** | 0.5991 | 0.87413 | 1.28947 |
| **H20** | 0.32298 | 0.37046 | 0.54926 | **H200** | 1.01536 | 0.68087 | 0.89703 | **H380** | 0.67347 | 1.03289 | 1.22953 |
| **H21** | 0.27214 | 0.19636 | 0.61275 | **H201** | 0.96452 | 0.50677 | 0.96052 | **H381** | 0.62263 | 0.85878 | 1.29302 |
| **O22** | 0.8257 | 0.88748 | 0.537 | **O202** | 1.51808 | 1.19789 | 0.88478 | **O382** | 1.17619 | 1.5499 | 1.21728 |
| **O23** | 0.20423 | 0.1121 | 0.62144 | **O203** | 0.8966 | 0.42251 | 0.96921 | **O383** | 0.55472 | 0.77452 | 1.30172 |
| **C24** | 0.77824 | 0.80319 | 0.58168 | **C204** | 1.47062 | 1.1136 | 0.92945 | **C384** | 1.12873 | 1.46561 | 1.26195 |
| **H25** | 0.75397 | 0.81354 | 0.55389 | **H205** | 1.44635 | 1.12395 | 0.90166 | **H385** | 1.10446 | 1.47596 | 1.23416 |
| **C26** | 0.6558 | 0.66825 | 0.59946 | **C206** | 1.34818 | 0.97866 | 0.94723 | **C386** | 1.00629 | 1.33067 | 1.27973 |
| **C27** | 0.69498 | 0.67177 | 0.56733 | **C207** | 1.38735 | 0.98218 | 0.9151 | **C387** | 1.04547 | 1.33419 | 1.2476 |
| **C28** | 0.65955 | 0.70761 | 0.63212 | **C208** | 1.35193 | 1.01802 | 0.97988 | **C388** | 1.01004 | 1.37003 | 1.31239 |
| **C29** | 0.73546 | 0.7144 | 0.56068 | **C209** | 1.42784 | 1.02481 | 0.90845 | **C389** | 1.08595 | 1.37682 | 1.24095 |
| **H30** | 0.69428 | 0.63952 | 0.54598 | **H210** | 1.38666 | 0.94993 | 0.89375 | **H390** | 1.04477 | 1.30194 | 1.22626 |
| **C31** | 0.70053 | 0.75023 | 0.62775 | **C211** | 1.39291 | 1.06064 | 0.97551 | **C391** | 1.05102 | 1.41266 | 1.30802 |
| **H32** | 0.62863 | 0.70469 | 0.66219 | **H212** | 1.32101 | 1.0151 | 1.00996 | **H392** | 0.97912 | 1.36711 | 1.34246 |
| **C33** | 0.73842 | 0.75414 | 0.59059 | **C213** | 1.4308 | 1.06455 | 0.93837 | **C393** | 1.08891 | 1.41656 | 1.27087 |
| **H34** | 0.7663 | 0.71697 | 0.5308 | **H214** | 1.45868 | 1.02738 | 0.87857 | **H394** | 1.11679 | 1.37939 | 1.21108 |
| **H35** | 0.70297 | 0.78202 | 0.65425 | **H215** | 1.39535 | 1.09244 | 1.00202 | **H395** | 1.05346 | 1.44445 | 1.33452 |
| **C36** | 0.82196 | 0.82766 | 0.59288 | **C216** | 1.51434 | 1.13807 | 0.94065 | **C396** | 1.17245 | 1.49009 | 1.27315 |
| **C37** | 0.84756 | 0.87136 | 0.56979 | **C217** | 1.53994 | 1.18177 | 0.91756 | **C397** | 1.19805 | 1.53378 | 1.25007 |
| **H38** | 0.84287 | 0.81673 | 0.6273 | **H218** | 1.53525 | 1.12715 | 0.97507 | **H398** | 1.19336 | 1.47916 | 1.30758 |
| **C39** | 0.89449 | 0.9112 | 0.56626 | **C219** | 1.58687 | 1.22161 | 0.91403 | **C399** | 1.24498 | 1.57362 | 1.24654 |
| **C40** | 0.90551 | 0.95582 | 0.54463 | **C220** | 1.59789 | 1.26624 | 0.8924 | **C400** | 1.256 | 1.61825 | 1.2249 |
| **C41** | 0.93028 | 0.90363 | 0.58827 | **C221** | 1.62266 | 1.21405 | 0.93604 | **C401** | 1.28077 | 1.56606 | 1.26855 |
| **C42** | 0.95132 | 0.99217 | 0.54671 | **C222** | 1.6437 | 1.30258 | 0.89447 | **C402** | 1.30181 | 1.6546 | 1.22698 |
| **H43** | 0.87403 | 0.96523 | 0.52015 | **H223** | 1.56641 | 1.27564 | 0.86792 | **H403** | 1.22452 | 1.62766 | 1.20042 |
| **C44** | 0.9761 | 0.93986 | 0.58945 | **C224** | 1.66848 | 1.25027 | 0.93722 | **C404** | 1.32659 | 1.60228 | 1.26973 |
| **H45** | 0.92162 | 0.86753 | 0.60581 | **H225** | 1.61399 | 1.17794 | 0.95358 | **H405** | 1.27211 | 1.52996 | 1.28608 |
| **C46** | 0.98691 | 0.9844 | 0.56936 | **C226** | 1.67929 | 1.29481 | 0.91713 | **C406** | 1.3374 | 1.64683 | 1.24964 |
| **H47** | 0.96352 | 1.02821 | 0.53188 | **H227** | 1.65589 | 1.33863 | 0.87965 | **H407** | 1.31401 | 1.69064 | 1.21215 |
| **H48** | 1.003 | 0.93298 | 0.6062 | **H228** | 1.69538 | 1.24339 | 0.95396 | **H408** | 1.35349 | 1.59541 | 1.28647 |
| **C49** | 0.18186 | 0.13069 | 0.60061 | **C229** | 0.87424 | 0.4411 | 0.94838 | **C409** | 0.53235 | 0.79311 | 1.28089 |
| **C50** | 0.20234 | 0.18182 | 0.59889 | **C230** | 0.89472 | 0.49223 | 0.94666 | **C410** | 0.55283 | 0.84424 | 1.27917 |
| **H51** | 0.17835 | 0.19654 | 0.60653 | **H231** | 0.87073 | 0.50695 | 0.9543 | **H411** | 0.52884 | 0.85896 | 1.28681 |
| **C52** | 0.28183 | 0.25775 | 0.60528 | **C232** | 0.9742 | 0.56816 | 0.95305 | **C412** | 0.63232 | 0.92017 | 1.28555 |
| **C53** | 0.31944 | 0.26521 | 0.64672 | **C233** | 1.01181 | 0.57563 | 0.99449 | **C413** | 0.66993 | 0.92764 | 1.327 |
| **C54** | 0.28301 | 0.29591 | 0.57246 | **C234** | 0.97539 | 0.60632 | 0.92023 | **C414** | 0.6335 | 0.95833 | 1.25274 |
| **C55** | 0.35945 | 0.30849 | 0.64923 | **C235** | 1.05183 | 0.61891 | 0.997 | **C415** | 0.70994 | 0.97092 | 1.32951 |
| **H56** | 0.31782 | 0.23573 | 0.67928 | **H236** | 1.0102 | 0.54614 | 1.02705 | **H416** | 0.66831 | 0.89815 | 1.35956 |
| **C57** | 0.32244 | 0.33979 | 0.5766 | **C237** | 1.01482 | 0.6502 | 0.92437 | **C417** | 0.67293 | 1.00221 | 1.25687 |
| **H58** | 0.25144 | 0.29085 | 0.54177 | **H238** | 0.94382 | 0.60126 | 0.88954 | **H418** | 0.60193 | 0.95328 | 1.22204 |
| **C59** | 0.36133 | 0.34617 | 0.61367 | **C239** | 1.05371 | 0.65658 | 0.96144 | **C419** | 0.71182 | 1.00859 | 1.29394 |
| **H60** | 0.39079 | 0.31329 | 0.68033 | **H240** | 1.08317 | 0.62371 | 1.0281 | **H420** | 0.74128 | 0.97572 | 1.3606 |
| **C61** | 0.57663 | 0.24861 | 0.60937 | **C241** | 1.26901 | 0.55902 | 0.95714 | **C421** | 0.92712 | 0.91103 | 1.28964 |
| **H62** | 0.5006 | 0.33055 | 0.57885 | **H242** | 1.19298 | 0.64096 | 0.92662 | **H422** | 0.85109 | 0.99297 | 1.25913 |
| **H63** | 0.61167 | 0.26864 | 0.59178 | **H243** | 1.30405 | 0.57905 | 0.93955 | **H423** | 0.96216 | 0.93107 | 1.27206 |
| **O64** | 0.4861 | 0.82041 | 0.67034 | **O244** | 1.17848 | 1.13082 | 1.01811 | **O424** | 0.83659 | 1.48283 | 1.35062 |
| **O65** | 0.61533 | 0.1938 | 0.55747 | **O245** | 1.30771 | 0.50421 | 0.90524 | **O425** | 0.96582 | 0.85622 | 1.23774 |
| **C66** | 0.54263 | 0.78793 | 0.63454 | **C246** | 1.23501 | 1.09834 | 0.98231 | **C426** | 0.89312 | 1.45035 | 1.31481 |
| **H67** | 0.50581 | 0.7591 | 0.64925 | **H247** | 1.19818 | 1.06951 | 0.99702 | **H427** | 0.8563 | 1.42153 | 1.32953 |
| **C68** | 0.55291 | 0.66688 | 0.61656 | **C248** | 1.24529 | 0.97729 | 0.96433 | **C428** | 0.9034 | 1.3293 | 1.29684 |
| **C69** | 0.51344 | 0.667 | 0.64009 | **C249** | 1.20582 | 0.97741 | 0.98786 | **C429** | 0.86393 | 1.32942 | 1.32037 |
| **C70** | 0.59032 | 0.7086 | 0.59301 | **C250** | 1.2827 | 1.01901 | 0.94078 | **C430** | 0.94081 | 1.37102 | 1.27328 |
| **C71** | 0.51289 | 0.70827 | 0.64766 | **C251** | 1.20527 | 1.01868 | 0.99543 | **C431** | 0.86338 | 1.37069 | 1.32794 |
| **H72** | 0.48093 | 0.63303 | 0.6535 | **H252** | 1.17331 | 0.94345 | 1.00127 | **H432** | 0.83142 | 1.29546 | 1.33377 |
| **C73** | 0.5895 | 0.74998 | 0.59821 | **C253** | 1.28188 | 1.06039 | 0.94598 | **C433** | 0.93999 | 1.4124 | 1.27849 |
| **H74** | 0.62182 | 0.70863 | 0.56975 | **H254** | 1.31419 | 1.01904 | 0.91752 | **H434** | 0.97231 | 1.37106 | 1.25002 |
| **C75** | 0.55119 | 0.75025 | 0.6271 | **C255** | 1.24357 | 1.06066 | 0.97487 | **C435** | 0.90168 | 1.41267 | 1.30737 |
| **H76** | 0.48116 | 0.7079 | 0.67067 | **H256** | 1.17354 | 1.01831 | 1.01844 | **H436** | 0.83165 | 1.37032 | 1.35094 |
| **H77** | 0.62033 | 0.78371 | 0.5792 | **H257** | 1.31271 | 1.09412 | 0.92697 | **H437** | 0.97082 | 1.44613 | 1.25947 |
| **C78** | 0.55424 | 0.83253 | 0.62551 | **C258** | 1.24662 | 1.14295 | 0.97328 | **C438** | 0.90473 | 1.49496 | 1.30578 |
| **C79** | 0.52469 | 0.84747 | 0.64286 | **C259** | 1.21707 | 1.15788 | 0.99063 | **C439** | 0.87518 | 1.50989 | 1.32314 |
| **H80** | 0.58995 | 0.85864 | 0.60459 | **H260** | 1.28232 | 1.16905 | 0.95236 | **H440** | 0.94043 | 1.52106 | 1.28487 |
| **C81** | 0.54487 | 0.90362 | 0.61636 | **C261** | 1.23725 | 1.21403 | 0.96413 | **C441** | 0.89536 | 1.56604 | 1.29663 |
| **C82** | 0.53016 | 0.92108 | 0.67204 | **C262** | 1.22254 | 1.2315 | 1.01981 | **C442** | 0.88065 | 1.58351 | 1.35231 |
| **C83** | 0.56537 | 0.93147 | 0.55672 | **C263** | 1.25775 | 1.24188 | 0.90449 | **C443** | 0.91586 | 1.5939 | 1.237 |
| **C84** | 0.53694 | 0.96592 | 0.66846 | **C264** | 1.22932 | 1.27633 | 1.01623 | **C444** | 0.88743 | 1.62834 | 1.34874 |
| **H85** | 0.51293 | 0.89996 | 0.71765 | **H265** | 1.20531 | 1.21037 | 1.06542 | **H445** | 0.86342 | 1.56238 | 1.39793 |
| **C86** | 0.57158 | 0.97604 | 0.55285 | **C266** | 1.26396 | 1.28645 | 0.90062 | **C446** | 0.92207 | 1.63846 | 1.23312 |
| **H87** | 0.57733 | 0.91753 | 0.51133 | **H267** | 1.26971 | 1.22794 | 0.8591 | **H447** | 0.92782 | 1.57995 | 1.19161 |
| **C88** | 0.55785 | 0.99374 | 0.60883 | **C268** | 1.25023 | 1.30415 | 0.9566 | **C448** | 0.90834 | 1.65616 | 1.2891 |
| **H89** | 0.52547 | 0.9801 | 0.71416 | **H269** | 1.21785 | 1.29051 | 1.06193 | **H449** | 0.87596 | 1.64253 | 1.39443 |
| **H90** | 0.58719 | 0.99661 | 0.5061 | **H270** | 1.27957 | 1.30702 | 0.85386 | **H450** | 0.93768 | 1.65903 | 1.18637 |
| **C91** | 0.57872 | 0.17455 | 0.59254 | **C271** | 1.2711 | 0.48496 | 0.94031 | **C451** | 0.92921 | 0.83697 | 1.27282 |
| **C92** | 0.55819 | 0.20296 | 0.62129 | **C272** | 1.25057 | 0.51338 | 0.96906 | **C452** | 0.90868 | 0.86539 | 1.30156 |
| **H93** | 0.52874 | 0.18576 | 0.6611 | **H273** | 1.22112 | 0.49617 | 1.00887 | **H453** | 0.87923 | 0.84819 | 1.34137 |
| **C94** | 0.57785 | 0.29296 | 0.61757 | **C274** | 1.27023 | 0.60337 | 0.96534 | **C454** | 0.92834 | 0.95538 | 1.29784 |
| **C95** | 0.61451 | 0.33478 | 0.64125 | **C275** | 1.30689 | 0.64519 | 0.98902 | **C455** | 0.965 | 0.9972 | 1.32153 |
| **C96** | 0.53689 | 0.29168 | 0.59742 | **C276** | 1.22927 | 0.60209 | 0.94519 | **C456** | 0.88738 | 0.9541 | 1.27769 |
| **C97** | 0.61258 | 0.3755 | 0.63757 | **C277** | 1.30496 | 0.68591 | 0.98534 | **C457** | 0.96307 | 1.03792 | 1.31784 |
| **H98** | 0.64673 | 0.33611 | 0.66408 | **H278** | 1.33911 | 0.64652 | 1.01185 | **H458** | 0.99722 | 0.99853 | 1.34435 |
| **C99** | 0.53409 | 0.3321 | 0.59577 | **C279** | 1.22647 | 0.64251 | 0.94354 | **C459** | 0.88458 | 0.99452 | 1.27604 |
| **H100** | 0.50567 | 0.25721 | 0.58185 | **H280** | 1.19805 | 0.56762 | 0.92962 | **H460** | 0.85616 | 0.91964 | 1.26212 |
| **C101** | 0.57228 | 0.37447 | 0.61429 | **C281** | 1.26466 | 0.68488 | 0.96206 | **C461** | 0.92277 | 1.03689 | 1.29456 |
| **H102** | 0.64389 | 0.40979 | 0.65356 | **H282** | 1.33626 | 0.7202 | 1.00133 | **H462** | 0.99438 | 1.07221 | 1.33384 |
| **H103** | 0.96413 | 0.53141 | 0.58855 | **H283** | 1.65651 | 0.84182 | 0.93632 | **H463** | 1.31462 | 1.19383 | 1.26882 |
| **C104** | 0.92465 | 0.40146 | 0.61424 | **C284** | 1.61703 | 0.71187 | 0.96201 | **C464** | 1.27514 | 1.06389 | 1.29452 |
| **H105** | 0.85319 | 0.38838 | 0.61677 | **H285** | 1.54557 | 0.69879 | 0.96454 | **H465** | 1.20368 | 1.0508 | 1.29704 |
| **C106** | 0.97163 | 0.43424 | 0.60953 | **C286** | 1.66401 | 0.74465 | 0.9573 | **C466** | 1.32212 | 1.09666 | 1.2898 |
| **H107** | 1.02331 | 0.50792 | 0.59673 | **H287** | 1.71569 | 0.81834 | 0.9445 | **H467** | 1.3738 | 1.17035 | 1.27701 |
| **H108** | 0.91354 | 0.36521 | 0.61996 | **H288** | 1.60592 | 0.67562 | 0.96773 | **H468** | 1.26403 | 1.02763 | 1.30024 |
| **C109** | 0.14297 | 0.41739 | 0.61374 | **C289** | 0.83535 | 0.72781 | 0.96151 | **C469** | 0.49346 | 1.07982 | 1.29402 |
| **C110** | 0.19382 | 0.44175 | 0.6167 | **C290** | 0.8862 | 0.75217 | 0.96447 | **C470** | 0.54431 | 1.10418 | 1.29697 |
| **H111** | 0.21276 | 0.48107 | 0.62034 | **H291** | 0.90514 | 0.79148 | 0.96811 | **H471** | 0.56325 | 1.1435 | 1.30061 |
| **C112** | 0.26907 | 0.43966 | 0.62033 | **C292** | 0.96145 | 0.75007 | 0.9681 | **C472** | 0.61956 | 1.10208 | 1.3006 |
| **C113** | 0.27419 | 0.40798 | 0.66171 | **C293** | 0.96656 | 0.71839 | 1.00948 | **C473** | 0.62468 | 1.07041 | 1.34198 |
| **C114** | 0.30723 | 0.47211 | 0.58136 | **C294** | 0.99961 | 0.78252 | 0.92913 | **C474** | 0.65772 | 1.13454 | 1.26164 |
| **C115** | 0.34826 | 0.47046 | 0.57961 | **C295** | 1.04064 | 0.78087 | 0.92738 | **C475** | 0.69875 | 1.13288 | 1.25988 |
| **H116** | 0.30449 | 0.49996 | 0.5505 | **H296** | 0.99687 | 0.81037 | 0.89827 | **H476** | 0.65498 | 1.16238 | 1.23078 |
| **C117** | 0.35175 | 0.43626 | 0.61686 | **C297** | 1.04413 | 0.74667 | 0.96463 | **C477** | 0.70224 | 1.09869 | 1.29713 |
| **C118** | 0.31439 | 0.40509 | 0.65846 | **C298** | 1.00677 | 0.7155 | 1.00623 | **C478** | 0.66488 | 1.06752 | 1.33873 |
| **H119** | 0.24527 | 0.38391 | 0.69896 | **H299** | 0.93765 | 0.69432 | 1.04673 | **H479** | 0.59576 | 1.04634 | 1.37923 |
| **H120** | 0.31692 | 0.37727 | 0.68975 | **H300** | 1.0093 | 0.68768 | 1.03752 | **H480** | 0.66741 | 1.0397 | 1.37002 |
| **C121** | 0.7946 | 0.46915 | 0.6066 | **C301** | 1.48698 | 0.77956 | 0.95437 | **C481** | 1.14509 | 1.13158 | 1.28687 |
| **H122** | 0.81495 | 0.50998 | 0.61258 | **H302** | 1.50732 | 0.82039 | 0.96035 | **H482** | 1.16544 | 1.17241 | 1.29286 |
| **C123** | 0.66145 | 0.46068 | 0.60346 | **C303** | 1.35383 | 0.7711 | 0.95123 | **C483** | 1.01194 | 1.12311 | 1.28374 |
| **C124** | 0.66593 | 0.42407 | 0.57757 | **C304** | 1.35831 | 0.73448 | 0.92534 | **C484** | 1.01642 | 1.08649 | 1.25784 |
| **C125** | 0.69975 | 0.49743 | 0.63638 | **C305** | 1.39213 | 0.80785 | 0.98415 | **C485** | 1.05024 | 1.15986 | 1.31665 |
| **C126** | 0.70869 | 0.42614 | 0.57707 | **C306** | 1.40107 | 0.73655 | 0.92484 | **C486** | 1.05918 | 1.08856 | 1.25735 |
| **H127** | 0.63437 | 0.39177 | 0.55636 | **H307** | 1.32675 | 0.70218 | 0.90413 | **H487** | 0.98486 | 1.05419 | 1.23663 |
| **C128** | 0.74244 | 0.49909 | 0.63822 | **C308** | 1.43482 | 0.8095 | 0.98599 | **C488** | 1.09293 | 1.16152 | 1.31849 |
| **H129** | 0.69593 | 0.52636 | 0.6615 | **H309** | 1.3883 | 0.83677 | 1.00927 | **H489** | 1.04642 | 1.18879 | 1.34177 |
| **C130** | 0.74747 | 0.46396 | 0.60713 | **C310** | 1.43984 | 0.77438 | 0.9549 | **C490** | 1.09796 | 1.12639 | 1.2874 |
| **H131** | 0.71216 | 0.39696 | 0.55213 | **H311** | 1.40454 | 0.70737 | 0.8999 | **H491** | 1.06265 | 1.05938 | 1.2324 |
| **H132** | 0.77338 | 0.52937 | 0.66484 | **H312** | 1.46576 | 0.83978 | 1.01261 | **H492** | 1.12387 | 1.19179 | 1.34511 |
| **C133** | 0.82241 | 0.45084 | 0.59961 | **C313** | 1.51479 | 0.76126 | 0.94738 | **C493** | 1.1729 | 1.11327 | 1.27989 |
| **C134** | 0.8688 | 0.47803 | 0.59624 | **C314** | 1.56118 | 0.78845 | 0.944 | **C494** | 1.21929 | 1.14046 | 1.27651 |
| **H135** | 0.80704 | 0.41176 | 0.60157 | **H315** | 1.49942 | 0.72217 | 0.94934 | **H495** | 1.15753 | 1.07418 | 1.28184 |
| **C136** | 0.2201 | 0.42213 | 0.62315 | **C316** | 0.91248 | 0.73254 | 0.97092 | **C496** | 0.57059 | 1.08456 | 1.30342 |
| **H137** | 0.37893 | 0.49695 | 0.54732 | **H317** | 1.07131 | 0.80736 | 0.89509 | **H497** | 0.72942 | 1.15937 | 1.22759 |
| **H138** | 0.20888 | 0.3869 | 0.62066 | **H318** | 0.90126 | 0.69731 | 0.96843 | **H498** | 0.55937 | 1.04932 | 1.30094 |
| **O139** | 0.88877 | 0.52101 | 0.59641 | **O319** | 1.58114 | 0.83142 | 0.94418 | **O499** | 1.23925 | 1.18343 | 1.27668 |
| **O140** | 0.12006 | 0.37502 | 0.62629 | **O320** | 0.81244 | 0.68543 | 0.97406 | **O500** | 0.47055 | 1.03744 | 1.30657 |
| **C141** | 0.90456 | 0.46196 | 0.60377 | **C321** | 1.59693 | 0.77238 | 0.95154 | **C501** | 1.25505 | 1.12439 | 1.28404 |
| **C142** | 0.95179 | 0.49482 | 0.59753 | **C322** | 1.64417 | 0.80523 | 0.9453 | **C502** | 1.30228 | 1.15725 | 1.2778 |
| **C143** | 0.89142 | 0.41522 | 0.61176 | **C323** | 1.5838 | 0.72563 | 0.95953 | **C503** | 1.24191 | 1.07765 | 1.29204 |
| **C144** | 0.98503 | 0.48106 | 0.60094 | **C324** | 1.67741 | 0.79147 | 0.94871 | **C504** | 1.33552 | 1.14348 | 1.28121 |
| **H145** | 0.06741 | 0.506 | 0.59765 | **H325** | 0.75979 | 0.81642 | 0.94542 | **H505** | 0.4179 | 1.16843 | 1.27792 |
| **C146** | 0.05145 | 0.38271 | 0.60828 | **C326** | 0.74383 | 0.69312 | 0.95605 | **C506** | 0.40194 | 1.04513 | 1.28855 |
| **H147** | -0.02359 | 0.35615 | 0.61032 | **H327** | 0.66879 | 0.66656 | 0.95809 | **H507** | 0.3269 | 1.01857 | 1.29059 |
| **C148** | 0.09575 | 0.42149 | 0.6068 | **C328** | 0.78812 | 0.7319 | 0.95456 | **C508** | 0.44623 | 1.08391 | 1.28707 |
| **H149** | 0.13665 | 0.49746 | 0.60209 | **H329** | 0.82903 | 0.80788 | 0.94986 | **H509** | 0.48714 | 1.15989 | 1.28236 |
| **H150** | 0.04691 | 0.34836 | 0.6098 | **H330** | 0.73929 | 0.65877 | 0.95757 | **H510** | 0.3974 | 1.01078 | 1.29008 |
| **C151** | 0.017 | 0.43198 | 0.60541 | **C331** | 0.70938 | 0.74239 | 0.95318 | **C511** | 0.36749 | 1.09441 | 1.28568 |
| **C152** | 0.06156 | 0.47095 | 0.6024 | **C332** | 0.75394 | 0.78137 | 0.95017 | **C512** | 0.41206 | 1.13338 | 1.28268 |
| **C153** | 0.01243 | 0.38791 | 0.60797 | **C333** | 0.70481 | 0.69832 | 0.95574 | **C513** | 0.36292 | 1.05034 | 1.28824 |
| **C154** | 0.1006 | 0.46571 | 0.60364 | **C334** | 0.79297 | 0.77612 | 0.95141 | **C514** | 0.45108 | 1.12813 | 1.28391 |
| **C155** | 0.05667 | 0.07542 | 0.56656 | **C335** | 0.74904 | 0.38583 | 0.91433 | **C515** | 0.40715 | 0.73784 | 1.24684 |
| **C156** | 0.10363 | 0.10791 | 0.5746 | **C336** | 0.79601 | 0.41832 | 0.92237 | **C516** | 0.45412 | 0.77033 | 1.25488 |
| **H157** | 0.03354 | 0.08853 | 0.55342 | **H337** | 0.72592 | 0.39894 | 0.9012 | **H517** | 0.38403 | 0.75095 | 1.2337 |
| **C158** | 0.12037 | 0.0463 | 0.59426 | **C338** | 0.81275 | 0.35671 | 0.94203 | **C518** | 0.47086 | 0.70872 | 1.27454 |
| **C159** | 0.13578 | 0.09351 | 0.58872 | **C339** | 0.82816 | 0.40392 | 0.93649 | **C519** | 0.48627 | 0.75593 | 1.269 |
| **H160** | 0.11603 | 0.14638 | 0.5697 | **H340** | 0.80841 | 0.45679 | 0.91747 | **H520** | 0.46652 | 0.8088 | 1.24998 |
| **C161** | 0.04102 | 0.02795 | 0.57362 | **C341** | 0.73339 | 0.33837 | 0.92139 | **C521** | 0.3915 | 0.69038 | 1.25389 |
| **C162** | 0.07339 | 0.01382 | 0.58715 | **C342** | 0.76577 | 0.32423 | 0.93492 | **C522** | 0.42388 | 0.67625 | 1.26742 |
| **H163** | 0.06239 | -0.02165 | 0.58766 | **H343** | 0.75477 | 0.28877 | 0.93543 | **H523** | 0.41288 | 0.64078 | 1.26793 |
| **H164** | 0.14457 | 0.03442 | 0.60421 | **H344** | 0.83695 | 0.34484 | 0.95198 | **H524** | 0.49506 | 0.69685 | 1.28448 |
| **C165** | 0.5596 | 0.03456 | 0.6035 | **C345** | 1.25198 | 0.34497 | 0.95127 | **C525** | 0.91009 | 0.69699 | 1.28378 |
| **C166** | 0.52985 | 0.04306 | 0.64536 | **C346** | 1.22223 | 0.35347 | 0.99313 | **C526** | 0.88034 | 0.70548 | 1.32563 |
| **C167** | 0.59105 | 0.06963 | 0.55851 | **C347** | 1.28342 | 0.38004 | 0.90628 | **C527** | 0.94153 | 0.73205 | 1.23879 |
| **C168** | 0.53246 | 0.08619 | 0.64319 | **C348** | 1.22484 | 0.3966 | 0.99096 | **C528** | 0.88295 | 0.74862 | 1.32346 |
| **H169** | 0.50426 | 0.01628 | 0.67884 | **H349** | 1.19664 | 0.32669 | 1.02661 | **H529** | 0.85475 | 0.67871 | 1.35911 |
| **C170** | 0.5932 | 0.1125 | 0.55574 | **C350** | 1.28558 | 0.42291 | 0.90351 | **C530** | 0.94369 | 0.77493 | 1.23602 |
| **H171** | 0.61524 | 0.06298 | 0.52436 | **H351** | 1.30762 | 0.37339 | 0.87213 | **H531** | 0.96573 | 0.7254 | 1.20463 |
| **C172** | 0.56431 | 0.12126 | 0.59844 | **C352** | 1.25668 | 0.43167 | 0.94621 | **C532** | 0.9148 | 0.78368 | 1.27872 |
| **H173** | 0.50865 | 0.09307 | 0.67791 | **H353** | 1.20103 | 0.40348 | 1.02567 | **H533** | 0.85914 | 0.7555 | 1.35818 |
| **H174** | 0.61754 | 0.13877 | 0.52028 | **H354** | 1.30992 | 0.44918 | 0.86805 | **H534** | 0.96803 | 0.80119 | 1.20055 |
| **H175** | 0.64299 | 0.53749 | 0.60946 | **H355** | 1.33537 | 0.8479 | 0.95723 | **H535** | 0.99348 | 1.19991 | 1.28973 |
| **H176** | 0.50369 | 0.37574 | 0.60959 | **H356** | 1.19607 | 0.68615 | 0.95736 | **H536** | 0.85418 | 1.03817 | 1.28987 |
| **H177** | 0.44317 | 0.37048 | 0.60945 | **H357** | 1.13554 | 0.68089 | 0.95722 | **H537** | 0.79366 | 1.0329 | 1.28973 |
| **H178** | 0.42382 | 0.51291 | 0.60948 | **H358** | 1.1162 | 0.82333 | 0.95725 | **H538** | 0.77431 | 1.17534 | 1.28975 |
| **H179** | 0.64256 | 0.58378 | 0.61023 | **H359** | 1.33494 | 0.89419 | 0.958 | **H539** | 0.99305 | 1.24621 | 1.2905 |
| **H180** | 0.48627 | 0.57553 | 0.60981 | **H360** | 1.17865 | 0.88595 | 0.95758 | **H540** | 0.83676 | 1.23796 | 1.29008 |
| **C46** | -0.01309 | -0.0156 | 0.56936 | **C226** | 0.67929 | 0.29481 | 0.91713 | **C406** | 0.3374 | 0.64683 | 1.24964 |
| **C88** | 0.55785 | -0.00626 | 0.60883 | **C268** | 1.25023 | 0.30415 | 0.9566 | **C448** | 0.90834 | 0.65616 | 1.2891 |
| **C106** | -0.02837 | 0.43424 | 0.60953 | **C286** | 0.66401 | 0.74465 | 0.9573 | **C466** | 0.32212 | 1.09666 | 1.2898 |
| **C151** | 1.017 | 0.43198 | 0.60541 | **C331** | 1.70938 | 0.74239 | 0.95318 | **C511** | 1.36749 | 1.09441 | 1.28568 |
| **C161** | 1.04102 | 1.02795 | 0.57362 | **C341** | 1.73339 | 1.33837 | 0.92139 | **C521** | 1.3915 | 1.69038 | 1.25389 |
| **C165** | 0.5596 | 1.03456 | 0.6035 | **C345** | 1.25198 | 1.34497 | 0.95127 | **C525** | 0.91009 | 1.69699 | 1.28378 |


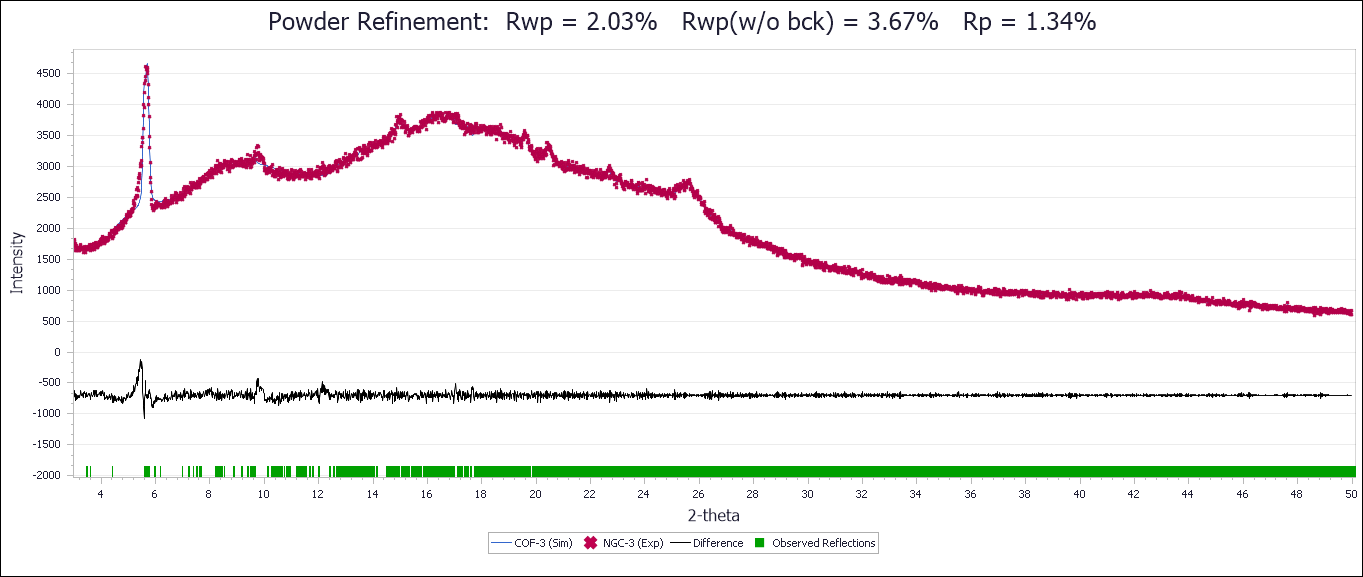


Figure S6: Pawley refinement of NGC-3. Experimental PXRD pattern after Pawley refinement is in good agreement with the simulated one.

Table S4. Fractional atomic coordinates of unit cell and lattice parameters of NGC-3.

| **NGC-3 ABC model; Space group P1**  **a = 28.48 Å, b = 28.48 Å, c = 19.84 Å; α = 89.90°, β = 91.19°, γ = 117.82°** | | | | | | | | | | | |
| --- | --- | --- | --- | --- | --- | --- | --- | --- | --- | --- | --- |
| **Atoms** | x | y | z | **Atoms** | x | y | z | **Atoms** | x | y | z |
| **C1** | 0.02841 | 0.83852 | 0.5645 | **C148** | 0.73138 | 1.21165 | 0.88562 | **C295** | 0.3458 | 1.49085 | 1.22926 |
| **C2** | 0.07615 | 0.83761 | 0.56518 | **C149** | 0.77912 | 1.21075 | 0.8863 | **C296** | 0.39353 | 1.48994 | 1.22994 |
| **C3** | 0.02983 | 0.89175 | 0.55906 | **C150** | 0.7328 | 1.26489 | 0.88018 | **C297** | 0.34722 | 1.54409 | 1.22382 |
| **C4** | 0.12941 | 0.88972 | 0.56802 | **C151** | 0.83237 | 1.26286 | 0.88914 | **C298** | 0.44679 | 1.54205 | 1.23278 |
| **C5** | 0.079 | 0.93982 | 0.55877 | **C152** | 0.78196 | 1.31296 | 0.8799 | **C299** | 0.39638 | 1.59215 | 1.22354 |
| **C6** | 0.13106 | 0.93876 | 0.56404 | **C153** | 0.83403 | 1.3119 | 0.88516 | **C300** | 0.44845 | 1.59109 | 1.2288 |
| **C7** | 0.97367 | 0.78797 | 0.56928 | **C154** | 1.67664 | 1.1611 | 0.8904 | **C301** | 1.29106 | 1.4403 | 1.23404 |
| **C8** | 0.97615 | 0.88971 | 0.55363 | **C155** | 1.67912 | 1.26285 | 0.87475 | **C302** | 1.29354 | 1.54204 | 1.21839 |
| **C9** | 0.07829 | 0.78596 | 0.56027 | **C156** | 0.78126 | 1.1591 | 0.88139 | **C303** | 0.39568 | 1.43829 | 1.22503 |
| **C10** | 0.17914 | 0.88479 | 0.5766 | **C157** | 0.8821 | 1.25793 | 0.89773 | **C304** | 0.49652 | 1.53712 | 1.24137 |
| **C11** | 0.18342 | 0.99284 | 0.56387 | **C158** | 0.88639 | 1.36598 | 0.88499 | **C305** | 0.50081 | 1.64517 | 1.22863 |
| **C12** | 0.08429 | 0.99516 | 0.55241 | **C159** | 0.78726 | 1.3683 | 0.87353 | **C306** | 0.40167 | 1.64749 | 1.21717 |
| **C13** | 0.92689 | 0.84347 | 0.54957 | **C160** | 1.62985 | 1.21661 | 0.8707 | **C307** | 1.24427 | 1.4958 | 1.21434 |
| **C14** | 0.92215 | 0.78792 | 0.55341 | **C161** | 1.62512 | 1.16106 | 0.87454 | **C308** | 1.23954 | 1.44025 | 1.21818 |
| **C15** | 0.13283 | 0.04209 | 0.55197 | **C162** | 0.83579 | 0.41523 | 0.87309 | **C309** | 0.45021 | 0.69443 | 1.21673 |
| **C16** | 0.18516 | 0.04133 | 0.55874 | **C163** | 0.88813 | 0.41447 | 0.87987 | **C310** | 0.50255 | 0.69366 | 1.22351 |
| **C17** | 0.17798 | 0.83658 | 0.57535 | **C164** | 0.88094 | 1.20972 | 0.89647 | **C311** | 0.49536 | 1.48891 | 1.24011 |
| **C18** | 0.12531 | 0.78475 | 0.56415 | **C165** | 0.82827 | 1.15789 | 0.88528 | **C312** | 0.44269 | 1.43708 | 1.22892 |
| **C19** | 0.70771 | 0.597 | 0.53425 | **C166** | 1.41068 | 0.97014 | 0.85537 | **C313** | 1.02509 | 1.24933 | 1.19901 |
| **H20** | 0.83285 | 0.77218 | 0.47588 | **H167** | 1.53582 | 1.14532 | 0.79701 | **H314** | 1.15024 | 1.42451 | 1.14065 |
| **H21** | 0.67772 | 0.60893 | 0.52325 | **H168** | 1.38069 | 0.98207 | 0.84437 | **H315** | 0.99511 | 1.26126 | 1.18801 |
| **C22** | 0.64156 | 0.49167 | 0.54652 | **C169** | 1.34453 | 0.86481 | 0.86764 | **C316** | 0.95895 | 1.144 | 1.21128 |
| **O23** | 0.60726 | 0.51227 | 0.55276 | **O170** | 1.31022 | 0.88541 | 0.87388 | **O317** | 0.92464 | 1.1646 | 1.21752 |
| **C24** | 0.39313 | 0.25128 | 0.55985 | **C171** | 1.0961 | 0.62442 | 0.88098 | **C318** | 0.71051 | 0.90361 | 1.22462 |
| **H25** | 0.38959 | 0.28606 | 0.57468 | **H172** | 1.09256 | 0.6592 | 0.8958 | **H319** | 0.70697 | 0.93839 | 1.23944 |
| **C26** | 0.23925 | 0.09455 | 0.55745 | **C173** | 0.94221 | 0.46769 | 0.87858 | **C320** | 0.55663 | 0.74689 | 1.22222 |
| **C27** | 0.28526 | 0.09848 | 0.53191 | **C174** | 0.98823 | 0.47161 | 0.85303 | **C321** | 0.60264 | 0.75081 | 1.19667 |
| **C28** | 0.24408 | 0.1439 | 0.59079 | **C175** | 0.94705 | 0.51704 | 0.91191 | **C322** | 0.56147 | 0.79623 | 1.25555 |
| **C29** | 0.33851 | 0.15047 | 0.53551 | **C176** | 1.04148 | 0.52361 | 0.85664 | **C323** | 0.65589 | 0.8028 | 1.20028 |
| **H30** | 0.28394 | 0.06409 | 0.50647 | **H177** | 0.98691 | 0.43723 | 0.82759 | **H324** | 0.60133 | 0.71642 | 1.17123 |
| **C31** | 0.2919 | 0.191 | 0.59312 | **C178** | 0.99487 | 0.56414 | 0.91424 | **C325** | 0.60929 | 0.84333 | 1.25789 |
| **H32** | 0.20872 | 0.14209 | 0.61396 | **H179** | 0.91169 | 0.51523 | 0.93508 | **H326** | 0.5261 | 0.79442 | 1.27872 |
| **C33** | 0.34245 | 0.19549 | 0.56443 | **C180** | 1.04542 | 0.56862 | 0.88555 | **C327** | 0.65984 | 0.84782 | 1.22919 |
| **H34** | 0.37349 | 0.15158 | 0.51225 | **H181** | 1.07646 | 0.52472 | 0.83337 | **H328** | 0.69088 | 0.80391 | 1.17701 |
| **H35** | 0.29362 | 0.22601 | 0.61734 | **H182** | 0.99659 | 0.59915 | 0.93846 | **H329** | 0.611 | 0.87834 | 1.2821 |
| **C36** | 0.44309 | 0.25956 | 0.5442 | **C183** | 1.14605 | 0.6327 | 0.86533 | **C330** | 0.76047 | 0.91189 | 1.20897 |
| **C37** | 0.62115 | 0.43762 | 0.53822 | **C184** | 1.32412 | 0.81076 | 0.85934 | **C331** | 0.93853 | 1.08995 | 1.20298 |
| **H38** | 0.44749 | 0.22477 | 0.53257 | **H185** | 1.15046 | 0.59791 | 0.85369 | **H332** | 0.76488 | 0.8771 | 1.19733 |
| **C39** | 0.62534 | 0.35696 | 0.50588 | **C186** | 1.3283 | 0.7301 | 0.82701 | **C333** | 0.94272 | 1.00929 | 1.17065 |
| **C40** | 0.56574 | 0.323 | 0.51612 | **C187** | 1.26871 | 0.69614 | 0.83725 | **C334** | 0.88312 | 0.97533 | 1.18089 |
| **H41** | 0.54705 | 0.27955 | 0.50916 | **H188** | 1.25002 | 0.65269 | 0.83029 | **H335** | 0.86443 | 0.93188 | 1.17393 |
| **H42** | 0.64789 | 0.33749 | 0.48985 | **H189** | 1.35085 | 0.71063 | 0.81098 | **H336** | 0.96527 | 0.98982 | 1.15462 |
| **C43** | 0.47837 | 0.31223 | 0.55245 | **C190** | 1.18134 | 0.68537 | 0.87358 | **C337** | 0.79576 | 0.96456 | 1.21722 |
| **N44** | 0.56345 | 0.40345 | 0.54632 | **N191** | 1.26641 | 0.77659 | 0.86744 | **N338** | 0.88083 | 1.05578 | 1.21108 |
| **C45** | 0.6519 | 0.41084 | 0.51653 | **C192** | 1.35487 | 0.78398 | 0.83765 | **C339** | 0.96928 | 1.06317 | 1.18129 |
| **C46** | 0.53622 | 0.34566 | 0.5357 | **C193** | 1.23919 | 0.7188 | 0.85682 | **C340** | 0.8536 | 0.99799 | 1.20046 |
| **H47** | 0.22188 | 0.99436 | 0.5685 | **H194** | 0.92485 | 1.3675 | 0.88962 | **H341** | 0.53927 | 1.64669 | 1.23326 |
| **O48** | 0.45632 | 0.33638 | 0.57469 | **O195** | 1.15929 | 0.70952 | 0.89582 | **O342** | 0.77371 | 0.98871 | 1.23946 |
| **H49** | 0.69528 | 0.43375 | 0.50808 | **H196** | 1.39825 | 0.80688 | 0.82921 | **H343** | 1.01266 | 1.08608 | 1.17285 |
| **C50** | 0.6886 | 0.54317 | 0.5429 | **C197** | 1.39157 | 0.9163 | 0.86403 | **C344** | 1.00599 | 1.1955 | 1.20767 |
| **H51** | 0.71613 | 0.52774 | 0.55053 | **H198** | 1.4191 | 0.90088 | 0.87166 | **H345** | 1.03352 | 1.18007 | 1.2153 |
| **C52** | 0.76616 | 0.64228 | 0.53453 | **C199** | 1.46912 | 1.01542 | 0.85565 | **C346** | 1.08354 | 1.29461 | 1.19929 |
| **C53** | 0.80997 | 0.6393 | 0.56301 | **C200** | 1.51294 | 1.01244 | 0.88413 | **C347** | 1.12735 | 1.29163 | 1.22777 |
| **C54** | 0.7762 | 0.69138 | 0.50508 | **C201** | 1.47917 | 1.06452 | 0.8262 | **C348** | 1.09358 | 1.34371 | 1.16984 |
| **C55** | 0.86278 | 0.68504 | 0.56607 | **C202** | 1.56574 | 1.05817 | 0.88719 | **C349** | 1.18016 | 1.33737 | 1.23083 |
| **H56** | 0.8031 | 0.60164 | 0.58506 | **H203** | 1.50606 | 0.97477 | 0.90618 | **H350** | 1.12048 | 1.25397 | 1.24982 |
| **C57** | 0.82744 | 0.73801 | 0.50554 | **C204** | 1.53041 | 1.11114 | 0.82666 | **C351** | 1.14482 | 1.39034 | 1.1703 |
| **H58** | 0.74357 | 0.69353 | 0.47989 | **H205** | 1.44654 | 1.06666 | 0.80102 | **H352** | 1.06095 | 1.34586 | 1.14466 |
| **C59** | 0.87382 | 0.73973 | 0.5424 | **C206** | 1.57679 | 1.11287 | 0.86352 | **C353** | 1.19121 | 1.39206 | 1.20716 |
| **H60** | 0.89357 | 0.68007 | 0.59137 | **H207** | 1.59653 | 1.05321 | 0.9125 | **H354** | 1.21095 | 1.3324 | 1.25614 |
| **C61** | 0.13866 | 0.58799 | 0.54206 | **C208** | 0.84163 | 0.96113 | 0.86318 | **C355** | 0.45605 | 1.24032 | 1.20682 |
| **H62** | 0.06212 | 0.70094 | 0.47277 | **H209** | 0.76508 | 1.07408 | 0.7939 | **H356** | 0.3795 | 1.35327 | 1.13754 |
| **H63** | 0.18227 | 0.60782 | 0.54859 | **H210** | 0.88524 | 0.98096 | 0.86971 | **H357** | 0.49965 | 1.26015 | 1.21336 |
| **C64** | 0.12601 | 0.4964 | 0.52 | **C211** | 0.82898 | 0.86954 | 0.84112 | **C358** | 0.44381 | 1.14784 | 1.19268 |
| **O65** | 0.17094 | 0.5151 | 0.47932 | **O212** | 0.87391 | 0.88824 | 0.80044 | **O359** | 0.49384 | 1.16785 | 1.16302 |
| **C66** | 0.14808 | 0.24918 | 0.50362 | **C213** | 0.85105 | 0.62232 | 0.82474 | **C360** | 0.46547 | 0.90151 | 1.16838 |
| **H67** | 0.18227 | 0.2693 | 0.46956 | **H214** | 0.88524 | 0.64243 | 0.79069 | **H361** | 0.49966 | 0.92163 | 1.13433 |
| **C68** | 0.13034 | 0.09271 | 0.53695 | **C215** | 0.83331 | 0.46584 | 0.85807 | **C362** | 0.44772 | 0.74504 | 1.20171 |
| **C69** | 0.17641 | 0.13416 | 0.50506 | **C216** | 0.87938 | 0.5073 | 0.82619 | **C363** | 0.4938 | 0.7865 | 1.16983 |
| **C70** | 0.08941 | 0.10348 | 0.55786 | **C217** | 0.79238 | 0.47662 | 0.87899 | **C364** | 0.4068 | 0.75581 | 1.22263 |
| **C71** | 0.1816 | 0.18515 | 0.49428 | **C218** | 0.88457 | 0.55829 | 0.8154 | **C365** | 0.49899 | 0.83748 | 1.15905 |
| **H72** | 0.20836 | 0.1269 | 0.48808 | **H219** | 0.91132 | 0.50004 | 0.8092 | **H366** | 0.52574 | 0.77923 | 1.15285 |
| **C73** | 0.09499 | 0.15503 | 0.54801 | **C220** | 0.79796 | 0.52817 | 0.86914 | **C367** | 0.41237 | 0.80736 | 1.21278 |
| **H74** | 0.05344 | 0.07214 | 0.58257 | **H221** | 0.75641 | 0.44527 | 0.9037 | **H368** | 0.37083 | 0.72447 | 1.24734 |
| **C75** | 0.14133 | 0.1966 | 0.51593 | **C222** | 0.8443 | 0.56974 | 0.83706 | **C369** | 0.45871 | 0.84893 | 1.1807 |
| **H76** | 0.21752 | 0.21597 | 0.46886 | **H223** | 0.92049 | 0.58911 | 0.78999 | **H370** | 0.5349 | 0.8683 | 1.13363 |
| **H77** | 0.06261 | 0.16175 | 0.56433 | **H224** | 0.76557 | 0.53489 | 0.88545 | **H371** | 0.37999 | 0.81408 | 1.22909 |
| **C78** | 0.12672 | 0.28007 | 0.53169 | **C225** | 0.82969 | 0.65321 | 0.85281 | **C372** | 0.44411 | 0.9324 | 1.19645 |
| **C79** | 0.10562 | 0.44506 | 0.54075 | **C226** | 0.80916 | 0.81974 | 0.85357 | **C373** | 0.42199 | 1.09883 | 1.20406 |
| **H80** | 0.09469 | 0.26078 | 0.56836 | **H227** | 0.79766 | 0.63392 | 0.88948 | **H374** | 0.41208 | 0.91311 | 1.23313 |
| **C81** | 0.03311 | 0.36424 | 0.5953 | **C228** | 0.73548 | 0.73783 | 0.90473 | **C375** | 0.34768 | 1.01432 | 1.24914 |
| **C82** | 0.06087 | 0.33349 | 0.57701 | **C229** | 0.76433 | 0.70783 | 0.88811 | **C376** | 0.37799 | 0.9859 | 1.23183 |
| **H83** | 0.04129 | 0.29085 | 0.59005 | **H230** | 0.74472 | 0.66505 | 0.90037 | **H377** | 0.35871 | 0.94272 | 1.24143 |
| **H84** | -0.00489 | 0.34356 | 0.62281 | **H231** | 0.69665 | 0.71648 | 0.93032 | **H378** | 0.3081 | 0.99139 | 1.27262 |
| **H85** | 0.97142 | 0.7504 | 0.58293 | **H232** | 1.67439 | 1.12354 | 0.90406 | **H379** | 1.28881 | 1.40273 | 1.2477 |
| **C86** | 0.13513 | 0.3279 | 0.51938 | **C233** | 0.83809 | 0.70103 | 0.8405 | **C380** | 0.45203 | 0.97901 | 1.18419 |
| **N87** | 0.13165 | 0.41412 | 0.52516 | **N234** | 0.83618 | 0.78949 | 0.83955 | **N381** | 0.45038 | 1.07004 | 1.18928 |
| **C88** | 0.05357 | 0.41661 | 0.57816 | **C235** | 0.75597 | 0.79036 | 0.88836 | **C382** | 0.36779 | 1.06732 | 1.23594 |
| **C89** | 0.10827 | 0.35775 | 0.54314 | **C236** | 0.81276 | 0.73295 | 0.85663 | **C383** | 0.42738 | 1.01298 | 1.20292 |
| **O90** | 0.16032 | 0.34519 | 0.46664 | **O237** | 0.86329 | 0.71832 | 0.78776 | **O384** | 0.4882 | 1.0024 | 1.14268 |
| **H91** | 0.03187 | 0.43721 | 0.59292 | **H238** | 0.73345 | 0.81039 | 0.90184 | **H385** | 0.34419 | 1.08613 | 1.2498 |
| **C92** | 0.11781 | 0.53771 | 0.52848 | **C239** | 0.82077 | 0.91085 | 0.8496 | **C386** | 0.43519 | 1.19004 | 1.19324 |
| **H93** | 0.0745 | 0.52156 | 0.52691 | **H240** | 0.77746 | 0.8947 | 0.84804 | **H387** | 0.39188 | 1.17389 | 1.19168 |
| **C94** | 0.12765 | 0.63381 | 0.53208 | **C241** | 0.83062 | 1.00695 | 0.8532 | **C388** | 0.44503 | 1.28614 | 1.19684 |
| **C95** | 0.16107 | 0.67298 | 0.5799 | **C242** | 0.86403 | 1.04612 | 0.90102 | **C389** | 0.47845 | 1.32531 | 1.24467 |
| **C96** | 0.09012 | 0.64525 | 0.4929 | **C243** | 0.79308 | 1.01839 | 0.81402 | **C390** | 0.4075 | 1.29758 | 1.15766 |
| **C97** | 0.159 | 0.72105 | 0.58866 | **C244** | 0.86197 | 1.09419 | 0.90979 | **C391** | 0.47638 | 1.37338 | 1.25343 |
| **H98** | 0.18942 | 0.66743 | 0.61029 | **H245** | 0.89238 | 1.04057 | 0.93141 | **H392** | 0.5068 | 1.31976 | 1.27505 |
| **C99** | 0.08942 | 0.6934 | 0.50284 | **C246** | 0.79238 | 1.06654 | 0.82397 | **C393** | 0.4068 | 1.34574 | 1.16761 |
| **H100** | 0.06313 | 0.61655 | 0.45573 | **H247** | 0.76609 | 0.98968 | 0.77686 | **H394** | 0.38051 | 1.26887 | 1.1205 |
| **C101** | 0.12391 | 0.73201 | 0.55011 | **C248** | 0.82688 | 1.10515 | 0.87123 | **C395** | 0.4413 | 1.38434 | 1.21487 |
| **H102** | 0.18529 | 0.75019 | 0.6259 | **H249** | 0.88826 | 1.12333 | 0.94702 | **H396** | 0.50268 | 1.40252 | 1.29066 |
| **H103** | 0.04881 | 0.99901 | 0.54504 | **H250** | 0.75178 | 1.37215 | 0.86616 | **H397** | 0.36619 | 1.65134 | 1.20981 |
| **C104** | 0.51448 | 0.76052 | 0.56635 | **C251** | 1.21744 | 1.13365 | 0.88747 | **C398** | 0.83186 | 1.41285 | 1.23111 |
| **H105** | 0.4399 | 0.7581 | 0.57453 | **H252** | 1.14287 | 1.13124 | 0.89566 | **H399** | 0.75728 | 1.41043 | 1.2393 |
| **C106** | 0.57475 | 0.79316 | 0.56483 | **C253** | 1.27771 | 1.1663 | 0.88595 | **C400** | 0.89213 | 1.44549 | 1.22959 |
| **H107** | 0.59726 | 0.77226 | 0.55641 | **H254** | 1.30022 | 1.1454 | 0.87753 | **H401** | 0.91464 | 1.42459 | 1.22117 |
| **H108** | 0.49506 | 0.71683 | 0.56107 | **H255** | 1.19803 | 1.08997 | 0.88219 | **H402** | 0.81244 | 1.36916 | 1.22583 |
| **C109** | 0.65561 | 0.87377 | 0.57034 | **C256** | 1.35858 | 1.24691 | 0.89146 | **C403** | 0.97299 | 1.5261 | 1.23511 |
| **C110** | 0.69332 | 0.85177 | 0.5642 | **C257** | 1.39628 | 1.22491 | 0.88532 | **C404** | 1.0107 | 1.5041 | 1.22896 |
| **H111** | 0.67894 | 0.81003 | 0.5786 | **H258** | 1.3819 | 1.18317 | 0.89972 | **H405** | 0.99632 | 1.46236 | 1.24337 |
| **C112** | 0.78881 | 0.86913 | 0.54784 | **C259** | 1.49177 | 1.24227 | 0.86897 | **C406** | 1.10619 | 1.52146 | 1.21261 |
| **C113** | 0.78644 | 0.82538 | 0.58385 | **C260** | 1.48941 | 1.19852 | 0.90497 | **C407** | 1.10383 | 1.47771 | 1.24861 |
| **C114** | 0.83569 | 0.90271 | 0.51046 | **C261** | 1.53866 | 1.27585 | 0.83158 | **C408** | 1.15308 | 1.55504 | 1.17522 |
| **C115** | 0.87917 | 0.89305 | 0.50857 | **C262** | 1.58214 | 1.26619 | 0.82969 | **C409** | 1.19655 | 1.54538 | 1.17333 |
| **H116** | 0.83886 | 0.93698 | 0.48278 | **H263** | 1.54183 | 1.31011 | 0.8039 | **H410** | 1.15624 | 1.58931 | 1.14754 |
| **C117** | 0.87758 | 0.85002 | 0.54517 | **C264** | 1.58055 | 1.22316 | 0.8663 | **C411** | 1.19497 | 1.50235 | 1.20994 |
| **C118** | 0.83073 | 0.81634 | 0.58318 | **C265** | 1.5337 | 1.18948 | 0.9043 | **C412** | 1.14812 | 1.46867 | 1.24794 |
| **H119** | 0.7514 | 0.7995 | 0.61443 | **H266** | 1.45437 | 1.17264 | 0.93556 | **H413** | 1.06879 | 1.45183 | 1.2792 |
| **H120** | 0.82858 | 0.78324 | 0.61247 | **H267** | 1.53155 | 1.15638 | 0.93359 | **H414** | 1.14597 | 1.43557 | 1.27724 |
| **C121** | 0.39294 | 0.8636 | 0.60909 | **C268** | 1.09591 | 1.23673 | 0.93021 | **C415** | 0.71033 | 1.51593 | 1.27385 |
| **H122** | 0.41676 | 0.89602 | 0.6446 | **H269** | 1.11973 | 1.26915 | 0.96573 | **H416** | 0.73415 | 1.54835 | 1.30937 |
| **C123** | 0.23441 | 0.84622 | 0.58239 | **C270** | 0.93737 | 1.21936 | 0.90351 | **C417** | 0.55179 | 1.49855 | 1.24715 |
| **C124** | 0.23742 | 0.80031 | 0.56264 | **C271** | 0.94039 | 1.17345 | 0.88376 | **C418** | 0.55481 | 1.45265 | 1.2274 |
| **C125** | 0.28141 | 0.8915 | 0.60707 | **C272** | 0.98437 | 1.26464 | 0.92819 | **C419** | 0.59879 | 1.54383 | 1.27183 |
| **C126** | 0.28687 | 0.80042 | 0.56597 | **C273** | 0.98983 | 1.17355 | 0.88709 | **C420** | 0.60425 | 1.45275 | 1.23073 |
| **H127** | 0.20197 | 0.76511 | 0.54253 | **H274** | 0.90494 | 1.13825 | 0.86365 | **H421** | 0.51936 | 1.41744 | 1.20729 |
| **C128** | 0.33054 | 0.89177 | 0.60997 | **C275** | 1.0335 | 1.2649 | 0.93109 | **C422** | 0.64792 | 1.5441 | 1.27473 |
| **H129** | 0.28033 | 0.92738 | 0.62267 | **H276** | 0.98329 | 1.30052 | 0.94379 | **H423** | 0.59771 | 1.57971 | 1.28743 |
| **C130** | 0.3343 | 0.84671 | 0.58872 | **C277** | 1.03727 | 1.21985 | 0.90985 | **C424** | 0.65169 | 1.49904 | 1.25349 |
| **H131** | 0.28821 | 0.76527 | 0.54799 | **H278** | 0.99118 | 1.13841 | 0.86912 | **H425** | 0.60559 | 1.4176 | 1.21276 |
| **H132** | 0.36624 | 0.92808 | 0.62749 | **H279** | 1.06921 | 1.30122 | 0.94861 | **H426** | 0.68363 | 1.58041 | 1.29225 |
| **C133** | 0.41799 | 0.84056 | 0.57634 | **C280** | 1.12096 | 1.2137 | 0.89746 | **C427** | 0.73537 | 1.49289 | 1.2411 |
| **C134** | 0.47824 | 0.86432 | 0.58218 | **C281** | 1.18121 | 1.23746 | 0.9033 | **C428** | 0.79562 | 1.51665 | 1.24694 |
| **H135** | 0.39498 | 0.80643 | 0.54301 | **H282** | 1.09795 | 1.17957 | 0.86413 | **H429** | 0.71237 | 1.45876 | 1.20777 |
| **C136** | 0.74548 | 0.88383 | 0.54514 | **C283** | 1.44844 | 1.25697 | 0.86626 | **C430** | 1.06286 | 1.53616 | 1.2099 |
| **H137** | 0.91448 | 0.91956 | 0.47868 | **H284** | 1.61744 | 1.2927 | 0.7998 | **H431** | 1.23186 | 1.57189 | 1.14344 |
| **H138** | 0.75775 | 0.92431 | 0.52674 | **H285** | 1.46072 | 1.29745 | 0.84786 | **H432** | 1.07514 | 1.57664 | 1.1915 |
| **O139** | 0.50778 | 0.91569 | 0.60844 | **O286** | 1.21075 | 1.28883 | 0.92956 | **O433** | 0.82516 | 1.56803 | 1.27321 |
| **O140** | 0.67407 | 0.92273 | 0.56652 | **O287** | 1.37704 | 1.29586 | 0.88764 | **O434** | 0.99145 | 1.57506 | 1.23128 |
| **C141** | 0.51004 | 0.84343 | 0.58095 | **C288** | 1.213 | 1.21657 | 0.90207 | **C435** | 0.82742 | 1.49576 | 1.24571 |
| **N142** | 0.56842 | 0.87339 | 0.58046 | **N289** | 1.27139 | 1.24653 | 0.90158 | **N436** | 0.88581 | 1.52572 | 1.24522 |
| **C143** | 0.4836 | 0.78371 | 0.57347 | **C290** | 1.18657 | 1.15685 | 0.89459 | **C437** | 0.80099 | 1.43605 | 1.23823 |
| **C144** | 0.60044 | 0.8475 | 0.57155 | **C291** | 1.3034 | 1.22064 | 0.89267 | **C438** | 0.91782 | 1.49983 | 1.23631 |
| **H145** | 0.21808 | 0.92054 | 0.58502 | **H292** | 0.92104 | 1.29368 | 0.90615 | **H439** | 0.53546 | 1.57287 | 1.24979 |
| **H146** | 0.0415 | 0.74801 | 0.55059 | **H293** | 0.74446 | 1.12115 | 0.87171 | **H440** | 0.35888 | 1.40034 | 1.21536 |
| **H147** | 0.97564 | 0.92732 | 0.55501 | **H294** | 1.67861 | 1.30046 | 0.87613 | **H441** | 1.29303 | 1.57965 | 1.21977 |
| **C1** | 1.02841 | 0.83852 | 0.5645 | **C148** | 1.73138 | 1.21165 | 0.88562 | **C295** | 1.3458 | 1.49085 | 1.22926 |
| **C3** | 1.02983 | 0.89175 | 0.55906 | **C150** | 1.7328 | 1.26489 | 0.88018 | **C297** | 1.34722 | 1.54409 | 1.22382 |
| **C7** | -0.02633 | 0.78797 | 0.56928 | **C154** | 0.67664 | 1.1611 | 0.8904 | **C301** | 0.29106 | 1.4403 | 1.23404 |
| **C8** | -0.02385 | 0.88971 | 0.55363 | **C155** | 0.67912 | 1.26285 | 0.87475 | **C302** | 0.29354 | 1.54204 | 1.21839 |
| **C11** | 0.18342 | -0.00716 | 0.56387 | **C158** | 0.88639 | 0.36598 | 0.88499 | **C305** | 0.50081 | 0.64517 | 1.22863 |
| **C12** | 0.08429 | -0.00484 | 0.55241 | **C159** | 0.78726 | 0.3683 | 0.87353 | **C306** | 0.40167 | 0.64749 | 1.21717 |
| **C15** | 0.13283 | 1.04209 | 0.55197 | **C162** | 0.83579 | 1.41523 | 0.87309 | **C309** | 0.45021 | 1.69443 | 1.21673 |
| **C16** | 0.18516 | 1.04133 | 0.55874 | **C163** | 0.88813 | 1.41447 | 0.87987 | **C310** | 0.50255 | 1.69366 | 1.22351 |

**Figure S7.** Comparison of the experimental PXRD pattern of the monomers 2,3,6,7,10,11-hexakis(4-formylphenyl) triphenylene (**HFPTP**) and 1,4-diacetylbenzene (**DB**), and **NGC-1**.

**Figure S8.** Comparison of the experimental PXRD pattern of monomers 2,3,6,7,10,11-hexakis(4-formylphenyl) triphenylene (**HFPTP**) and 4,4'-diacetylbiphenyl (**DBP**), and **NGC-2**.

**Figure S9.** Comparison of the experimental PXRD pattern of monomers 2,3,6,7,10,11-hexakis(4-formylphenyl) triphenylene (**HFPTP**) and 2,6-diacetylpyridine (**DP**), and **NGC-3**.

**Figure S10.** FT-IR spectra of **NGC-1** and its constituent monomers.

**Figure S11.** FT-IR spectra of **NGC-2** and its constituent monomers.

**Figure S12.** FT-IR spectra of **NGC-3** and its constituent monomers.

**Figure S13.** ^13^C CP-MAS NMR spectrum of a) **NGC-1**, b) **NGC-2**.


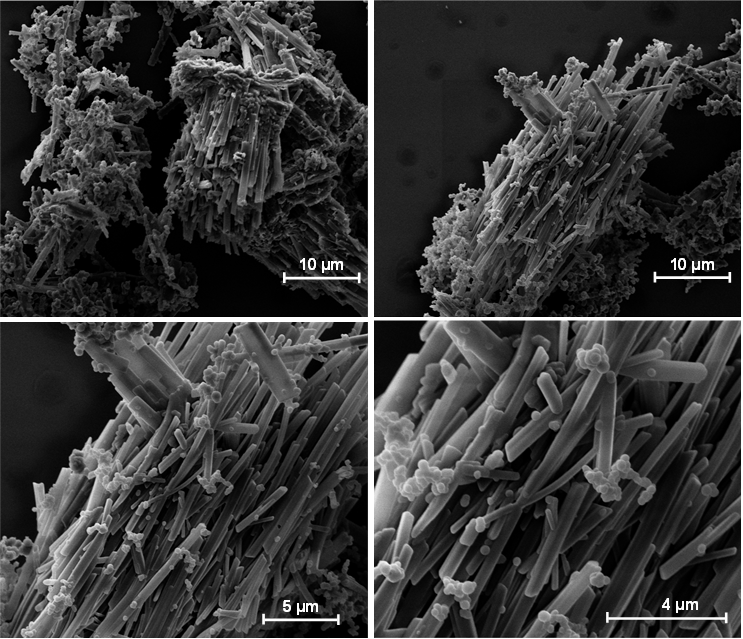


**Figure S14.** SEM images of **NGC-1**.


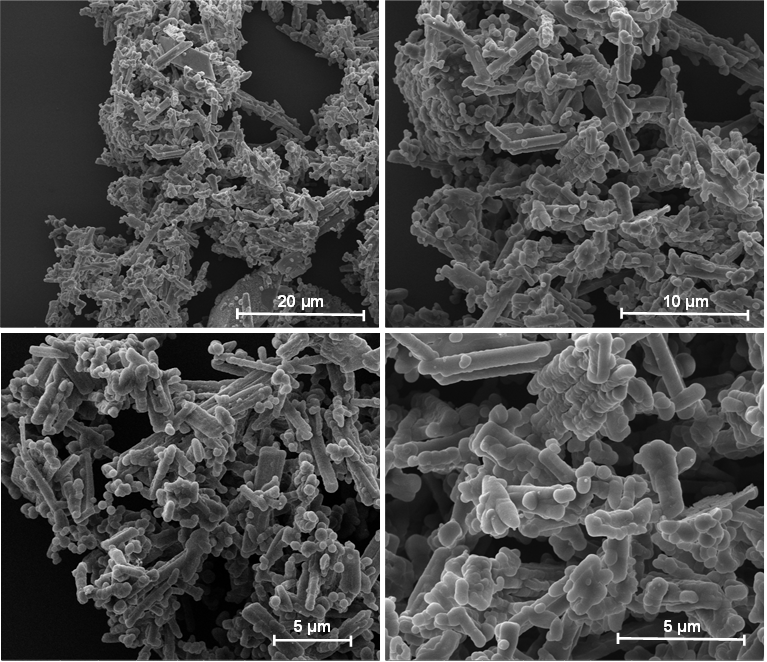


**Figure S15.** SEM images of **NGC-2**.


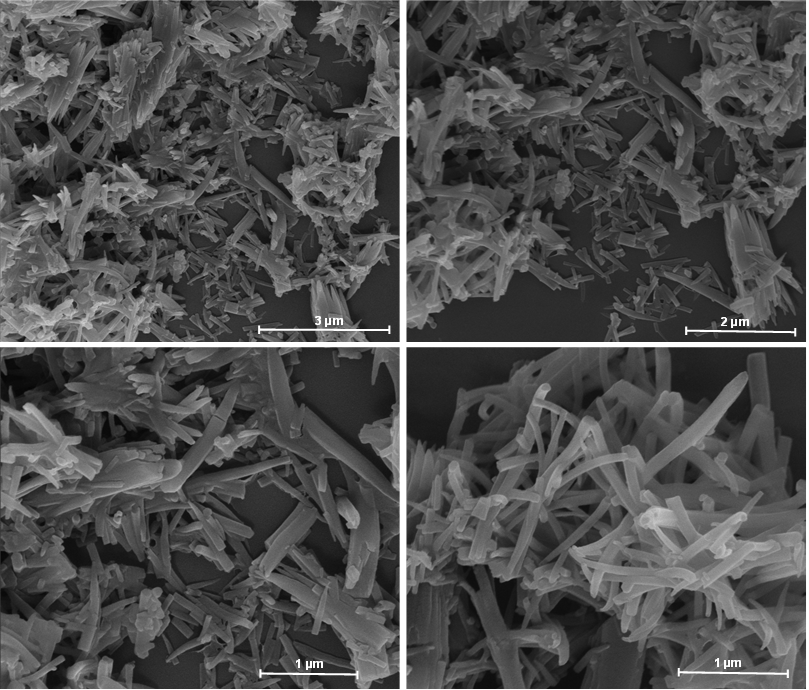


**Figure S16.** SEM images of **NGC-3**.


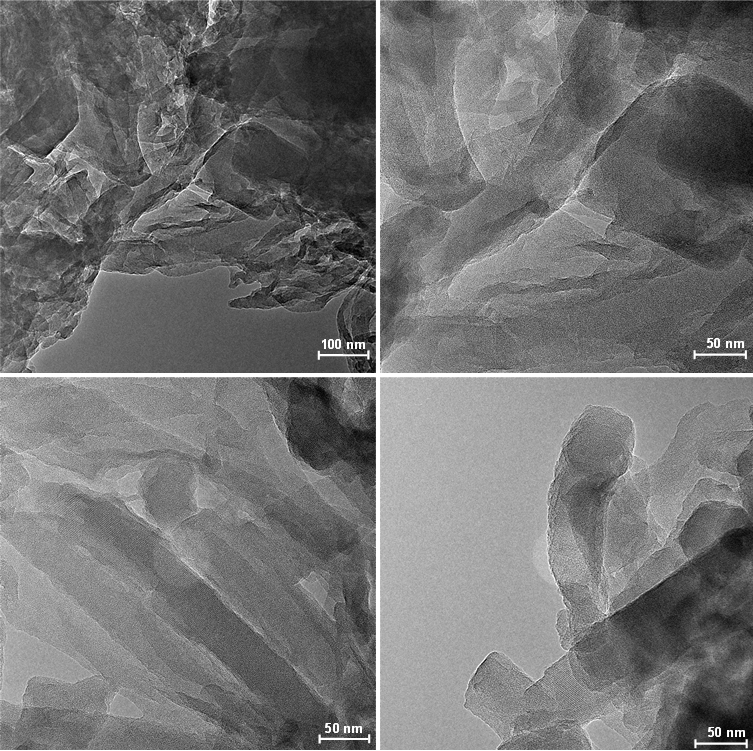


**Figure S17:** TEM images of **NGC-1**.


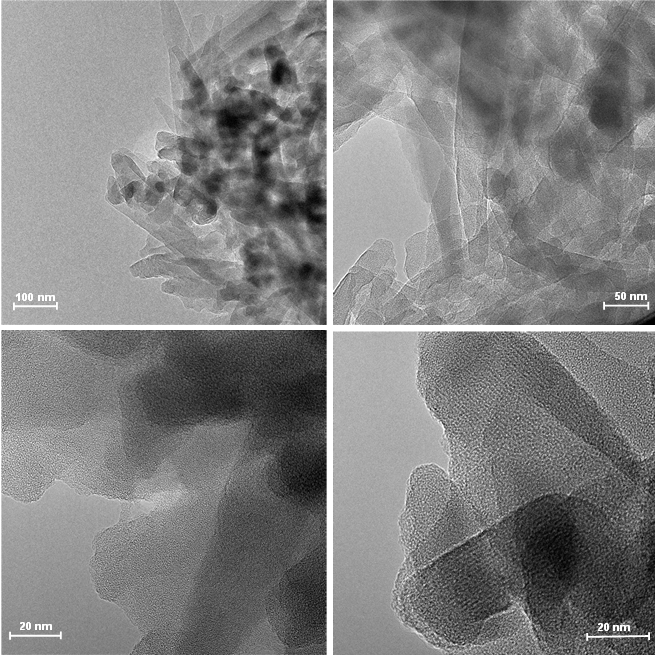


**Figure S18:** TEM images of **NGC-2**.


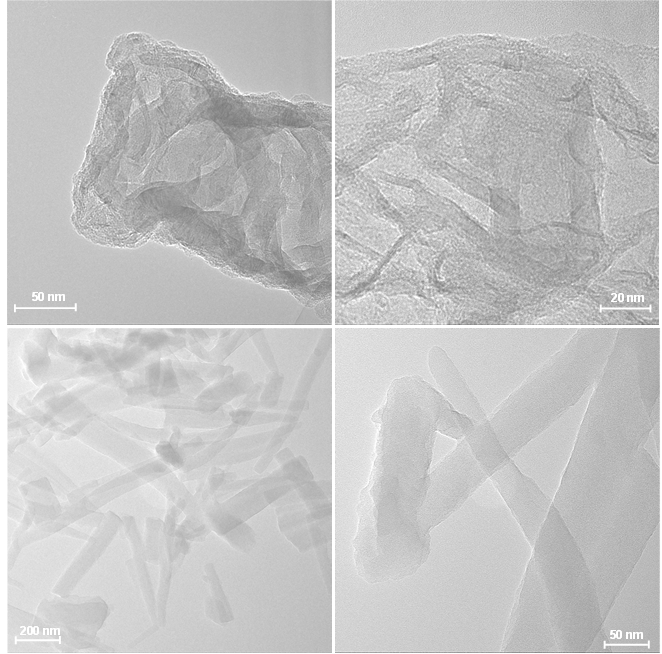


**Figure S19:** TEM images of **NGC-3**.


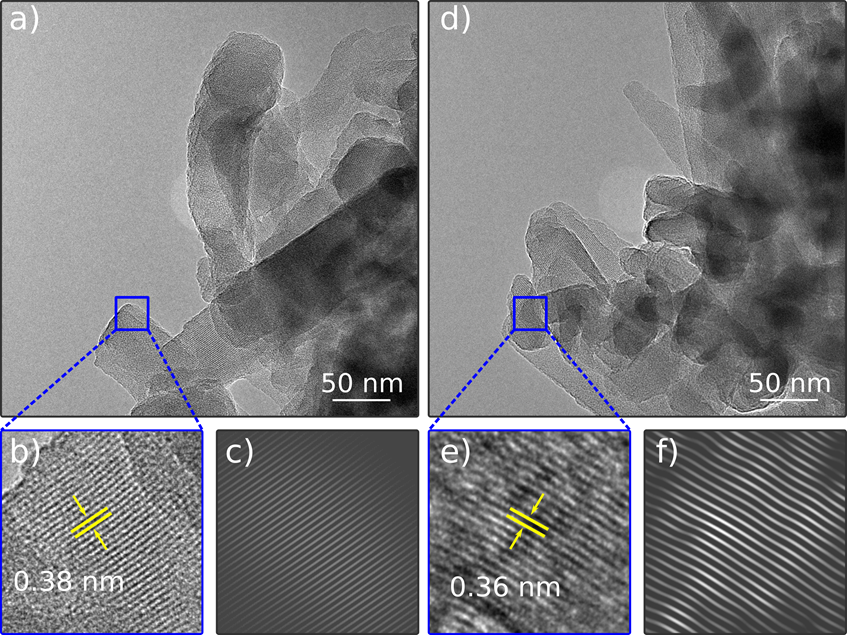


**Figure S20:** TEM images of **NGC-1** (a-c) and **NGC-2** (d-f), showing layered sheets and lattice fringes


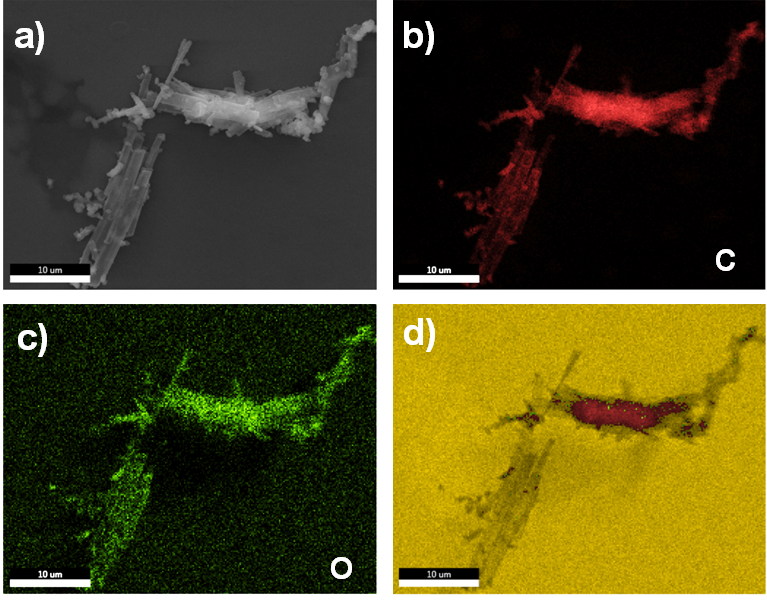


**Figure S21.** SEM-EDS elemental dot mapping of **NGC-1**. a) Reference image of the mapped material, b) carbon, c) oxygen, and d) elemental overlay.


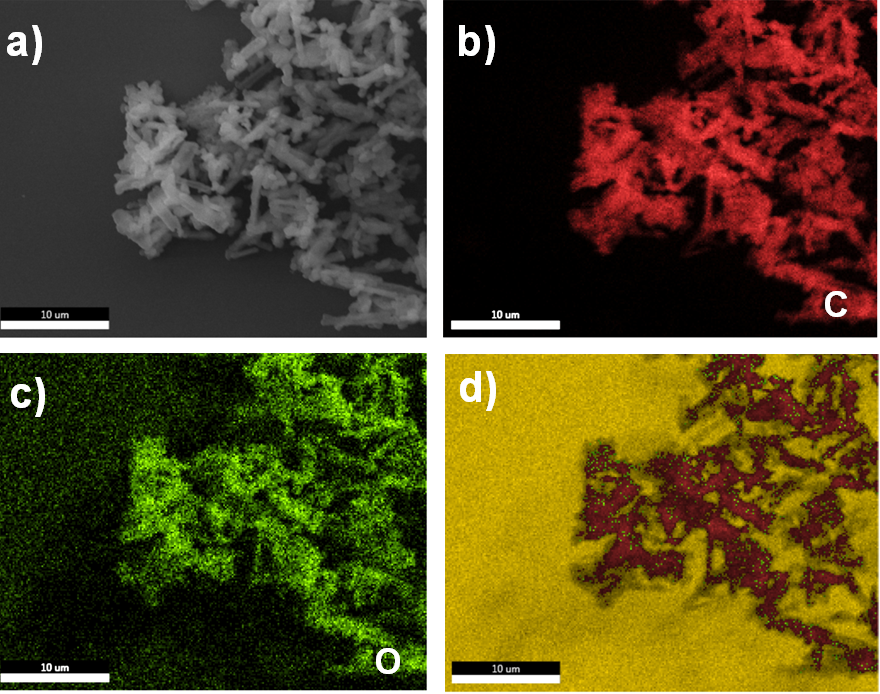


**Figure S22.** SEM-EDS elemental dot mapping of **NGC-2.** a) Reference image of the mapped material, b) carbon, c) oxygen, and d) elemental overlay.


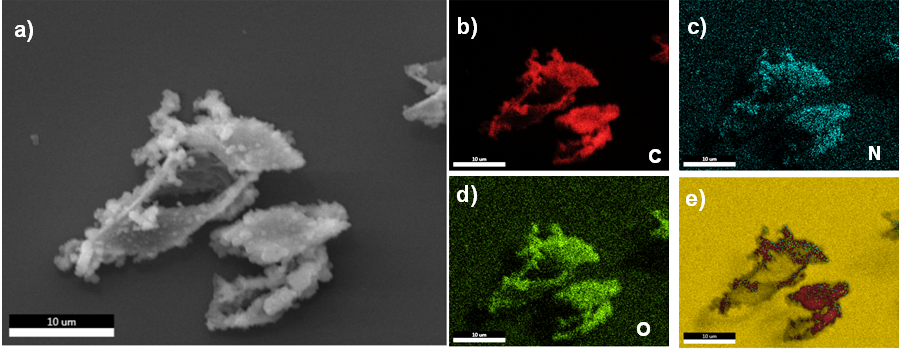


**Figure S23.** SEM-EDS elemental dot mapping of **NGC-3**. a) Reference image of the mapped material, b) carbon, c) nitrogen, d) oxygen, and e) elemental overlay.


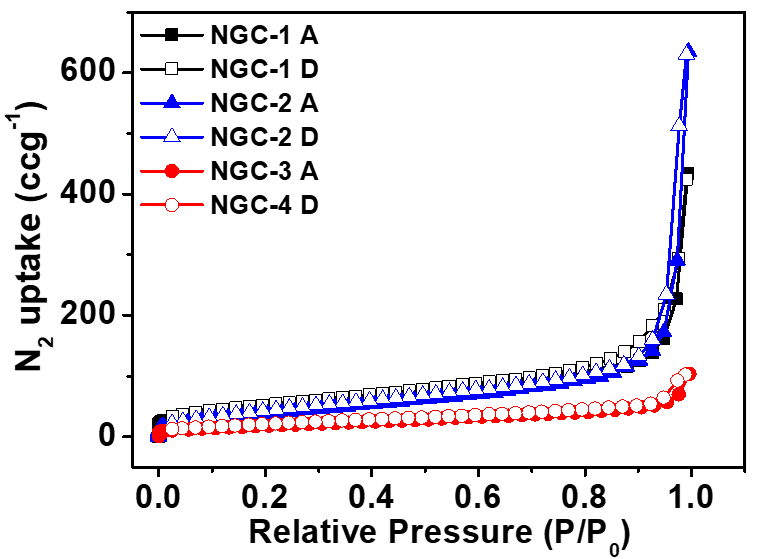


**Figure S24.** N_2_ sorption isotherm of **NGC-1-3** measured at 77 K. Filled symbols represent adsorption and empty symbols correspond to desorption.

**Figure S25.** Pore-size distribution profile of a) **NGC-1**, b) **NGC-2**, and **NGC-3**.

**Figure S26.** N_2_ sorption isotherm of a) **NGC-1** b) **NGC-2** c) **NGC-3** measured at 77 K. Filled symbols represent adsorption, and empty symbols correspond to desorption. Pore-size distribution profile of d) **NGC-1**, e) **NGC-2**, and f) **NGC-3**.

**Table S5.** Surface Area and Pore Size Distribution of Pristine NGC Samples and Their Sulfur-Loaded Counterparts

| **S. No.** | **Sample Identification** | ***Surface Area*** | **Major Pore Size Distribution (Centered on)** |
| --- | --- | --- | --- |
| 1. | **NGC-1** | 172 m^2^ g^−1^ | 1.4, 2.8, 4.6 nm |
| 2. | **NGC-2** | 150 m^2^ g^−1^ | 1.7, 2.9, 4.7 nm |
| 3. | **NGC-3** | 109 m^2^ g^−1^ | 1.4, 2.7, 4.4 nm |
| 4. | **NGC-1/S** | 61 m^2^ g^−1^ | 2.8, 4.5 nm |
| 5. | **NGC-2/S** | 29 m^2^ g^−1^ | 2.9, 4.7 nm |
| 6. | **NGC-3/S** | 26 m^2^ g^−1^ | 2.7, 4.4 nm |

**Figure S27.** Thermogravimetric analysis curves of **NGC-1,2,3**.

**Figure S28.** a) XPS survey spectra of **NGC-1** and the corresponding deconvoluted XPS spectra of b) C 1s and c) O 1s. d) XPS survey spectra of **NGC-2** and the corresponding deconvoluted XPS spectra of e) C 1s and f) O 1s.

**Figure S29.** a) XPS survey spectra of **NGC-3** and the corresponding deconvoluted XPS spectra of b) C 1s c) O1s and d) N1s.

**Figure S30.** a) Solid-state diffuse reflectance UV-visible absorption spectra of **NGC-1,2,3**. The inset shows a visible light photograph of **NGC-1**,**2**,**3**. b) Tauc plot of **NGC-1**,**2**,**3** for bandgap calculations.


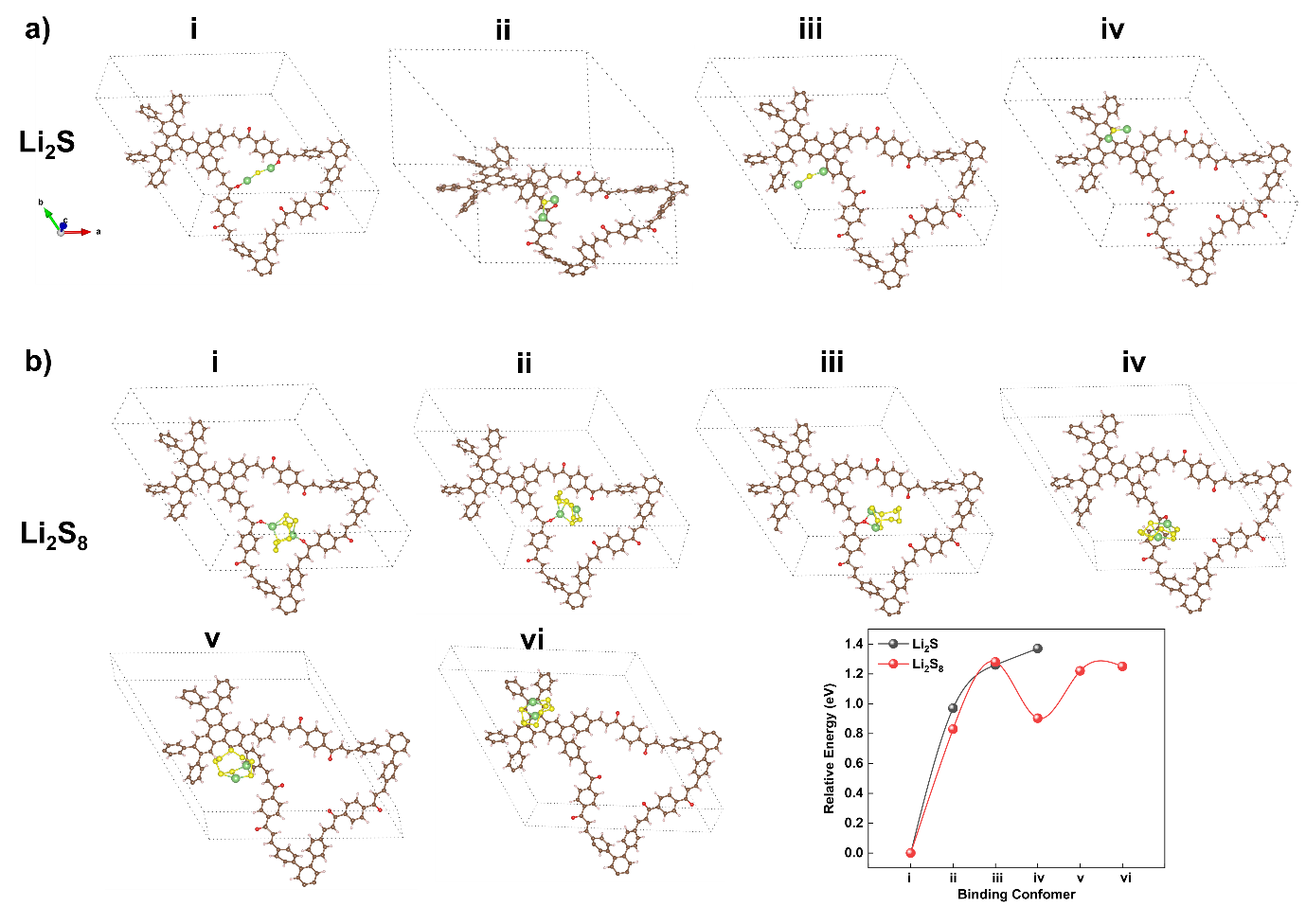


**Figure S31**: Possible binding conformers of Li_2_S and Li_2_S_8_ on **NGC-1** and their relative energies.


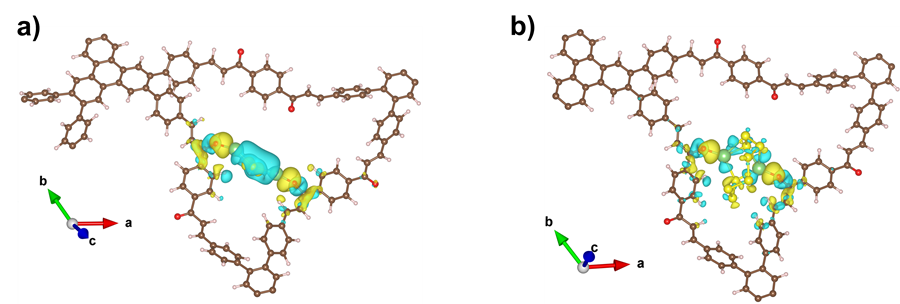


**Figure S32**: Charge density difference of Li_2_S and Li_2_S_8_ adsorbed **NGC-1** (isosurface value = 0.001 e Å^−3^)


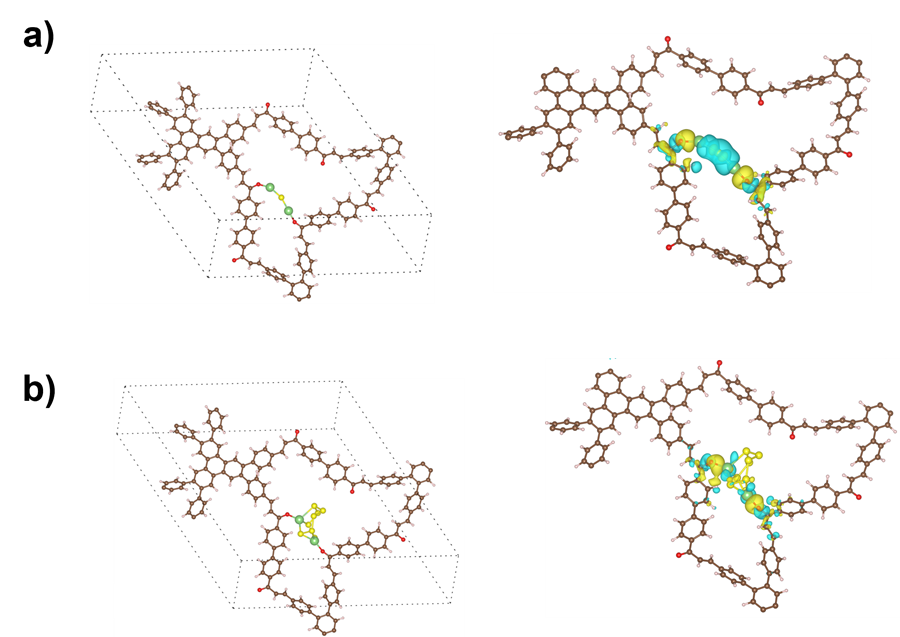


**Figure S33**: Binding configurations and corresponding charge density difference of Li_2_S and Li_2_S_8_ adsorbed **NGC-2** (isosurface value = 0.001 e Å^−3^).


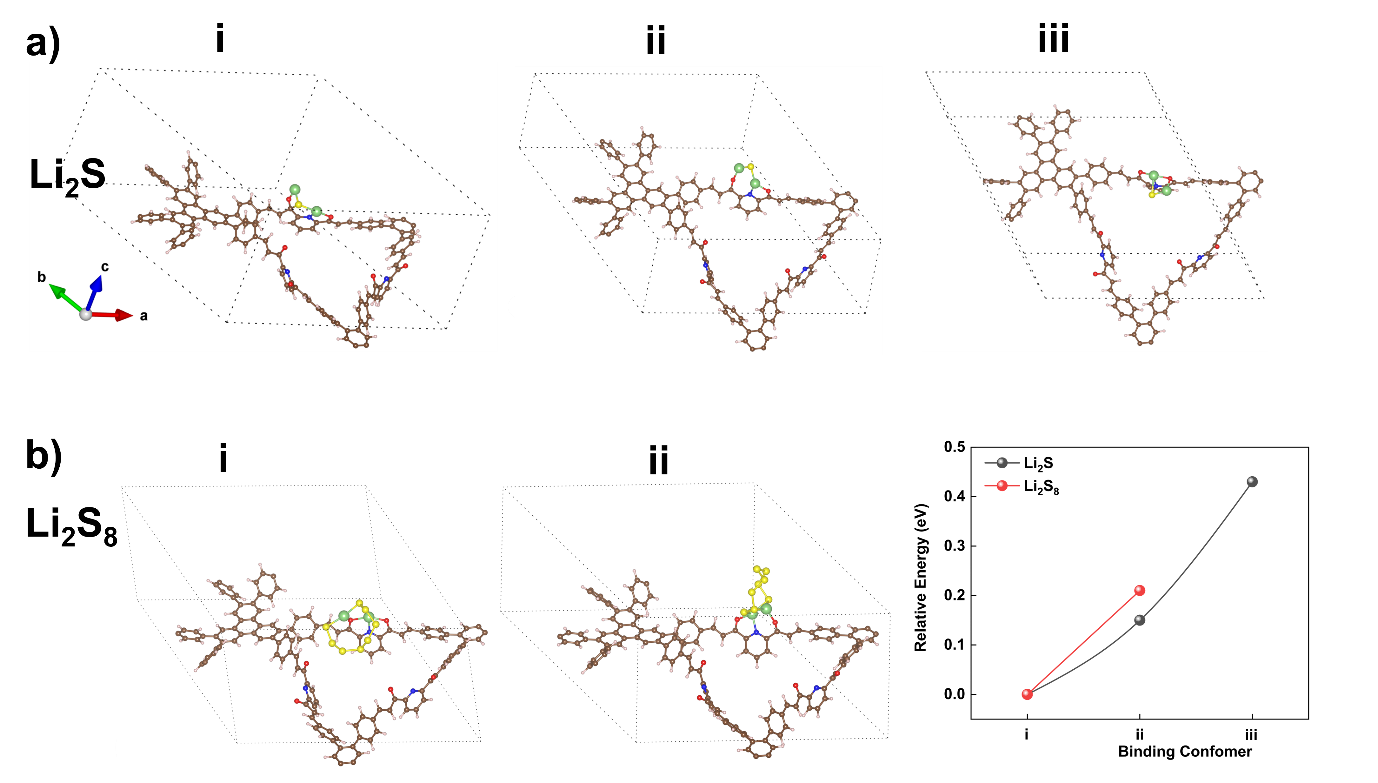


**Figure S34**: Possible binding conformers of Li_2_S and Li_2_S_8_ on the most active site of **NGC-3** and their relative energies.


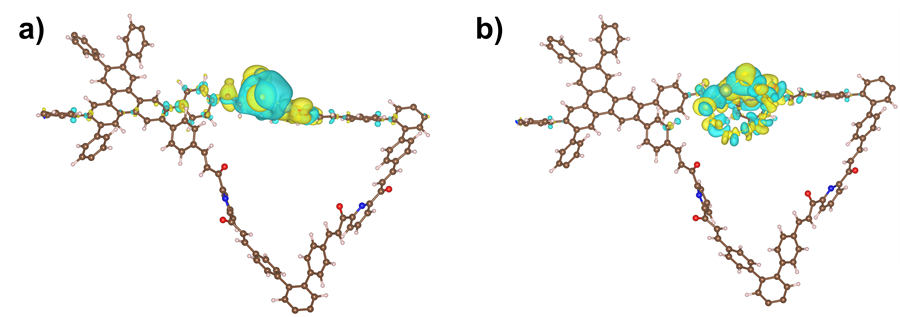


**Figure S35**: Charge density difference of Li_2_S and Li_2_S_8_ adsorbed **NGC-3** (isosurface value = 0.001 e Å^−3^).

**Table S6**: Comparison of LPS binding energies, Li-S bond lengths before (LPS molecule) and after binding with **NGC**s, and charged transfer values to **NGC**s.

| COF | Binding energy (eV) | | Li-S Bond lengths (Å) | | | | Charge transferred (\|e\|) | |
| --- | --- | --- | --- | --- | --- | --- | --- | --- |
|  | Li_2_S | Li_2_S_8_ | Li_2_S molecule | Li_2_S_8_-molecule | Li_2_S | Li_2_S_8_ | Li_2_S | Li_2_S_8_ |
| **NGC-1** | -2.44 | -2.01 | 2.08 | 2.41 | 2.20 | 2.56 | 0.44 | 0.10 |
| **NGC-2** | -2.34 | -1.74 | 2.08 | 2.41 | 2.20 | 2.55 | 0.44 | 0.07 |
| **NGC-3** | -3.42 | -2.71 | 2.08 | 2.41 | 2.29 | 2.60 | 0.84 | 0.34 |

Binding energy is calculated using the following equation,

$E_{B.E.}=E_{COF-{Li}_{2}S_{x}}-E_{COF}-E_{{Li}_{2}S_{x}}$ (1)

where, $E_{COF-{Li}_{2}S_{x}}$, $E_{COF}$, and $E_{{Li}_{2}S_{x}}$ represent the total energy for the Li_2_S_x_ adsorbed COF, pristine COF, and Li_2_S_x_ species, respectively.

The charge density difference$\Delta\rho$ is be defined as

$\Delta\rho=\rho_{COF-{Li}_{2}S_{x}}-\rho_{COF}-\rho_{{Li}_{2}S_{x}}$ (2)

where, $\rho_{COF-{Li}_{2}S_{x}}$, $\rho_{COF}$, and $\rho_{{Li}_{2}S_{x}}$ represent the charge density for the adsorption system of Li_2_S_x_ species with the modelled COF, COF, and Li_2_S_x_ species, respectively.

Moreover, DFT calculations are conducted to comprehensively explore the viability of charging and discharging sulfur species, as well as to analyze variations in **NGC-1** and **NGC-3**, each possessing distinct redox-active groups. Figure S35 shows the relative Gibbs free energy landscape for the evolution profiles from S_8_ stepwise lithiation to Li_2_S. The overall reduction reaction involving the reversible formation of Li_2_S from S_8_ and Li is considered as follows and the corresponding optimized structures are shown in Figure S34 and Figure S37:

$S_{8}^{*}+2{(Li}^{+}+e^{-})\to\mathrm{Li}_{2}S_{8}^{*}$ (3)

$\mathrm{Li}_{2}S_{8}^{*}\to\mathrm{Li}_{2}S_{6}^{*}+\frac{1}{4}S_{8}^{*}$ (4)

$\mathrm{Li}_{2}S_{6}^{*}\to\mathrm{Li}_{2}S_{4}^{*}+\frac{1}{4}S_{8}^{*}$ (5)

$\mathrm{Li}_{2}S_{4}^{*}\to\mathrm{Li}_{2}S_{2}^{*}+\frac{1}{4}S_{8}^{*}$ (6)

$\mathrm{Li}_{2}S_{2}^{*}\to\mathrm{Li}_{2}S^{*}+\frac{1}{8}S_{8}^{*}$ (7)


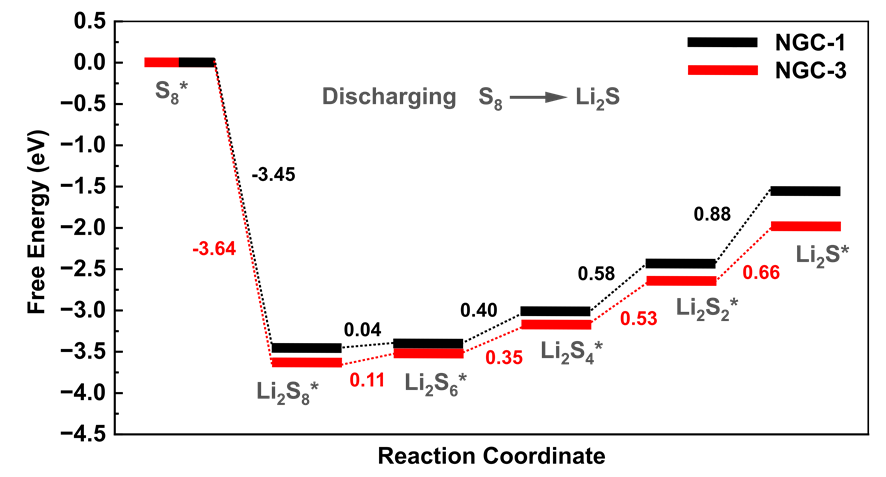


**Figure S36**: Gibbs free energy profiles for the discharging process.

For both cases, the shift from S_8_ to Li_2_S_8_ marks a spontaneous and exothermic conversion in the initial stage, while subsequent lithiation steps become endothermic. The rate-limiting step in the total discharge process is Li_2_S_2_@**NGC** $\to$ Li_2_S@**NGC** conversion with the highest positive Gibbs free energy of 0.88 eV and 0.66 eV for **NGC-1** and **NGC-3**, respectively. The lower Gibbs free energy on **NGC-3** indicates that the S reduction is more thermodynamically favorable. This feasibility for sulfur reduction within the **NGC-3** cathode host, coupled with the identified rate-limiting step, closely aligns with findings in previously examined MOF systems.^9,10^


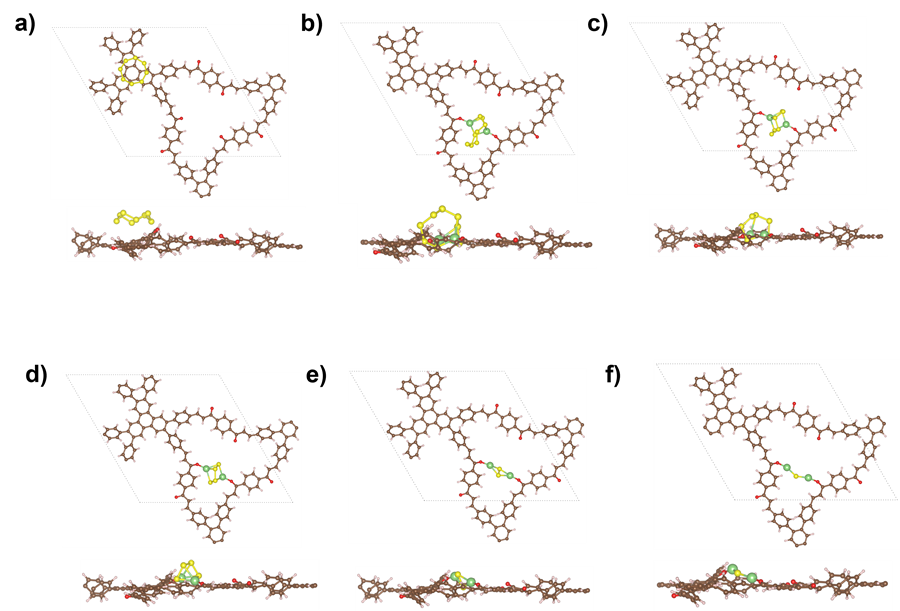


**Figure S37:** The optimized adsorption configurations of S_8_ and Li polysulfides species on the **NGC-1** surface.


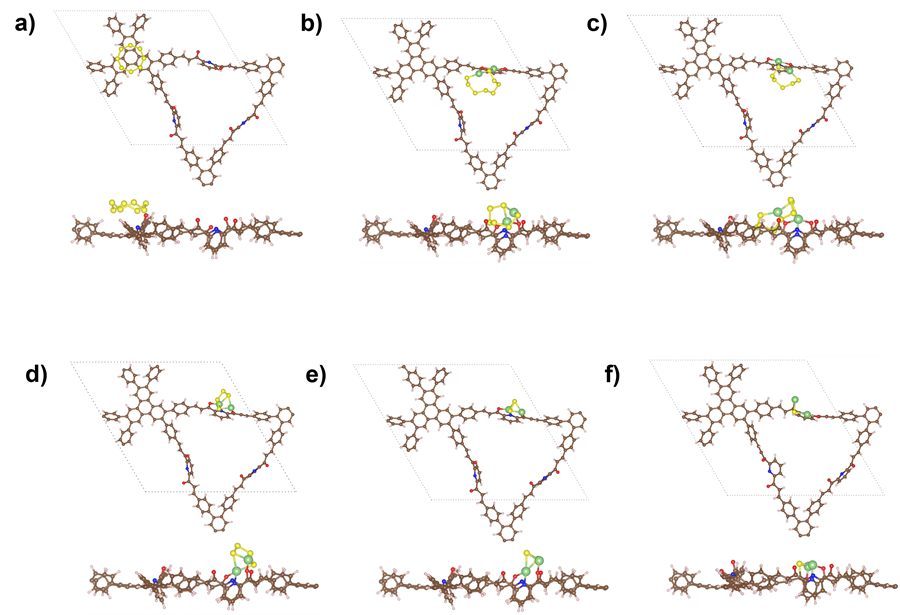


**Figure S38**: The optimized adsorption configurations of S_8_ and Li polysulfides species on the **NGC-3** surface.


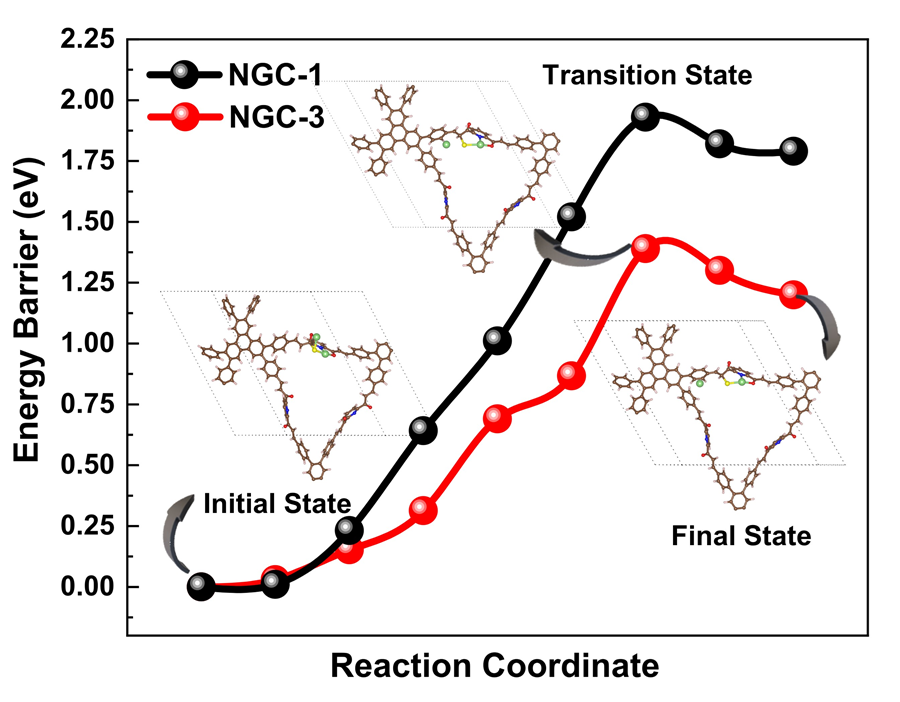


**Figure S39**: Decomposition energy barriers for the charging process on **NGC-1** and **NGC-3**.

In comparison to cathode hosts studied earlier, the barriers of **NGC-1** and **NGC-3** are either comparable to or lower than those of N-doped graphene^11^ (2.29 eV), single atomic Co N-doped graphene^12^ (1.76 eV), and 2D conductive MOFs^10^ based on triphenylene.


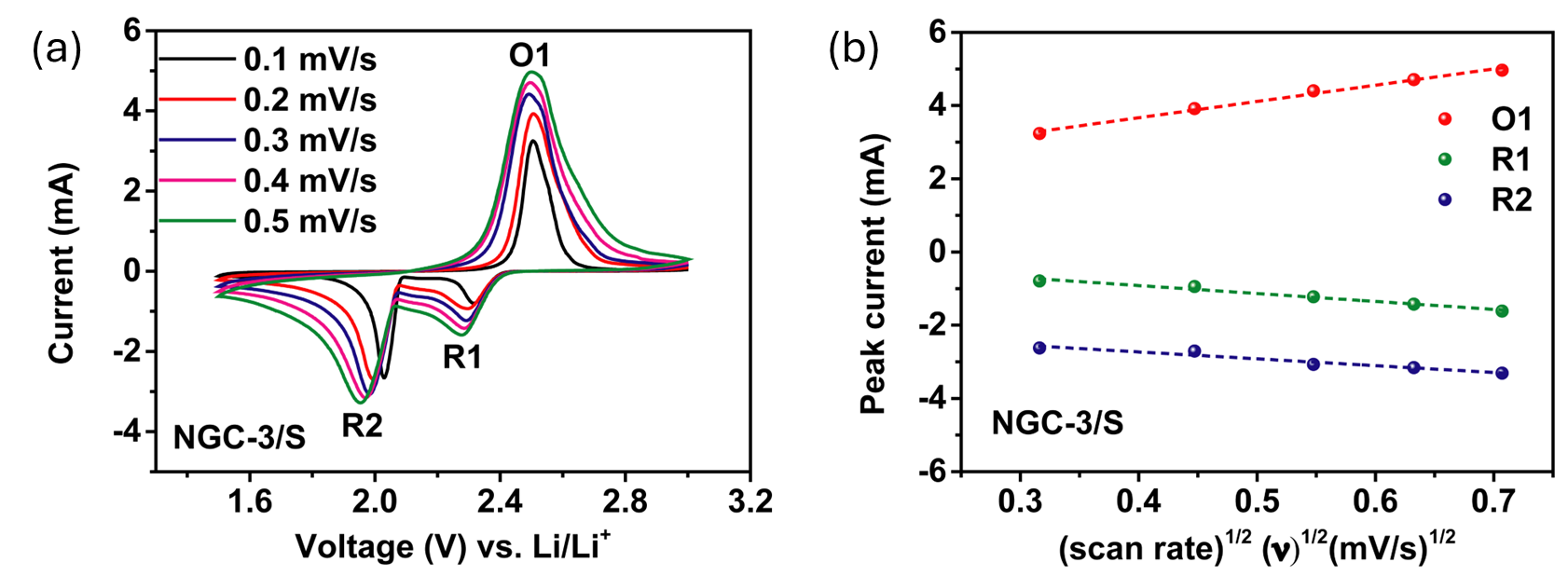


**Figure S40**: (a) CV profile of **NGC-3/S** at different scan rates (b) linear fit of oxidation (O1) and reduction (R1 and R2) peak currents with the square root of scan rates.

The CVs of **NGC-3/S** were recorded at different scan rates from 0.1 to 0.5 mV/s to understand the ion diffusion kinetics. The oxidation and reduction peak currents were plotted against the (scan rate)^0.5^. The peak currents of O1, R1 and R2 were found to vary linearly with increasing scan rates which is in accordance with the Randles Sevcik equation.

$Ip=2.69x{10}^{5}n^3/2 AC D^{0.5}\nu^{0.5}$

Ip= Peak current

n= no. of electrons involved in the redox process.

A= Area of the electrode in cm^2^

C= Concentration of Li^+^ ions in mol/dm^3^

D= Diffusion co-efficient cm^2^/s

ν= Scan rate in mV/s

*
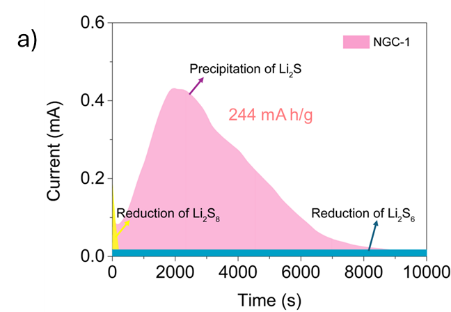

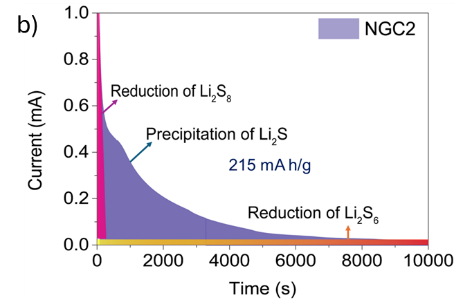

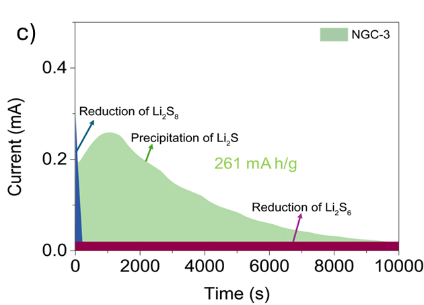
*

**Figure S41**: Current vs. time plot representing Li_2_S_8_ reduction to Li_2_S on a) **NGC-1**, b) **NGC-2**, and c) **NGC-3** with the corresponding Li_2_S precipitation capacities.


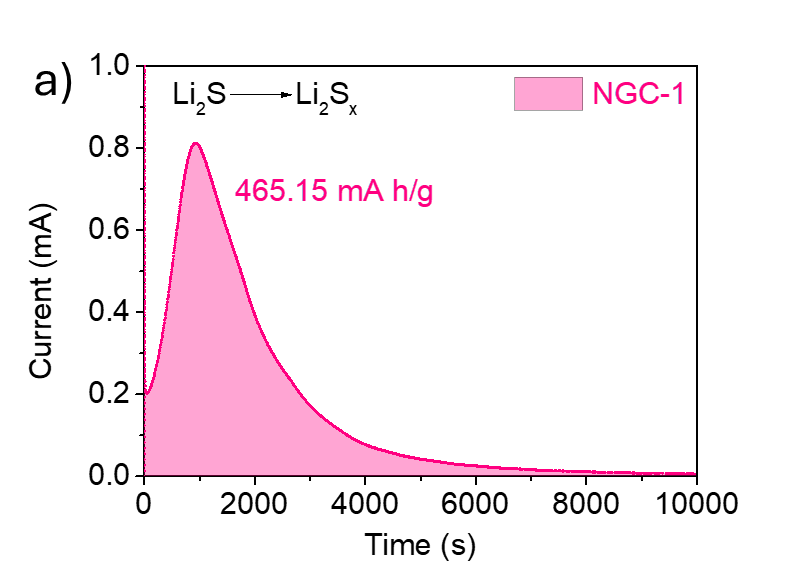

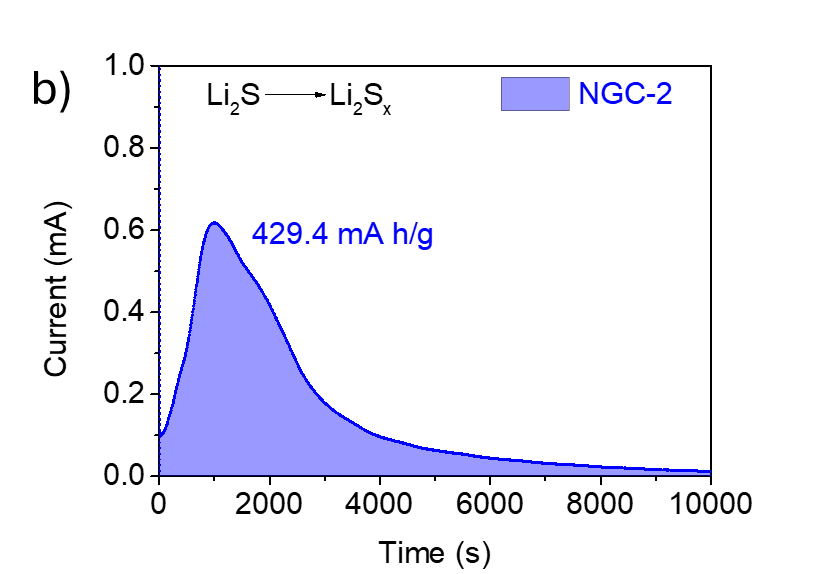

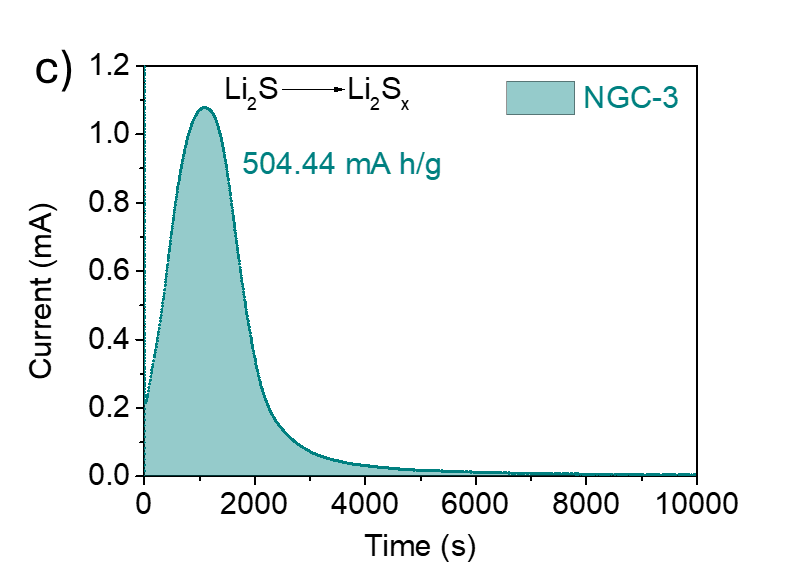


**Figure S42**: Current vs. time plot representing Li_2_S dissolution to Li_2_S_x_ on a) **NGC-1**, b) **NGC-2**, and c) **NGC-3** with the corresponding Li_2_S dissolution capacities.


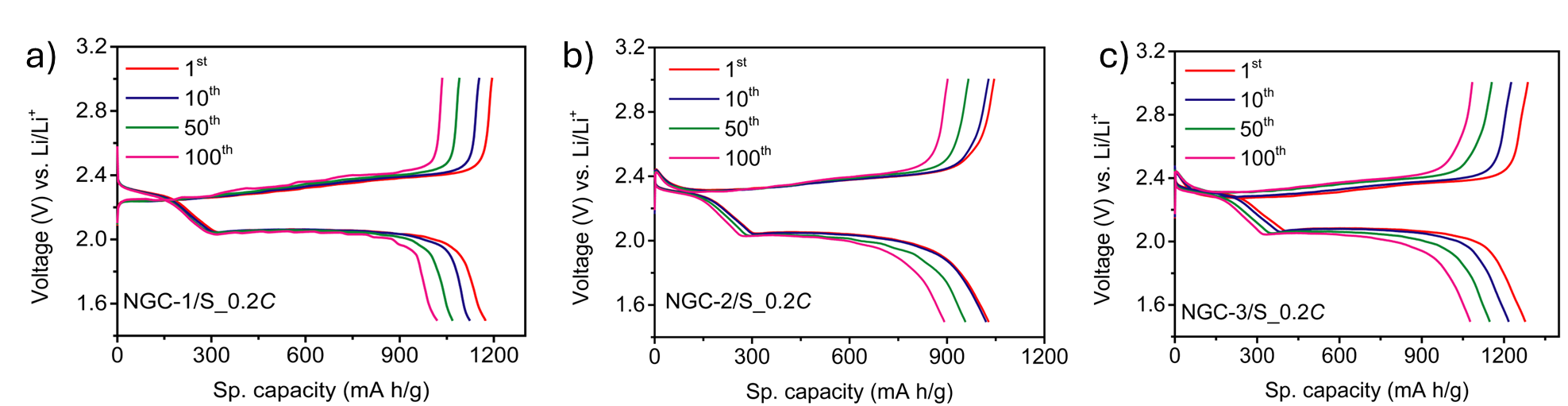


**Figure S43**: Charge-discharge profiles of a) **NGC-1/S,** b**) NGC-2/S,** and c) **NGC-3/S** at 0.2 *C*.


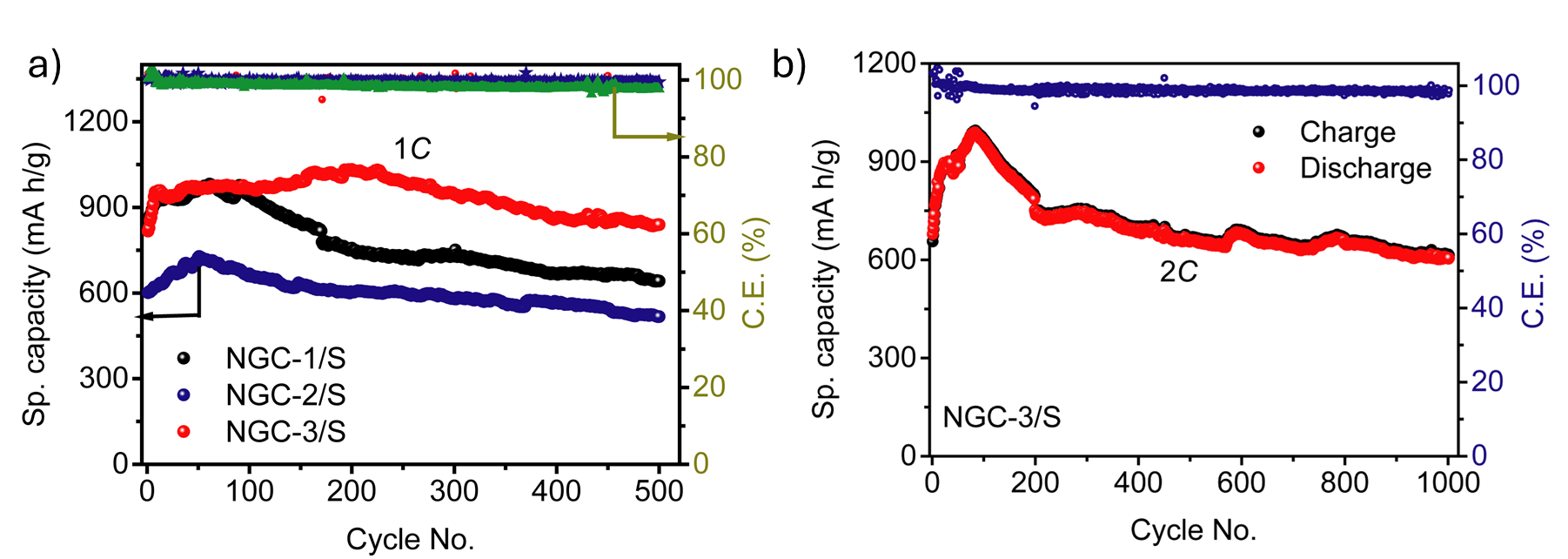


**Figure S44**: Long-term cycling profile of a) **NGC**s at 1C and **NGC-3/S** at 2*C*.


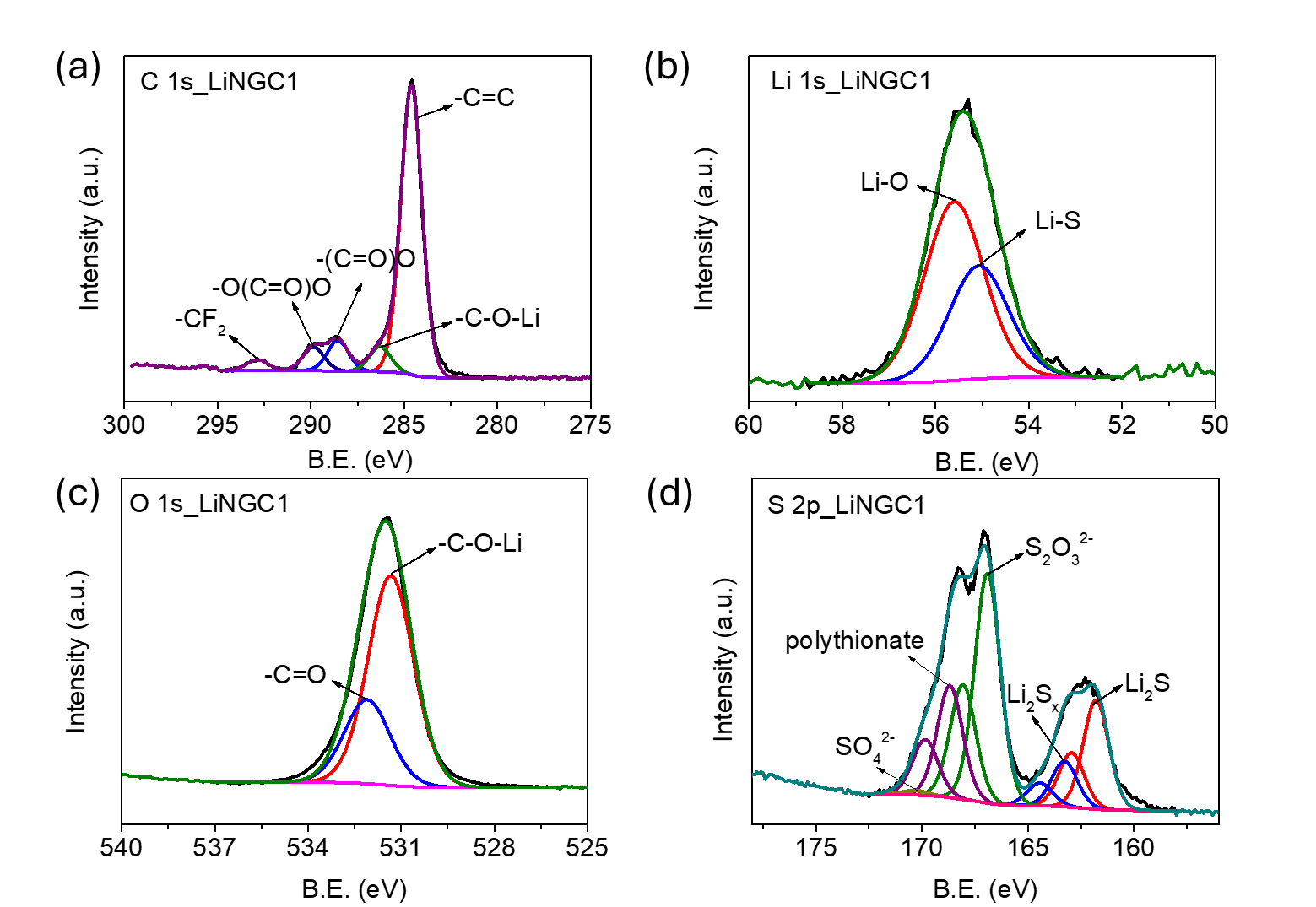


**Figure S45**: Post-cycling deconvoluted XPS spectra of **NGC-1/S** electrodes: a) C 1s, b) Li 1s, c) O 1s, and d) S 2p.


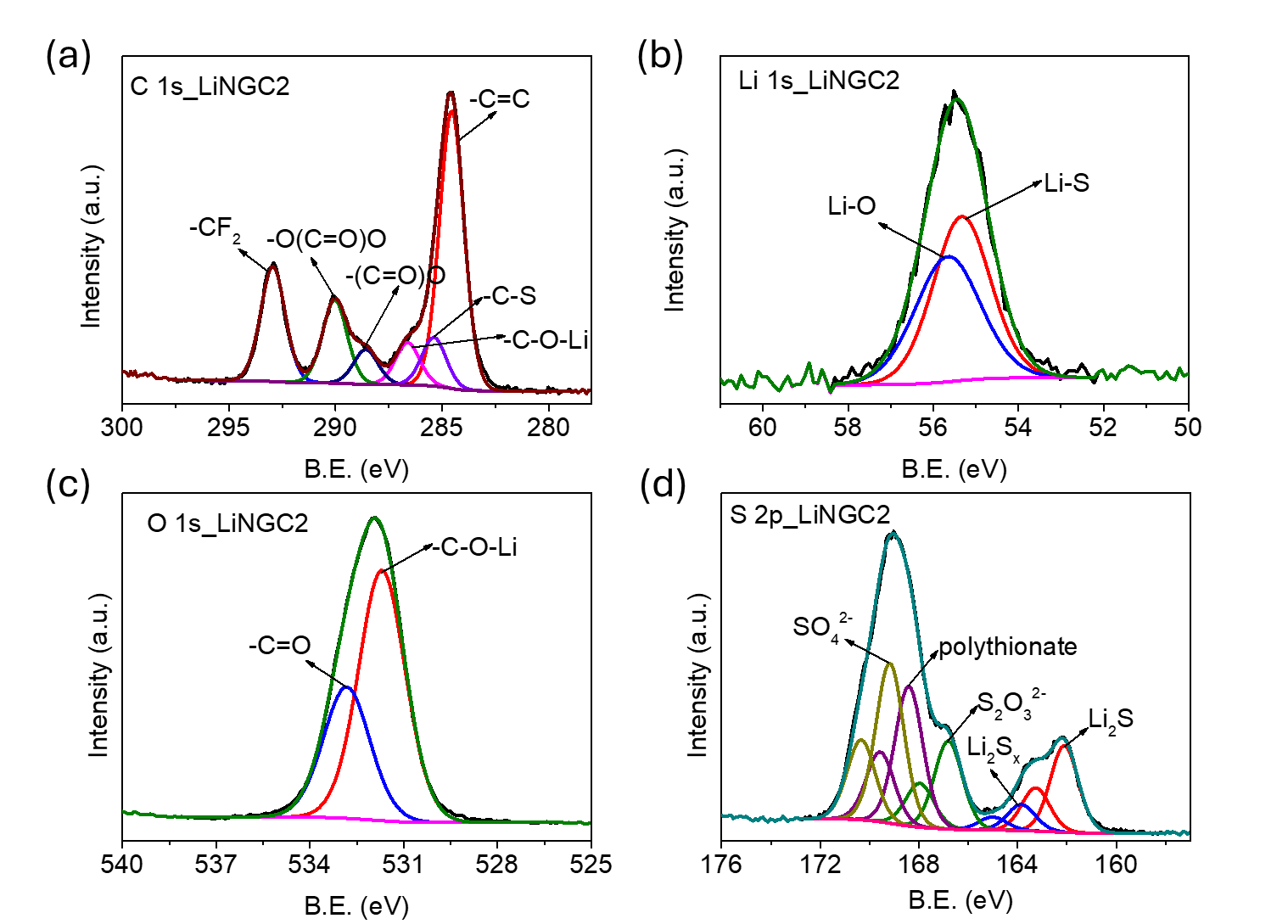


**Figure S46**: Post cycling deconvoluted XPS spectra of **NGC-2/S** electrodes: a) C1s, b) Li1s, c) O1s, and d) S2p.


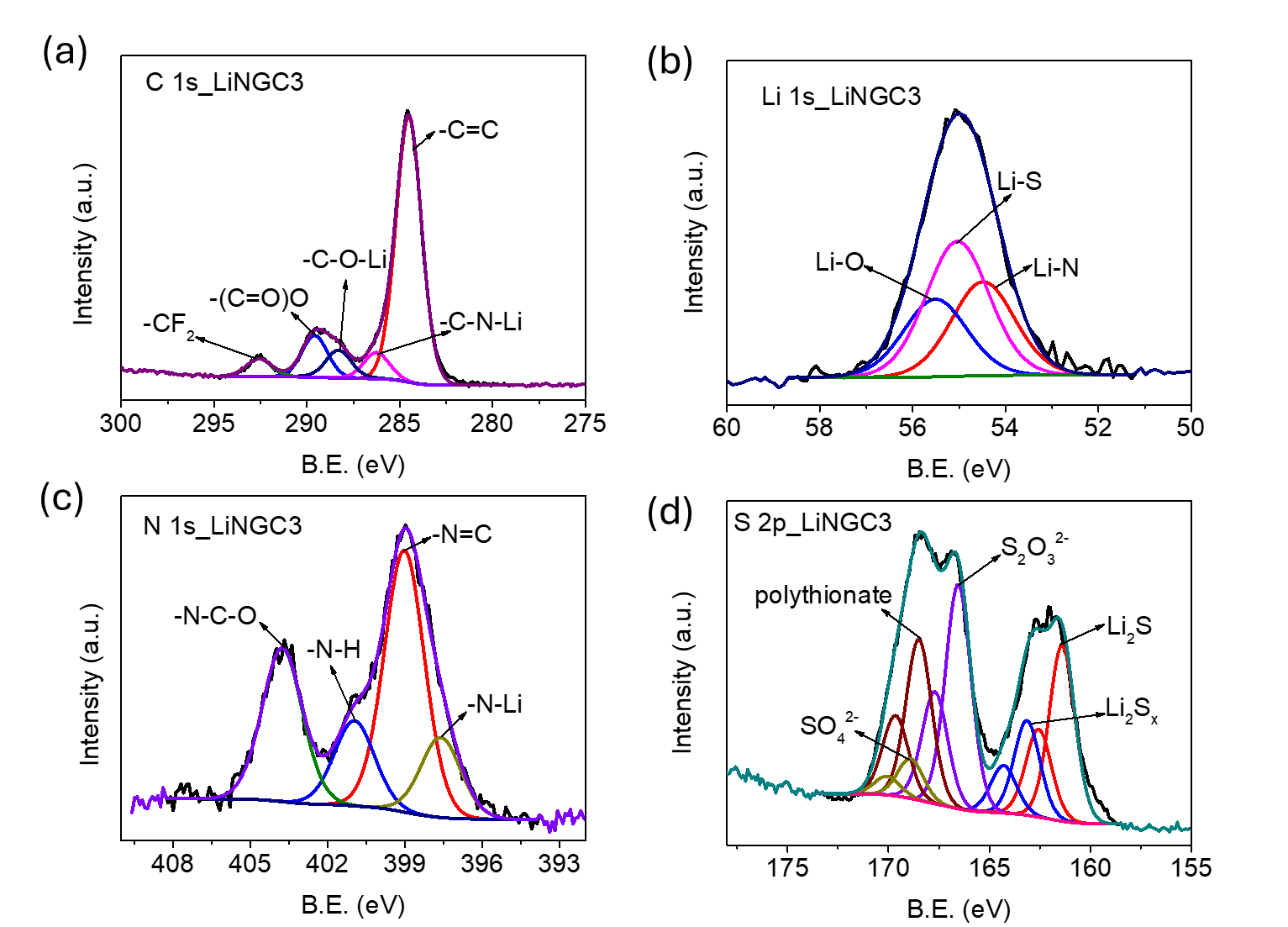


**Figure S47**: Post cycling deconvoluted XPS spectra of **NGC-3/S** electrodes: a) C1s, b) Li1s, c) N1s, and d) S2p.

**Figure S48**: Mechanisms for suppressing the shuttle effect of lithium polysulfides (LiPSs) in **NGC** covalent organic frameworks (COFs) containing pyridine and chalcone functionalities. Key mechanisms include (1) stabilization of LiPSs through Lewis acid-base interactions with chalcone carbonyl groups, (2) π-π (anion-π) interactions between nanographene-like polyaromatic cores and sulfur species, (3) pyridine-Li⁺ coordination to immobilize LiPSs, (4) synergistic adsorption and immobilization of LiPSs by chalcone and pyridine functionalities, and (5) facilitated Li₂S nucleation and deposition during discharge. Structural integrity and active site retention enhance cycling stability and overall battery performance.

**Table S7.** Comparison of the **NGC** COFs Li-S battery performance with state-of-the-art COF-based and inorganic sulfur host materials for LSBs.

| **COF** | **Sulfur**  **loading**  **(wt.%)** | **Capacity**  **(CD**  **/RC, mA h/g)** | **Cycling stability**  **(CR%/CN/CD)** | **Voltage range**  **(vs.Li/Li^+^)** | **Mass loading**  **(mg cm^-2^)** | **Reference** |
| --- | --- | --- | --- | --- | --- | --- |
| **NGC-1/S** | 87.8 | 0.5/804, 1/642 | 75/500/0.5  66/500/1 | 1.5-3 V | 1-3 | **This work** |
| **NGC-2/S** | 85.8 | 0.5/571, 1/517 | 63/500/0.5  71CR/500/1 | 1.5-3 V | 1-3 | **This work** |
| **NGC-3/S** | 87.4 | 0.5/929, 1/839 | 76/500/0.5  81/500/1 | 1.5-3 V | 1-4 | **This work** |
| Thiazole-linked THZ-DMTD | 82 | 1/642 | 78.9/200 | 1.6 -2.8 V | 4:1 by mass (COF: Sulfur) | 13 |
| 3D-flu-COF | 70 | 0.2/1249 | 93/100/0.5 75/1000/5.0 | 1.7−2.8 V | 0.8-1.2 | 14 |
| COF-1 | 40 | 0.2/1628, 0.5/1032 | 929/100/0.2 | 3.0–1.5 V | -- | 15 |
| COF-F | 60 | 0.1/962 | 645/100/0.1  257/1000/1 | 1.7-2.8 V | 0.5 | 16 |
| Py-COF | 70 | 0.5/ 1145, 1/1064, 5/659 | 481.2/550/5.0 963.4/100/1.0 | 1.8 - 2.7 V | 0.8 -1.2 | 17 |
| TB-COF | 40 | 0.2/1390,1/670 | 663/800/1  945/250/0.2 | 1.5–3.0 V | 0.9 | 18 |
| TAPB-PDA-COF (S-P) | 60 | 0.2/1222, 0.4/779, 0.6/688, 1/658 | 705 / 210/0.2  274 /940/2 | 1.9–2.6 V | 0.6 | 19 |
| TPAS-TPB-COF | 71 | 0.2/1134, 1/857.6 | 521/500/1 | 1-3 V | -- | 20 |
| 3D-scu-COF-1 | 70 | 0.2/1035, 1/855, 5/713 | 941/100/0.5 | 1.7−2.8 V | 0.8-1.2 | 21 |
| 3D-scu-COF-2 | 68 | 0.2/1155,1/941, 5/757 | 1021 /100/0.5 | 1.7−2.8 V | 0.8-1.2 | 21 |
| USTB-27-Co | 70 | 0.1/1063, 5/644 | 543/500/1 | 1.7−2.8 V | 0.8-1.2 | 22 |
| S@TFPB-TAA | 58 | 0.1/1288, 2/537 | 69.3%/400/0.5 | 1.7−2.8 V | 0.2 | 23 |
| s@ TFPB-TAB | 61 | 0.1/1192,2/586 | 73.5%/400/0.5 | 1.7−2.8 V | 0.2 | 24 |
| Por-COF | 55 | 0.2/850, 1.0/670 | 633/200/0.5 | 1.8-2.7 V | 1.8 | 25 |
| COF-ETTA-ETTCA | 88.40 | 0.1/1617, 5/185 | 605 /528/0.5 | 1.8-2.8 V | 1.3 | 26 |
| CTF-1/S | 34 | 0.1/920, 0.2/848,1/541 | 762/50/0.1 | 1.1–3.0 V | -- | 27 |
| S-CTF-1 | 62 | 0.5/670, 0.1/607, 0.2/562  2.0/402 | 84/50/0.2  85.8/300/1 | 1.7–2.7 V | 0.5 | 28 |
| S/Azo-COF | 39 | 2.0/770 | 741/100/0.1 | 1.5-2.8 V | 0.6–0.8 | 29 |
| FCTF-S | 51 | 0.1/1296, 0.5/1131 | 833/150/0.5 | 1.7−2.8 V | 0.7 | 30 |
| COF-Tr-BA | 40 | 0.1/1400, 0.2/948, 0.5/841, 1/ 767, 2/714 | 627/200/0.5 | 2.03-2.29 V | 1-1.2 m | 31 |
| COF-PA-AI/S | 40 | 0.2/795.5, 0.5/711.2, 1/623, 2/ 518.4 | 665.3/200/0.5 | 1.7−2.8 V | 1.5 | 32 |
| S-COF-2 | 43 | 0.2/1293, 0.5/ 954, 1/846,2/ 756, 3/692 | 801/140/0.2 | 1.7-2.7 V | 1 | 33 |
| S@CTFO | 67 | 0.2/995,0.5/ 905,1/ 790, 2/ 780 | 512/300/1 | 1.7-2.8 V | 1.0−1.2 | 34 |
| PI-CONs | 73 -55 | 0.1/1330, 1.0/900 | 96/100/0.2 85/500/1 | 1.7-2.8 | -- | 35 |
| S@NiS4-TAPT | 77 | 1/753.1 | 500.2/400/1 | -1/2.5 -1 V | -- | 36 |
| cPpy-S-CTFs | 83 | 0.05/ 1203.4  0.2/800  5.0/350 | 86.7/500/0.5 | 1.8−2.7 V | 0.8 | 37 |
| S@CTF/TNS | 76 | 0.2/1307, 0.5/894, 1/791, 2/639 | 85.8/1000/ 1 | 1.5-2.8 | 1.5/5.6 | 38 |
| Polysulfie@TFPPy–ETTA–COF. | 64 | 0.2/698, 0.5/524, 0.8/414, 1/347 | 54/130/0.1 | 1.7-2.8 | -- | 39 |
| S-COF-V | 67 | 0.5/1045, 6/431,0.2/1324 Or 0.2/1150  5.0/500 | 416/1000/1  960/100/0.2 | 1.7-2.8 | 0.7 | 40 |
| SF-CTF | 86 | 0.1/878.7, 0.5/653.3, 1/603.25 | 435.1/300/2  520.1(81.6)/300/1 | 1.8-2.7 | 0.7 | 41 |

**CD**: current density, **RC**: reversible capacity (mA h g^-1^), **CN**: cycle number, **CR**: capacity retention %

**7. References**

1. G. Kresse, J. Hafner, *Phys. Rev. B: Condens. Matter Mater. Phys*., **1993**, *47*, 558.
2. J. P. Perdew, K. Burke, M. Ernzerhof, *Phys Rev Lett* **1996**, *77*, 3865.
3. P. E. Blöchl, *Phys Rev B: Condens. Matter Mater. Phys*., **1994**, *50*, 17953.
4. G. Kresse, D. Joubert, *Phys Rev B* **1999**, *59*, 1758.
5. S. Grimme, J. Antony, S. Ehrlich, H. Krieg, *J Chem Phys* **2010**, *132*, 154104.
6. G. Henkelman, B. P. Uberuaga, H. Jónsson, *J Chem Phys* **2000**, *113*, 9901.
7. J. S. Hummelshøj, A. C. Luntz, J. K. Nørskov, *J Chem Phys* **2013**, *138*, 034703.
8. C. Kang, Z. Zhang, V. Wee, A. K. Usadi, D. C. Calabro, L. S. Baugh, S. Wang, Y. Wang, D. Zhao, *J Am Chem Soc* **2020**, *142*, 12995.
9. T. Guo, Y. Ding, C. Xu, W. Bai, S. Pan, M. Liu, M. Bi, J. Sun, X. Ouyang, X. Wang, Y. Fu, J. Zhu, *Advanced Science* **2023**, *10*, 2302518.
10. P. Bhauriyal, T. Heine, *J Mater Chem A Mater* **2022**, *10*, 12400.
11. Z. Du, X. Chen, W. Hu, C. Chuang, S. Xie, A. Hu, W. Yan, X. Kong, X. Wu, H. Ji, L.-J. Wan, *J Am Chem Soc* **2019**, *141*, 3977.
12. G. Zhou, S. Zhao, T. Wang, S.-Z. Yang, B. Johannessen, H. Chen, C. Liu, Y. Ye, Y. Wu, Y. Peng, C. Liu, S. P. Jiang, Q. Zhang, Y. Cui, *Nano Lett* **2020**, *20*, 1252.
13. R. Yan, B. Mishra, M. Traxler, J. Roeser, N. Chaoui, B. Kumbhakar, J. Schmidt, S. Li, A. Thomas, P. Pachfule, *Angewandte Chemie International Edition* **2023**, *62*, e202302276.
14. W. Liu, K. Wang, X. Zhan, Z. Liu, X. Yang, Y. Jin, B. Yu, L. Gong, H. Wang, D. Qi, D. Yuan, J. Jiang, *J Am Chem Soc* **2023**, *145*, 8141.
15. Z. A. Ghazi, L. Zhu, H. Wang, A. Naeem, A. M. Khattak, B. Liang, N. A. Khan, Z. Wei, L. Li, Z. Tang, *Adv Energy Mater* **2016**, *6*, 1601250.
16. D.-G. Wang, N. Li, Y. Hu, S. Wan, M. Song, G. Yu, Y. Jin, W. Wei, K. Han, G.-C. Kuang, W. Zhang, *ACS Appl Mater Interfaces* **2018**, *10*, 42233.
17. Y. Meng, G. Lin, H. Ding, H. Liao, C. Wang, *J Mater Chem A Mater* **2018**, *6*, 17186.
18. Z. Xiao, L. Li, Y. Tang, Z. Cheng, H. Pan, D. Tian, R. Wang, *Energy Storage Mater* **2018**, *12*, 252.
19. J. Wang, L. Si, Q. Wei, X. Hong, L. Lin, X. Li, J. Chen, P. Wen, Y. Cai, *Journal of Energy Chemistry* **2019**, *28*, 54.
20. X. Song, D. Li, C. Luo, J. Hu, K. Xu, DOI: 10.2139/ssrn.4714086.
21. W. Liu, L. Gong, Z. Liu, Y. Jin, H. Pan, X. Yang, B. Yu, N. Li, D. Qi, K. Wang, H. Wang, J. Jiang, *J Am Chem Soc* **2022**, *144*, 17209.
22. X. Liu, X. Ding, T. Zheng, Y. Jin, H. Wang, X. Yang, B. Yu, J. Jiang, *ACS Appl Mater Interfaces* **2024**, *16*, 4741.
23. Z. Wang, X. Wu, S. Wei, Y. Xie, C.-Z. Lu, *Chemistry of Materials* **2024**, *36*, 2412.
24. H. Liao, H. Wang, H. Ding, X. Meng, H. Xu, B. Wang, X. Ai, C. Wang, *J Mater Chem A Mater* **2016**, *4*, 7416.
25. B.-Y. Lu, Z.-Q. Wang, F.-Z. Cui, J.-Y. Li, X.-H. Han, Q.-Y. Qi, D.-L. Ma, G.-F. Jiang, X.-X. Zeng, X. Zhao, *ACS Appl Mater Interfaces* **2020**, *12*, 34990.
26. Z. Xiao, L. Li, Y. Tang, Z. Cheng, H. Pan, D. Tian, R. Wang, *Energy Storage Mater* **2018**, *12*, 252.
27. H. Liao, H. Ding, B. Li, X. Ai, C. Wang, *J. Mater. Chem. A* **2014**, *2*, 8854–8858.
28. S. N. Talapaneni, T. H. Hwang, S. H. Je, O. Buyukcakir, J. W. Choi, A. Coskun, *Angew. Chemie - Int. Ed.* **2016**, *55*, 3106–3111.
29. X. Yang, B. Dong, H. Zhang, R. Ge, Y. Gao, H. Zhang, *RSC Adv.* **2015**, *5*, 86137–86143.
30. F. Xu, S. Yang, G. Jiang, Q. Ye, B. Wei, H. Wang, *ACS Appl. Mater. Interfaces* **2017**, *9*, 37731–37738.
31. Y. Liang, T. Xia, Z. Chang, W. Xie, Y. Li, C. Li, R. Fan, W. Wang, Z. Sui, Q. Chen, *Chem. Eng. J.* **2022**, *437*, 135314.
32. X. Liu, H. Chen, R. Wang, S. Zang, T. C. W. Mak, *Small* **2020**, *16*, 2002932.
33. J.-M. Chen, H. Duan, Y. Kong, B. Tian, G.-H. Ning, D. Li, *Energy & Fuels* **2022**, *36*, 5998–6004.
34. G. Gao, Y. Jia, H. Gao, W. Shi, J. Yu, Z. Yang, Z. Dong, Y. Zhao, *ACS Appl. Mater. Interfaces* **2021**, *13*, 50258–50269.
35. H. Duan, K. Li, M. Xie, J. M. Chen, H. G. Zhou, X. Wu, G. H. Ning, A. I. Cooper, D. Li, *J. Am. Chem. Soc.* **2021**, *143*, 19446–19453.
36. S. Lv, X. Ma, S. Ke, Y. Wang, T. Ma, S. Yuan, Z. Jin, J. L. Zuo, *J. Am. Chem. Soc.* **2024**, *146*, 9385–9394.
37. J. Kim, A. Elabd, S. Y. Chung, A. Coskun, J. W. Choi, *Chem. Mater.* **2020**, *32*, 4185–4193.
38. R. Meng, Q. Deng, C. Peng, B. Chen, K. Liao, L. Li, Z. Yang, D. Yang, L. Zheng, C. Zhang, J. Yang, *Nano Today* **2020**, *35*, 100991.
39. F. Xu, S. Yang, X. Chen, Q. Liu, H. Li, H. Wang, B. Wei, D. Jiang, *Chem. Sci.* **2019**, *10*, DOI 10.1039/c8sc04518f.
40. Q. Jiang, Y. Li, X. Zhao, P. Xiong, X. Yu, Y. Xu, L. Chen, *J. Mater. Chem. A* **2018**, *6*, 17977–17981.
41. S. H. Je, H. J. Kim, J. Kim, J. W. Choi, A. Coskun, *Adv. Funct. Mater.* **2017**, *27*, DOI 10.1002/adfm.201703947.
